# Characterization of Rare, Dormant, and Therapy-Resistant Cells in Acute Lymphoblastic Leukemia

**DOI:** 10.1016/j.ccell.2016.11.002

**Published:** 2016-12-12

**Authors:** Sarah Ebinger, Erbey Ziya Özdemir, Christoph Ziegenhain, Sebastian Tiedt, Catarina Castro Alves, Michaela Grunert, Michael Dworzak, Christoph Lutz, Virginia A. Turati, Tariq Enver, Hans-Peter Horny, Karl Sotlar, Swati Parekh, Karsten Spiekermann, Wolfgang Hiddemann, Aloys Schepers, Bernhard Polzer, Stefan Kirsch, Martin Hoffmann, Bettina Knapp, Jan Hasenauer, Heike Pfeifer, Renate Panzer-Grümayer, Wolfgang Enard, Olivier Gires, Irmela Jeremias

**Affiliations:** 1 Department of Gene Vectors, Helmholtz Zentrum München, German Center for Environmental Health (HMGU), 81377 Munich, Germany; 2 Anthropology and Human Genomics, Department Biology II, Faculty of Biology, Ludwig-Maximilians-Universität München, 82152 Martinsried, Germany; 3 Children's Cancer Research Institute and St. Anna Kinderspital, Department of Pediatrics, Medical University of Vienna, 1090 Vienna, Austria; 4 Department of Medicine V, University of Heidelberg, 69120 Heidelberg, Germany; 5 University College London Cancer Institute, London WC1E, UK; 6 Institute of Pathology, Ludwig-Maximilians-Universität München, 80337 Munich, Germany; 7 Department of Internal Medicine III, University Hospital Grosshadern, Ludwig-Maximilians-Universität München, 81377 Munich, Germany; 8 German Consortium for Translational Cancer Research (DKTK), Partnering Site, Munich, 81377 Munich, Germany; 9 Project Group Personalized Tumor Therapy, Fraunhofer Institute for Toxicology and Experimental Medicine ITEM, 93053 Regensburg, Germany; 10 Institute of Computational Biology, Helmholtz Zentrum München, German Center for Environmental Health (HMGU), 85764 Neuherberg, Germany; 11 Department of Mathematics, Technische Universität München (TUM), 85748 Munich, Germany; 12 Department of Medicine, Hematology and Oncology, Goethe University, 60590 Frankfurt, Germany; 13 Department of Otorhinolaryngology, Head and Neck Surgery, Grosshadern Medical Center, Ludwig-Maximilians-Universität München, 81377 Munich, Germany; 14 Department of Pediatrics, Dr. von Hauner Children’s Hospital, Ludwig Maximilians University München, 80337 Munich, Germany

**Keywords:** acute lymphoblastic leukemia, patient-derived xenograft (PDX) cells, dormant tumor cells, Cancer stem cells, treatment resistance, RNA single-cell sequencing, minimal residual disease (MRD), primary patients' ALL MRD cells

## Abstract

Tumor relapse is associated with dismal prognosis, but responsible biological principles remain incompletely understood. To isolate and characterize relapse-inducing cells, we used genetic engineering and proliferation-sensitive dyes in patient-derived xenografts of acute lymphoblastic leukemia (ALL). We identified a rare subpopulation that resembled relapse-inducing cells with combined properties of long-term dormancy, treatment resistance, and stemness. Single-cell and bulk expression profiling revealed their similarity to primary ALL cells isolated from pediatric and adult patients at minimal residual disease (MRD). Therapeutically adverse characteristics were reversible, as resistant, dormant cells became sensitive to treatment and started proliferating when dissociated from the in vivo environment. Our data suggest that ALL patients might profit from therapeutic strategies that release MRD cells from the niche.

## Highlights

• Patients' ALL cells growing in mice contain a rare unfavorable subpopulation
• Unfavorable cells display treatment resistance, dormancy, and stemness
• Unfavorable cells mimic patients' primary cells at minimal residual disease
• Retrieving unfavorable cells from their environment sensitizes them for treatment

## Graphical Abstract

**Figure:**
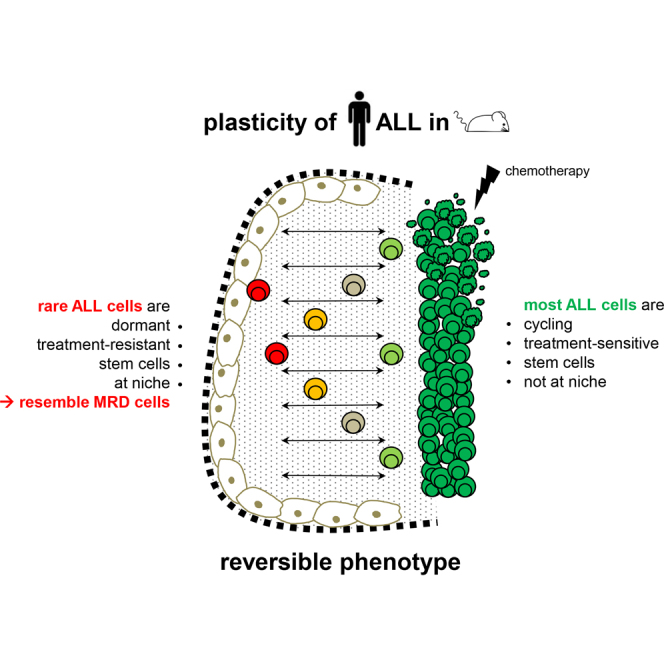

## Teaser

Ebinger et al. identify a rare subpopulation of acute lymphoblastic leukemia (ALL) cells that have the combined properties of long-term dormancy, treatment resistance, and leukemia initiation. RNA sequencing results show that these cells are similar to ALL cells isolated from patients at minimal residual disease.

## Significance

**After initially successful chemotherapy, relapse frequently jeopardizes the outcome of cancer patients. To improve the prognosis of ALL patients, treatment strategies that eliminate tumor cells at minimal residual disease (MRD) and prevent relapse are required. Toward a better understanding of the underlying biology, we established preclinical mouse models mimicking MRD and relapse in patients. Primary and surrogate MRD cells shared major similarities in expression profiles, demonstrating the suitability of our model. MRD cells revealed major functional plasticity in vivo and treatment resistance was reversible; MRD cells became sensitive toward treatment once released from their in vivo environment. Effective therapeutic strategies might aim at dissociating persistent cells from their protective niche to prevent relapse in ALL patients.**

## Introduction

Relapse represents a major threat for patients with cancer. After initially successful treatment, rare tumor cells might survive and re-initiate the malignant disease with dismal outcome. Acute lymphoblastic leukemia (ALL) is associated with poor prognosis in infants and adult patients and is the most frequent malignancy in children (Inaba et al., 2013). In many patients, the majority of ALL cells respond to chemotherapy but a minority display resistance, survive therapy, and cause relapse with poor outcome (Gokbuget et al., 2012).

Despite its clinical importance, basic biologic conditions underlying relapse remain partially elusive. For example, it is unclear whether relapse-inducing cells exist before onset of treatment or develop as result of therapy, and whether permanent or reversible characteristics determine relapse-inducing cells (Kunz et al., 2015). Of translational importance, understanding basic mechanisms opens perspectives for effective therapies to eradicate relapse-inducing cells.

Relapse-inducing cells, by their clinical definition, self-renew and give rise to entire tumors indicating tumor-initiating potential, a typical characteristic of cancer stem cells (Essers and Trumpp, 2010). In numerous tumor entities including acute myeloid leukemia, cancer stem cells were identified as a biologically distinct subpopulation that displays specific surface markers, has leukemia-inducing potential in mice, and gives rise to a hierarchy of descendant cells that lack such properties (Bonnet and Dick, 1997, Visvader and Lindeman, 2008). In ALL, however, many different subpopulations display stem cell properties; neither a stem cell hierarchy nor phenotypic markers defining stem cells could be identified (Kong et al., 2008, le Viseur et al., 2008, Rehe et al., 2013). Thus, up to now, stemness represents an insufficient criterion to define the subpopulation of relapse-inducing cells in ALL.

An additional feature of relapse-inducing cells is their treatment resistance, as, again by definition, they survive chemotherapy and eventually give rise to relapse with decreased chemosensitivity. Resistance against chemotherapy is closely related to dormancy as chemotherapy mainly targets proliferation-associated processes that are inactive in dormant cells (Clevers, 2011, Zhou et al., 2009). Dormant cells, by definition, do not divide or divide very slowly over prolonged periods of time, might survive chemotherapy, persist in minimal residual disease (MRD), and give rise to relapse (Schillert et al., 2013, Schrappe, 2014). Indeed, an increased frequency of non-dividing tumor cells has been described in patients after chemotherapy for defined subtypes of ALL (Lutz et al., 2013).

So far, technical obstacles have hampered characterizing phenotypic and functional features of relapse-inducing cells in ALL in detail. Established ALL cell lines represent inappropriate models as they display continuous proliferation. In patients, relapse-inducing cells are very rare and defining cell surface markers that reliably identify these rare ALL cells from the multiplicity of normal bone marrow cells remains intricate, at least in certain ALL subtypes (Hong et al., 2008, Ravandi et al., 2016). Moreover, primary ALL cells do not grow ex vivo, disabling their amplification in culture.

An attractive possibility to experimentally study patients' tumor cells in vivo is the patient-derived xenograft (PDX) model, which uses immuno-compromised mice to expand tumor cells from patients (Kamel-Reid et al., 1989). As shown previously, PDX ALL cells retain important characteristics of primary ALL cells (Castro Alves et al., 2012, Schmitz et al., 2011, Terziyska et al., 2012). While PDX models are mostly used for preclinical treatment trials (Gao et al., 2015, Townsend et al., 2016), we used them here to study relapse-inducing cells in ALL.

## Results

To characterize the challenging subpopulation of relapse-inducing cells in ALL, we used the individualized xenograft mouse model as a preclinical model, molecular cell marking as an unbiased approach, and in vivo dormancy as a functional benchmark. To mimic the heterogeneity of ALL, samples from nine different ALL patients were studied including children and adults, B cell precursor-ALL and T-ALL, first diagnosis, and relapse (Table S1).

### Molecular Marking Allows Unbiased, Sensitive Isolation of Rare PDX ALL Cells

**Figure 1:**
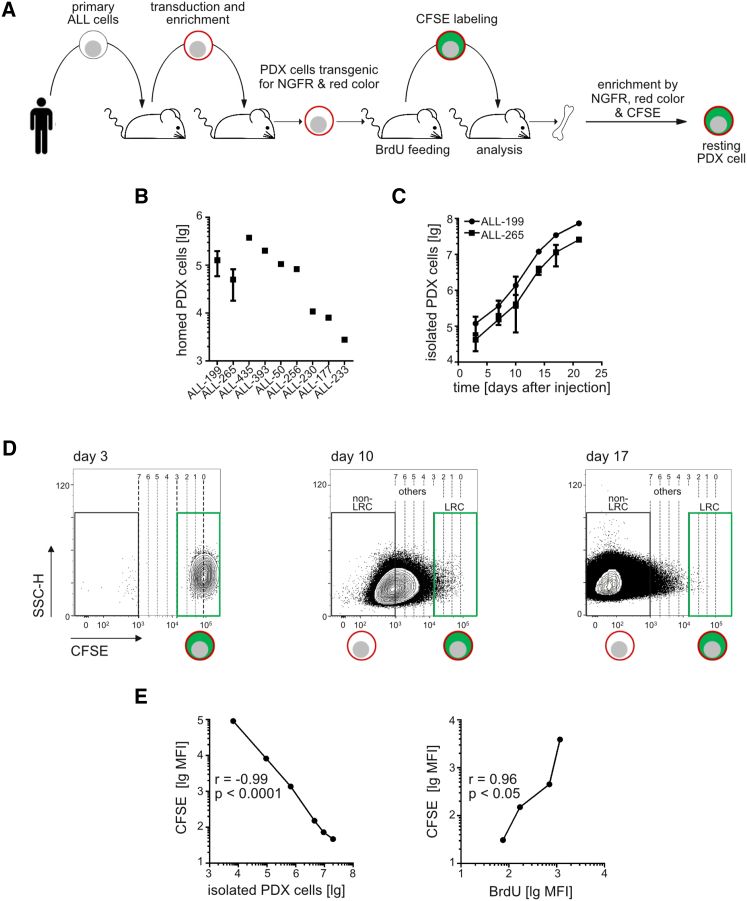
CFSE Staining Allows Reliable Monitoring of PDX ALL Growth in Mice (A) Experimental procedure of generating PDX ALL cells expressing several transgenes, staining with CFSE, and enriching rare transgenic, CFSE-stained PDX cells from mouse bone marrow. (B) Of each PDX sample, 10^7^ triple transgenic PDX cells were injected intravenously into mice and re-isolated from the bone marrow 3 days later; each dot represents data from one mouse, except that a mean of eight mice plus SE is shown for samples ALL-199 and ALL-265. (C) 10^7^ CFSE-stained PDX cells/mouse were injected and PDX cells were quantified in up to 11 mice per time point; shown is mean and SE. (D) Gating strategy defining LRC, non-LRC, and others. MFI of CFSE at the start of the experiment (3 days after cell injection) was divided by factor 2 to model bisections; upon no more than three bisections, cells were considered as LRC, upon more than seven bisections as non-LRC; intermediate cells were considered as others. (E) Similar experiment as in (C), except that the donor mouse was fed with BrdU in the last 7 days before cell harvesting. Each dot represents data from one mouse. See also Figure S1, Tables S1, and Table S2.

To study ALL growth starting very early after disease onset in the PDX mouse model, the technical challenge consisted in reliably enriching very low numbers of human ALL cells from mouse bone marrow. As expression levels of endogenous surface antigens across potentially relevant, but yet undefined, subpopulations are unknown, we used lentiviral transduction for unbiased molecular marking and in vivo imaging (Figure 1A).

PDX ALL cells were lentivirally transduced to express a luciferase for in vivo imaging (Terziyska et al., 2012), an artificial antigen (truncated nerve growth factor receptor [NGFR]) for magneto-activated cell sorting (Fehse et al., 1997) and a red fluorochrome for cell sorting by flow cytometry (Figures S1A and S1B). Transgenes allowed effective and reliable enrichment of minute numbers of PDX cells from mouse bone marrow in this two-step procedure. Quantification of PDX cells isolated with the magnetic-activated cell sorting (MACS)/fluorescence-activated cell sorting approach closely correlated with other methods monitoring leukemic proliferation, such as in vivo imaging and flow cytometry-based quantification of leukemia cells (Figure S1C). Quality controls showed that the procedure was highly efficient and reliable with minor cell loss (Table S2).

The procedure enabled addressing basic questions with translational potential in ALL biology. Homing capacity of PDX cells to mouse bone marrow differed by more than two orders of magnitude between the nine samples studied (Figure 1B). Homing efficiency decreased significantly when smaller cell numbers were injected (Figure S1D). These data argue in favor of sample-specific characteristics determining homing, and against the presence of a preformed, fixed number of leukemia homing sites within the niche. Spontaneous growth of PDX ALL cells in mouse bone marrow was logarithmic over the first 2 weeks of in vivo growth (Figures 1C and S1C). Growth slowed down thereafter and as early as at 10% blasts in bone marrow, when space restriction appears unlikely to be causative. Model selection indicated overall logistic growth which is typical for insufficient nutrient supply (Figure S1E). Thus, PDX ALL cells show sample-specific homing followed by logistic growth in mouse bone marrow.

### CFSE Staining Allows Reliable Monitoring of PDX ALL Growth in Mice

Proliferation-dependent dyes such as bromodeoxyuridine (BrdU) and carboxyfluorescein diacetate succinimidyl ester (CFSE) remain stable in mice over several months, enabling the characterization of a heterogeneous growth pattern in normal hematopoiesis (Takizawa et al., 2011). We adapted the use of these dyes in PDX tumor models. As BrdU staining requires the permeabilization and destroying of cells, fluorescent CFSE was mainly used as it allows flow cytometric enrichment of living cells for functional experiments including re-transplantation. Loss of CFSE was used to distinguish subpopulations of slowly and rapidly growing cells (Figures 1D and S1F) that were called label-retaining cells (LRC) and non-label-retaining cells (non-LRC), respectively (Takizawa et al., 2011). LRC were defined as those cells that had undergone at most three CFSE bisections resembling cell divisions (see the Supplemental Experimental Procedures for details). Loss of CFSE tightly correlated with increase in PDX cell numbers and loss of BrdU (Figures 1E and S1G) and confirmed that PDX ALL cells grow in vivo, but not ex vivo (Figure S1H). Thus, CFSE staining represents a reliable approach to monitor proliferation of PDX ALL cells in mice.

### A Rare, Long-Term Dormant Subpopulation Exists in ALL PDX Cells

**Figure 2:**
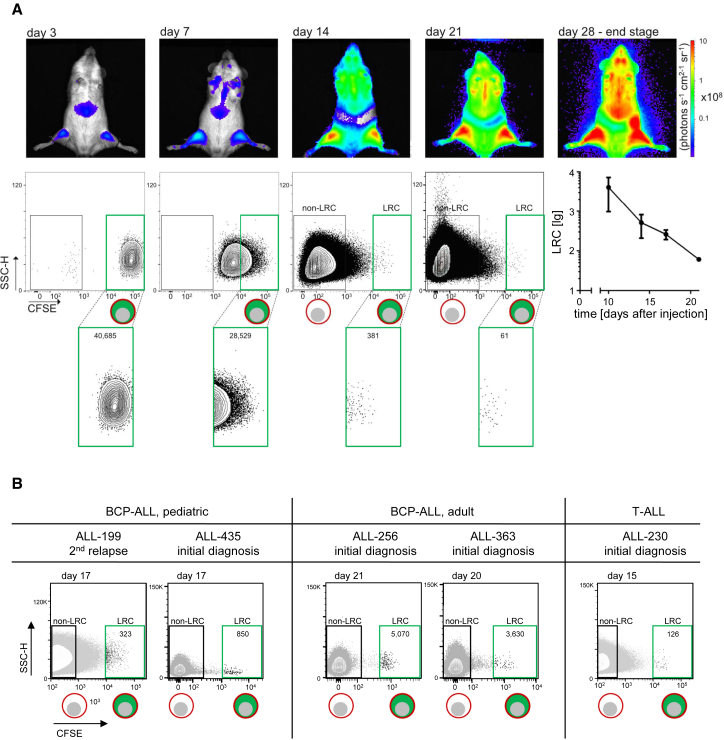
A Rare, Long-Term Dormant Subpopulation Exists in ALL PDX Cells (A) 10^7^ CFSE-stained PDX ALL-265 cells were injected into each of six mice; bioluminescence in vivo imaging was performed prior to quantifying LRC in one mouse per time point; LRC numbers are indicated and summarized in the line graph as a mean of up to ten mice ± SE. (B) Identification of LRC in PDX cells from all different ALL patients. Experiments were performed as in (A). See also Figure S2.

Importantly, CFSE staining disclosed the existence of a rare fraction of PDX ALL cells that hardly divided over prolonged periods of time (Figure 2A). LRC, by definition, had undergone no more than three cell divisions within 21 days, during which the leukemia burden had risen by several orders of magnitude so that mice would succumb to leukemia within a few days. In all nine PDX ALL samples studied, LRC were identified after prolonged periods of leukemic growth; (Figures 2B and S2A).

Thus, similarly to normal hematopoiesis (Trumpp et al., 2010), PDX ALL contains a rare subpopulation of LRC. LRC might resemble the dormant tumor cells described in ALL patients (Figure S2B) (Lutz et al., 2013). As an advantage over work with primary cells, our preclinical approach allows repetitive work on pure, vivid LRC, which gave us the chance to functionally and phenotypically characterize this interesting population.

### LRC Localize to the Endosteum, but Are Not Enriched for Stem Cells

**Figure 3:**
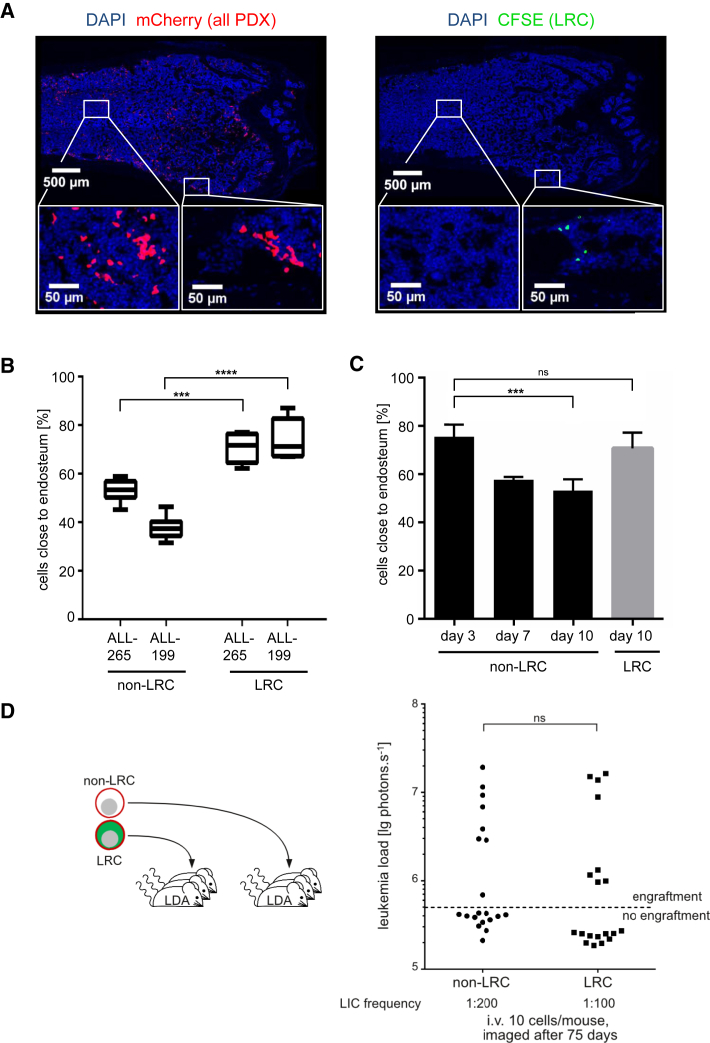
LRC Localize to the Endosteum, but Are Not Enriched for Stem Cells (A) Immunohistochemistry of consecutive mouse bone marrow femur sections 10 days after injection of CFSE-stained PDX ALL-265 cells; mCherry (red; left panel) indicates all PDX cells, CFSE (green; right panel) indicates LRC. (B) All sections from day 10 were quantified defining the endosteal region as less than 100 μm from bone matrix; shown is the median with upper/lower quartile and maximum/minimum of two to three sections from two femurs in two mice per data point; ^∗∗∗^p < 0.001, ^∗∗∗∗^p < 0.0001 by two-tailed unpaired t test. (C) Kinetic for ALL-265 as mean ± SE; ^∗∗∗^p < 0.01 by two-tailed unpaired t test. (D) Ten LRC or non-LRC were injected into each of 39 mice and engraftment was determined by in vivo imaging at day 75; each dot represents one mouse; dashed line represents detection threshold (5 × 10^5^ photons s^−1^); ns: not significant as determined by two-tailed unpaired t test. See also Figure S3 and Table S3.

Both normal hematopoietic stem cells and leukemia stem cells were reported to preferentially localize close to the endosteum, where a supportive niche might exist (Morrison and Spradling, 2008). We also found that LRCs preferentially localized close to the endosteum (Figures 3A–3C and S3), suggesting that they might use the same niche as normal hematopoietic stem cells and cancer stem cells.

We therefore asked whether LRC might resemble cancer stem cells. To compare leukemia-initiating potential between LRC and non-LRC, we performed limiting dilution transplantation assays and monitored engraftment by bioluminescence in a total of 83 mice (Table S3). To our surprise, we found highly similar stem cell frequencies in LRC and non-LRC and similar engraftment rates after transplantation of, e.g., ten cells per mouse (Figure 3D). The 95% confidence interval of the estimated frequency of leukemia-inducing cells ranged between 1/19 and 1/84 cells for LRC and between 1/40 and 1/179 cells in non-LRC of ALL-265 (Table S3). Similar findings were obtained for ALL-199 (Table S3). Thus, although only LRC display typical characteristics of stem cells such as reduced proliferation rate and localization close to the endosteum, LRC and non-LRC exhibited similar leukemia-initiating potential.

### LRC Survive Systemic Drug Treatment In Vivo

**Figure 4:**
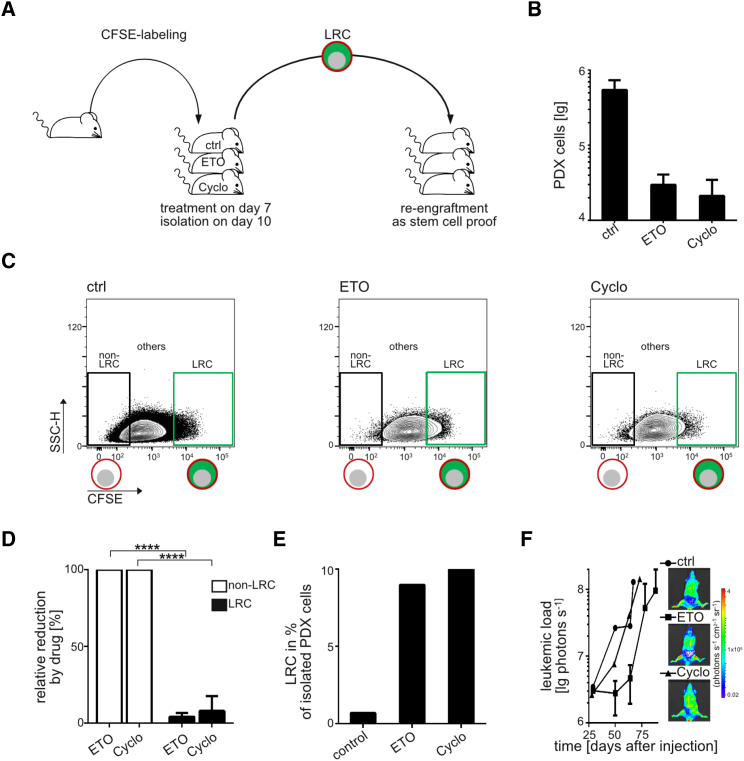
LRC Survive Systemic Drug Treatment In Vivo (A) Each mouse was injected with 10^7^ CFSE-stained ALL-265 PDX cells and treated with buffer, etoposide (ETO, 50 mg/kg, intraperitoneally [i.p.]), or cyclophosphamide (Cyclo, 150 mg/kg, i.p.) on day 7. Mice were euthanized on day 10; LRC were analyzed and re-transplanted into secondary recipients. (B) Living PDX cells from mice in (A) were quantified and presented as mean of each group (n = 4–5) ± SE. (C) Original data for one representative mouse per treatment. (D) Mean of all four to five mice per treatment, depicted as relative drug effect on LRC compared with non-LRC (100%) ± SE; ^∗∗∗∗^p < 0.0001 by two-tailed unpaired t test. (E) Mean relative proportion of LRC of total PDX cells. (F) LRC isolated were re-transplanted and mice monitored by in vivo imaging; mean of each group (n = 1–2) ± SE. See also Figure S4.

Dormant cells are known for their resistance against drug treatment, complicating elimination by anti-cancer therapy (Essers and Trumpp, 2010). We compared in vivo drug response of LRC and non-LRC by transplanting CFSE-labeled PDX ALL cells, treating mice with systemic chemotherapy on day 7 and analyzing surviving LRC and non-LRC on day 10 (Figure 4A). Chemotherapy reduced the overall leukemic burden by over 90% (Figures 4B and S4A) and eradicated most non-LRC. As a prominent difference, most LRC survived chemotherapy so that LRC increased in relative proportions (Figures 4C–4E and S4B–S4D). A 10- to 100-fold less efficient elimination of LRC compared with non-LRC became obvious across all PDX ALL samples tested that were derived from either primary disease or relapse, suggesting that this phenomenon is not restricted to a certain disease stage. Treatment-surviving LRC harbored leukemia-initiating potential as they gave rise to leukemias upon re-transplantation at a kinetic similar to that of untreated LRC (Figures 4F and S4E).

Taken together, LRC share the most important functional features that impede the cure of cancer: (1) dormancy, (2) in vivo drug resistance, and (3) leukemia-initiating potential. LRC might thus serve as preclinical surrogate for relapse-inducing cells in ALL.

### Expression Profile of LRC Shows Distinct Changes to Non-LRC

**Figure 5:**
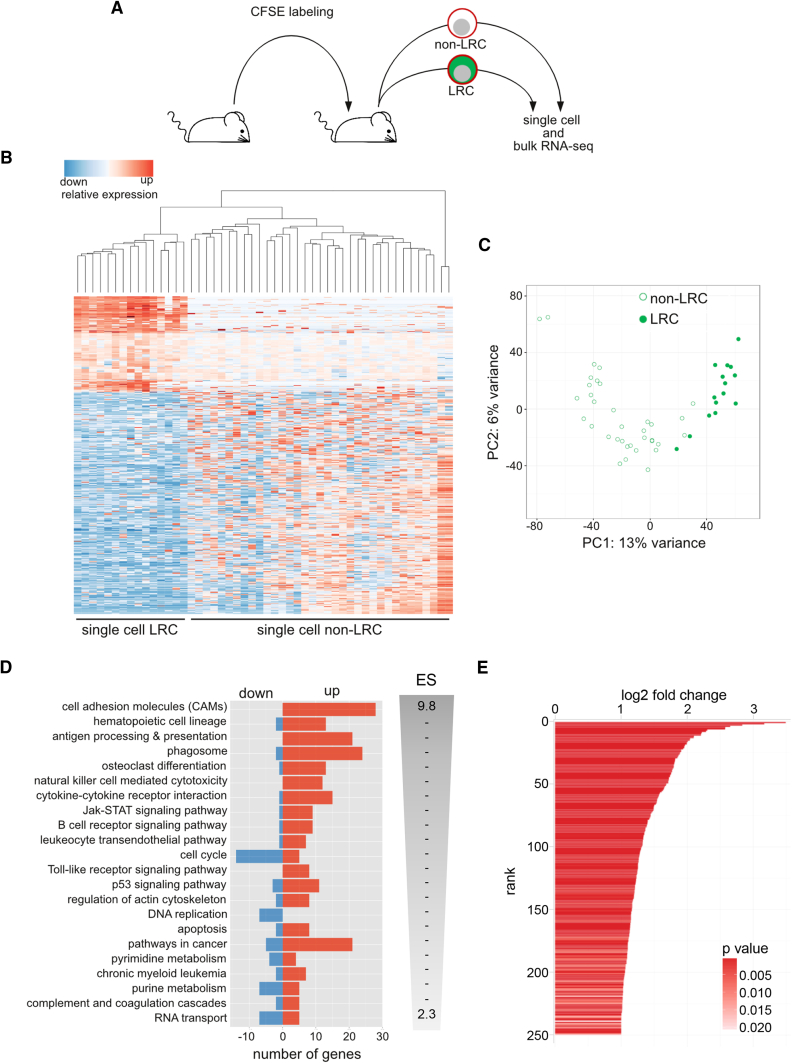
Expression Profile of LRC Shows Distinct Changes to Non-LRC (A) Fifteen days after transplantation, ALL-265 LRC or non-LRC were isolated and single-cell mRNA-seq was performed in 15 LRC and 35 non-LRC. (B) Hierarchical clustering and gene expression heatmap across the 500 most differentially expressed genes (false discovery rate [FDR] <0.01) in 15 LRC and 35 non-LRC single cells. Values are plotted relative to the average of non-LRC. (C) Principal component analysis of the 500 most variable genes in all 50 single cells. (D) Significantly enriched KEGG pathways (FDR <0.05) as determined by fixed network enrichment analysis (FNEA); bars show the number of significantly up- or downregulated genes in the corresponding pathway and are ordered according to the enrichment score (ES). (E) LRC signature genes (FDR < 0.05 and log2 fold-change >1) were derived from integrated bulk and single-cell RNA-seq analysis from six animals carrying either ALL-265 or ALL-199 and are shown ranked by fold-change and colored by significance. See also Figure S5, Tables S4, S5, and S6.

We then evaluated whether LRC adequately resemble challenging cells in patients. For a broad, unbiased comparison between LRC and non-LRC, RNA sequencing (RNA-seq) was performed on single cells and bulk populations (Figure 5A). Data from single cells correlated with data from bulk populations and different ALL PDX samples showed similar expression profiles (Figures S5A and S5B). Preliminary expression arrays on pools of 40 LRC and non-LRC showed mainly similar results (data not shown).

Single LRC differed consistently from single non-LRC as revealed by clustering differently expressed genes (Figures 5B and Table S4) and by a principle component analysis of the most variable genes (Figure 5C). Single LRC also had an overall reduced RNA content (Figure S5C), indicating a less active metabolism that is a prerequisite of dormant cells. We combined single-cell and bulk data of all six sample pairs to identify differently expressed genes (Table S5). Enrichment analysis revealed that genes expressed less in LRC were most strongly enriched in cell cycle and DNA replication and that genes more expressed in LRC were most strongly enriched in cell adhesion (Figures 5D, S5D, and Table S6). Hence, expression profiling of single cells and in bulk confirmed the quiescent state of LRC and an LRC signature of at least 2-fold differently expressed genes ranked by their significance (Figures 5E and Table S5) was used for further comparisons.

### LRC Resemble MRD Cells in the PDX Mouse Model

Relapse often results from treatment-resistant tumor cells that survive chemotherapy and persist at MRD. MRD cells contain a major fraction of dormant tumor cells (Lutz et al., 2013). Here, we hypothesized that LRC might represent surrogates for MRD cells.

**Figure 6:**
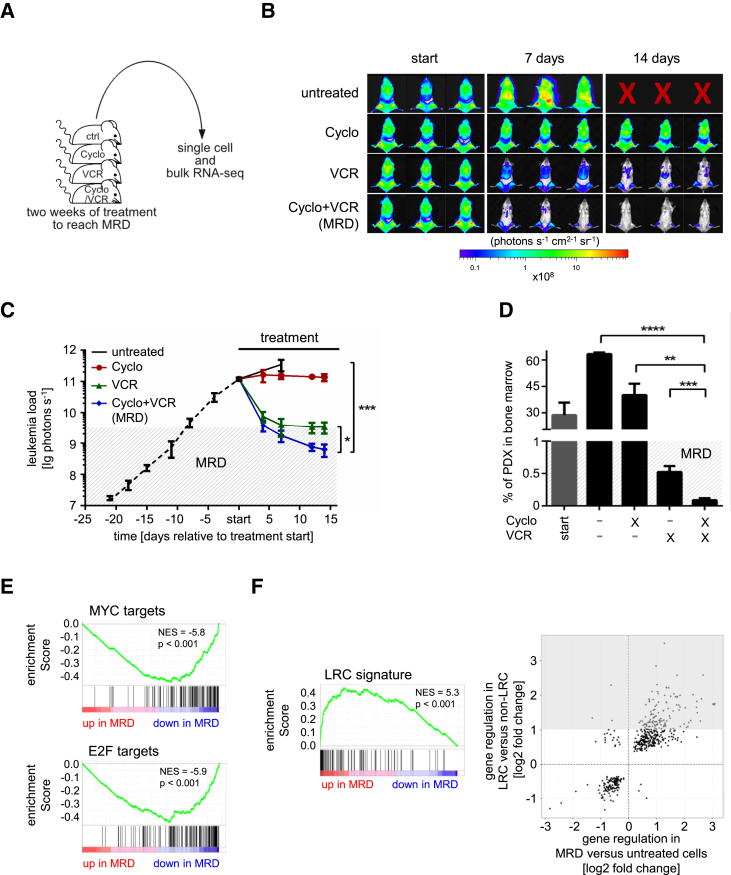
LRC Resemble MRD Cells in the PDX Mouse Model (A) 10^7^ ALL-199 cells were injected into 19 mice; when 30% of bone marrow cells were human, PDX cells were enriched from five mice and used as untreated control samples; cells of one mouse were subjected to single-cell sequencing; the remaining mice received buffer, vincristine (VCR, 0.25 mg/kg; n = 5), cyclophosphamide (Cyclo, 100 mg/kg; n = 3), or a combination thereof (VCR + Cyclo; n = 6) weekly for 2 weeks; when VCR + Cyclo combination treatment had reduced tumor burden to MRD (<1% human cells in bone marrow), PDX cells were enriched and cells of one VCR + Cyclo mouse were subjected to single cell mRNA-seq. (B) In vivo imaging data of three representative mice per group. (C) Mean of each group ± SE; ^∗^p < 0.05, ^∗∗∗^p < 0.001 by two-tailed unpaired t test; mice receiving buffer had to be euthanized after 1 week of treatment due to end-stage leukemia. (D) Percentage of PDX ALL cells in mouse bone marrow as determined by flow cytometry postmortem as mean ± SE; ^∗∗^p < 0.01, ^∗∗∗^p < 0.001, ^∗∗∗∗^p < 0.0001 by two-tailed unpaired t test. (E) MRD cells show reduced expression of MYC- and E2F-target genes in gene set enrichment analysis (GSEA) (Liberzon et al., 2015). (F) GSEA was performed comparing LRC signature with transcriptomes of MRD versus untreated cells (mean of data for ALL-199; left panel). Scatterplot of fold-changes for genes differentially expressed (FDR < 0.05) between both LRC versus non-LRC and MRD versus untreated control cells; grey area indicates LRC signature (right panel). See also Figure S6.

To experimentally test this hypothesis, we established a preclinical model of MRD for ALL-265 and ALL-199. When untreated control samples were harvested at advanced leukemia, they contained a leukemic burden of ∼30% human blasts in mouse bone marrow, mimicking the situation at diagnosis. Remaining mice received a systemic treatment with conventional chemotherapeutic drugs over 2–3 weeks (Figure 6A), which needs careful dosing as supportive therapy is mainly unfeasible in mice. A combination treatment of vincristine and cyclophosphamide reduced tumor burden substantially according to in vivo imaging (Figures 6B, 6C, and S6A). Postmortem analysis revealed that chemotherapy had reduced leukemic burden by more than two orders of magnitude to ∼0.1% leukemia cells in bone marrow. This resembled not only complete morphologic, but also complete molecular remission criteria (Figures 6D and S6B). MRD cells revealed relapse-inducing potential as they re-grew in mice when treatment was stopped (Figure S6C).

MRD cells were isolated from mouse bone marrow using expressed transgenes as above, and RNA sequencing of single cells and bulk samples was performed. Resulting transcriptomes showed marked differences between MRD and untreated control cells (Figure S6D). Enrichment analysis revealed significantly reduced expression of MYC and E2F target genes in MRD compared with untreated cells. Genes expressed less in MRD cells were most strongly enriched in cell cycle and DNA replication, while genes expressed more in MRD cells were most strongly enriched in cell adhesion (Figures 6E and S6E). This suggests a dormant phenotype of MRD cells similar to the dormant phenotype seen in LRC (Figure 5D). KEGG pathway analysis highlighted that MRD cells were of dormant nature and expressed increased adhesion molecules (Figure S6E). Indeed, single MRD cells clustered together with single LRC in a principal component analysis separated from non-LRC and cells from untreated mice (Figure S6F). Accordingly, the LRC signature (Figure 5E and Table S5) was strongly enriched in MRD cells and genes in MRD and LRC cells were similarly regulated compared with their respective controls (Figure 6F). This suggests that LRC mimic MRD cells in our preclinical mouse model.

### LRC Resemble Primary MRD Cells from Patients

**Figure 7:**
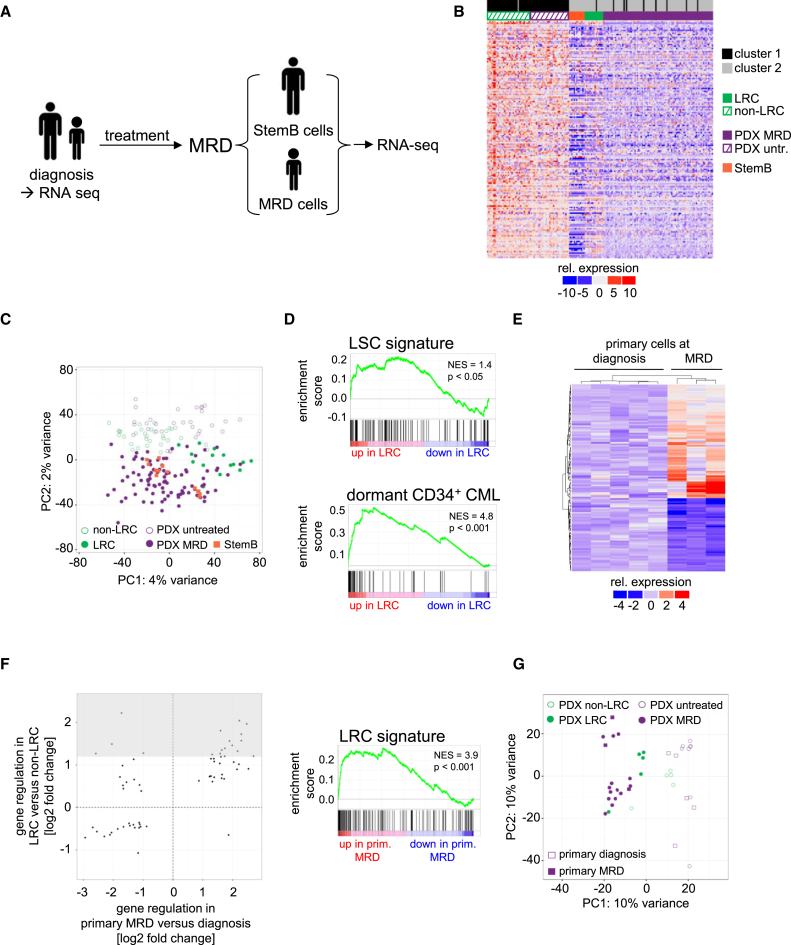
LRC Resemble Primary MRD Cells from Patients (A) Adult or pediatric ALL patients were treated according to GMALL-0703 or BFM-2009 protocols for 71 or 33 days, respectively; at MRD, the subgroup of StemB cells (in samples from adults) or all remaining ALL cells (in samples from children) were enriched out of normal bone marrow; cells at diagnosis and at MRD were subjected to RNA-seq. (B) K-means clustering of gene expression values of 167 highly differentially expressed genes (FDR < 0.001) of all data from single cells. (C) Principal-component analysis (PCA) of single cell transcriptomes using all shared expressed genes; each symbol indicates a single cell. (D) GSEA comparing the LRC signature with signatures of leukemia stem cells (Saito et al., 2010) and dormant CD34-positive chronic myeloid leukemia (CML) (Graham et al., 2007). (E) All genes differentially expressed (padj < 0.05) between primary samples from five children before onset of treatment to three matched MRD samples 33 days after onset of treatment. (F) Scatterplot of fold-changes for genes differentially expressed between both LRC versus non-LRC and primary MRD versus primary diagnostic cells, grey area indicates LRC signature (left panel); GSEA comparing the LRC signature with differentially expressed genes between primary MRD and primary diagnostic cells (right panel). (G) PCA of bulk samples transcriptomes using all shared expressed genes; each symbol indicates a single sample. See also Figure S7, Tables S7, and S8.

To relate these findings to the clinical situation, expression profiles from primary tumor cells from five children and two adults with B cell precursor (BCP) ALL were profiled at diagnosis and at MRD (Figure 7A and Table S7). Children and adults were treated according to the BFM-2009 and GMALL-0703 protocols, respectively, and MRD cells were enriched by flow cytometry at days 33 and 71 of treatment, respectively. In adults, we chose BCR-ABL-positive ALL and enriched the subpopulation of StemB cells at MRD, as Lutz et al. (2013) had shown that these cells exhibit a dormant phenotype. As dormancy in StemB cells might have persisted for a long period during treatment in patients, LRC might especially resemble StemB cells at MRD. We could obtain single-cell transcriptomes from one patient and one bulk transcriptome from another patient. K-means clustering and principal component analysis revealed that single StemB cells clustered together with single LRC and MRD cells, while single non-LRC clustered together with single untreated control cells (Figures 7B and 7C). The bulk StemB sample was distinct from diagnostic tumor cells of untreated adult patients with BCR-ABL-positive ALL (Figure S7A). Although limited by small cell and sample numbers, the data indicate that LRC resemble the dormant subpopulation of StemB cells in adult ALL patients at MRD.

This is also supported when comparing the LRC profiles with further published transcriptomes. Genes differently expressed in CD34-positive chronic myeloid leukemia cells (Graham et al., 2007), in leukemia stem cells (Saito et al., 2010), in hematopoietic stem cells (Eppert et al., 2011, Georgantas et al., 2004), as well as in pediatric ALL cells with high risk of relapse (Kang et al., 2010) were all significantly enriched in LRC versus non-LRC cells (Figures 7D, S7B, and S7C).

To further analyze the similarity of LRC to MRD cells from patients, we generated bulk transcriptomes of primary samples from five children with BCP-ALL before the onset of treatment and three matched MRD samples collected 33 days after the onset of treatment. Expression profiles differed significantly between diagnosis and MRD (Figure 7E and Table S8) and MRD cells regulated genes in the same direction as LRC compared with their respective controls, as revealed by a significant overlap of up- and downregulated genes (hypergeometric test, p = 1.9 × 10^−23^) and by a significant enrichment of the LRC signature (p < 0.001; Figure 7F). Finally, we combined these transcriptomes with all bulk samples isolated from the LRC and MRD mouse models and analyzed them unsupervised in a principal component analysis (Figure 7G). The first principal component separated all dormant and drug-resistant cells (PDX-LRC, PDX-MRD, and primary MRD) from all control cells (PDX-non-LRC, PDX untreated, and primary diagnosis).

In summary, we show that a distinct subpopulation of LRC exists in our ALL PDX model that combines the unfavorable characteristics of stemness, drug resistance, and dormancy. These LRC show high similarities to MRD cells in our mouse model and to MRD cells in ALL patients. Hence, LRC might represent preclinical surrogates for relapse-inducing cells in patients and could be used to develop therapeutic strategies to prevent relapse.

### Release from the Environment Induces Proliferation in LRC

As the first step toward therapies, we studied whether unfavorable drug resistance and dormancy represented permanent or reversible features in LRC. Dormancy and drug resistance might exist as genuine, constant biological characteristics of a special ALL subpopulation or as reversible functional phenotypes of putatively every ALL cell depending on the context.

**Figure 8:**
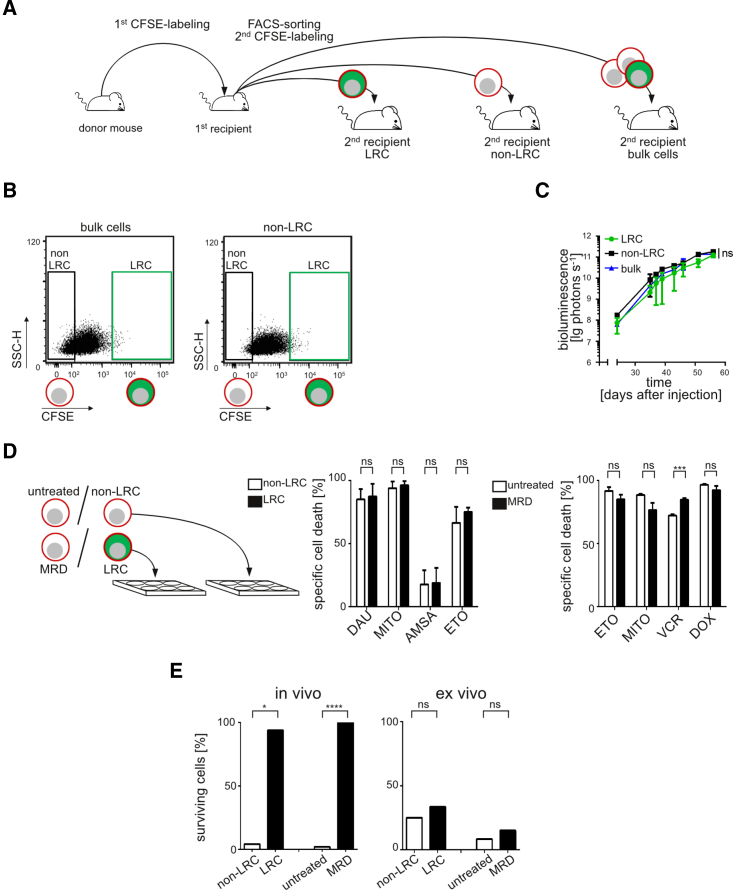
Release from the Environment Induces Proliferation in LRC and Sensitizes LRC and MRD Cells toward Drug Treatment (A) From a first recipient mouse carrying CFSE-stained ALL-199 cells, LRC, non-LRC, and bulk cells were obtained at day 10; bulk cells and non-LRC were re-labeled with CFSE, re-transplanted in second recipient mice at high numbers, and re-analyzed at day 10 using flow cytometry; bulk cells, LRC, and non-LRC were re-transplanted at low numbers into groups of mice and leukemia growth was monitored over time. (B) CFSE staining at day 10 in secondary recipient mice receiving high cell numbers. (C) Growth curve in secondary recipients; mean ± SE; ns, no statistical significance by Kruskal-Wallis test and Dunn's multiple comparison test. One out of two independent experiments is shown. (D) Fourteen days after transplantation, LRC or non-LRC were isolated and 500–800 cells treated ex vivo for 48 hr with daunorubicin (DAU; 250 nM), mitoxantrone (MITO; 675 nM), amsacrine (AMSA; 18 nM), or etoposide (ETO; 300 nM). Spontaneous cell death in the absence of cytotoxic drugs was 60%; a mean of eight data points from three independent experiments in triplicates or duplicates is shown for DAU and MITO and one experiment in triplicates is shown for AMSA and ETO. Four thousand untreated cells and MRD cells were treated ex vivo for 48 hr with 15 μM ETO, 450 μM MITO, 300 nM VCR, or 500 nM DOX. Cell death was measured by flow cytometry; spontaneous cell death in the absence of cytotoxic drugs was 33%; shown is one experiment in triplicate; mean ± SE; ns, not significant, ^∗∗∗^p < 0.001 by two-tailed unpaired t test. (E) Summary of ALL-265 data from Figure 4C (n = 5), S6 (n = 3), and 8D (n = 3); ns, not significant, ^∗^p < 0.05 and ^∗∗∗∗^p < 0.0001 by two-tailed unpaired t test.

To address this question, LRC and non-LRC were dissociated from their environment, isolated, and re-transplanted into recipient mice (Figures 8A and S8A). When non-LRC were re-stained with CFSE and re-transplanted at high numbers, they gave rise to an identical LRC population as re-transplanted bulk cells (Figures 8B and S8A); transplantation of high cell numbers of LRC was impossible, as only low numbers of LRC can be recovered from mice. When low cell numbers were re-transplanted, LRC, non-LRC, and bulk cells initiated identical leukemic growth in mice as monitored by bioluminescence in vivo imaging (Figures 8C and S8A). These data indicate that dormancy represents a reversible feature of LRC, as LRC lose their dormant nature once they are retrieved from their specific environment and transferred into a different surrounding.

### Release from the Environment Sensitizes LRC and MRD Cells for Drug Treatment

As dormancy emerged as a reversible phenotype, we asked whether drug resistance might be equally reversible. Isolated LRC and non-LRC or MRD and previously untreated cells from the PDX mouse model were treated ex vivo with common ALL chemotherapy drugs or drug controls. Here, the technical challenge lay in the very minor cell numbers of LRC and MRD that can be isolated from mice and used for ex vivo experiments (Figure S8B). Co-culture with feeder cells resembling bone marrow stroma reduced drug response in all samples, suggesting the influence of the bone marrow environment on drug resistance (Figures S8C–S8F) (Tesfai et al., 2012). Ex vivo, neither LRC nor MRD cells displayed increased drug resistance compared with their respective controls (Figures 8D and S8G).

Taken together, LRC and MRD cells showed a marked gain in drug sensitivity ex vivo compared with in vivo after isolation from the bone marrow environment. Both LRC and MRD cells lost their enhanced drug resistance, distinguishing them from non-LRC or untreated cells, once they were retrieved from their in vivo environment and cultured ex vivo (Figure 8E). Dormancy was reversible in LRC and drug resistance was reversible in both LRC and MRD cells. As LRC might represent surrogates for relapse-inducing cells in patients, our data suggest that the interaction between LRC and their environment represents an attractive therapeutic target for preventing relapse. Relapse-inducing cells might gain sensitivity toward treatment once mobilized from their in vivo environment.

## Discussion

The present work aimed at a better understanding of the cells that induce relapse in ALL and thereby limit prognosis of patients. We identified a rare, long-term dormant subpopulation termed LRC exhibiting the adverse characteristics of dormancy, in vivo drug resistance, and leukemia-initiating properties. LRC highly resemble primary MRD cells from adult and pediatric patients with ALL. MRD cells require preferential eradication by anti-leukemia treatment. LRC in preclinical models can now be used as surrogates for relapse-inducing cells in patients for developing therapies to prevent relapse. Upon removal from their in vivo environment, LRC lost dormancy and drug resistance, suggesting a reversible nature of adverse characteristics and an important role for the interaction between ALL and the environment. The data suggest that drug resistance and dormancy are linked and represent an acquired stem-like phenotype. Our data imply developing treatment approaches that dissociate ALL cells from their protective niche to sensitize them toward anti-leukemia treatment.

Here, we provide a preclinical tool to study dormant human ALL cells in vivo and show that long-term resting cells exist in ALL. This fact was previously unknown, as primary patients' samples allow quantifying non-cycling cells in a snapshot at a given moment, but fail to distinguish between short- and long-term resting cells (Lutz et al., 2013). As monitoring functionally defined cellular subpopulations such as LRC in longitudinal studies is still impossible in patients, our preclinical model enables the gaining of insights into ALL biology that cannot be obtained in patients: here the presence of long-term resting cells in ALL. Beyond its use in preclinical treatment trials, PDX models harbor major potential in basic research and enable unique insights into disease biology.

The emergence of relapse is a complex process involving genetic and non-genetic factors. Early relapse might be caused by a putatively pre-existing clone with additional mutations responsible for drug resistance, especially in adult patients. The genetic stability of most cases of ALL suggests that many relapses may not be mediated by mutational mechanisms. Late relapse might be caused by persisting, dormant tumor cells in the absence of additional mutations, and relapse cells often respond to the identical drugs used to treat the primary disease. LRC represent surrogates for late relapse and relapse in the absence of additional mutations, as often seen in children.

The fact that LRC exist might explain why ALL patients benefit from maintenance therapy, even in prognostically favorable, chemo-sensitive ALL subtypes. ALL patients are routinely treated with oral low-dose chemotherapy from end of intensive chemotherapy until, e.g., 2 years after diagnosis, and maintenance therapy improves patients' prognosis (Schrappe et al., 2000). Low-dose maintenance therapy might act by removing LRC-type ALL cells with relapse-inducing potential that remained quiescent over prolonged periods of time and turned on their cell cycle at late time points in the months following intensive chemotherapy.

Tumor cells often display both dormancy and drug resistance. It is unclear whether either dormancy or drug resistance is pivotal in respect to the other, so that dormancy is a consequence of resistance or vice versa (Blatter and Rottenberg, 2015). Our two complementary mouse models show that LRC were defined by their dormant nature and displayed drug resistance, while MRD cells were defined by their ability to survive drug treatment and displayed a dormant phenotype. Thus, both characteristics might be equally sufficient to determine each other and coincide interdependently.

Our study shows that ALL consists of functionally heterogeneous cells regarding proliferation rate and drug resistance, similar to the functional heterogeneity shown in other tumor entities (Kreso et al., 2013). As LRC did not substantially participate in proliferation during growth of leukemia over weeks, in our model LRC existed before onset of therapy and were not developed as a consequence of treatment. As both LRC and non-LRC contain similar amounts of stem cells, but show different sensitivity toward drug treatment in vivo, our data imply that stemness and drug resistance are not directly connected in ALL.

So how does a rare subpopulation acquire the three clinically challenging features dormancy, resistance, and stemness? LRC might represent a cell subpopulation with genuinely different biology harboring distinct intrinsic, constant characteristics, or being an LRC might represent a reversible, temporary, functional phenotype depending on circumstances. In the first case, LRC and non-LRC might be organized in a hierarchical way similar to the known stem cell hierarchy existing in many tumors including AML (Kreso and Dick, 2014). In the second case, ALL cells might mimic the phenotypic reversibility of normal hematopoiesis, where long-term dormant hematopoietic stem cells start cycling in response to stress for a defined period of time and turn back into dormancy later (Trumpp et al., 2010).

Our data favor the second scenario as LRC exhibit their specific characteristics as reversible, temporary, transient functional phenotypes. Re-transplantation experiments showed that formerly dormant LRC started proliferating as soon as they were dissociated from their in vivo environment and transferred into next recipient mice. Upon re-transplantation, LRC converted into non-LRC, while certain non-LRC converted into LRC. Both LRC and non-LRC thus harbored plasticity to switch between slow and rapid proliferation depending on the current context. This fact might explain the area of overlap between LRC and non-LRC detected in single-cell RNA sequencing.

Besides proliferation, drug resistance also proved to be a transient characteristic. Drug-treatment experiments showed that LRC lost their in vivo drug resistance upon ex vivo culture. The discrepancy between drug sensitivity ex vivo and in vivo might at least partly explain the limited predictability of ex vivo drug-screening tests for the outcome of cancer patients (Wilding and Bodmer, 2014). Thus, localization of LRC to the bone marrow niche influences both dormancy and drug resistance.

These insights have translational implications. For diagnostics, as LRC lose their clinically relevant characteristics upon release from their niche, rapid sample processing might be critical for reliable profiling, which represents a challenge in clinical routine (Bacher et al., 2010). Our data at least in part explain the limited power of in vitro assays using, e.g., proliferating cell lines, for studies on MRD cells or primary leukemia cells for drug testing in the absence of feeders. Most importantly for putative treatment strategies, the transient nature of the adverse characteristics of LRC suggests aiming at removing MRD cells from their protective environment to sensitize them toward treatment (Essers et al., 2009, Essers and Trumpp, 2010). The interaction between MRD cells and their bone marrow niche represents a promising target for therapeutic approaches to prevent relapse. Beyond the tumor cell itself, its interaction with the environment represents a suitable therapeutic target. As a caveat, a persistent therapeutic inhibition of the bone marrow niche might be required over prolonged periods of time, as in principle each and every remaining non-LRC ALL cell could convert into a drug-resistant LRC, as soon as it gets access to the protective niche.

At this point, we can only speculate which signals might determine whether an ALL cell behaves like an LRC or a non-LRC. In theory, external as well as internal factors or conditions might be influential; stimuli might be sent or received either stochastically or within a well-regulated process. As our studies were restricted to bone marrow, the bone marrow niche is a likely candidate for a regulatory function and requires investigatory work (Raaijmakers, 2011). Further research is required to address these important questions. Obvious candidates for therapeutic intervention are cell surface molecules expressed on LRC, the inhibition of which might release cells from their environment. Similarly, niche cells could be targeted to aim at reducing environmental support.

Our study shows that ALL growing in vivo contains a rare subpopulation of LRC that exhibits typical challenging adverse characteristics of relapse induction, which proved to be of a reversible nature. Our model might help to develop future anti-leukemia treatment strategies allowing the eradication of the precarious subpopulation of drug-resistant stem cells to prevent relapse and improve the prognosis of patients with ALL.

## Experimental Procedure

### Ethical Statements

Written informed consent was obtained from all patients and from parents/carers in the cases where the patients were minors. The study was performed in accordance with the ethical standards of the responsible committee on human experimentation (written approval by Ethikkommission des Klinikums der Ludwig-Maximilians-Universität München, Ethikkommission@med.unimuenchen.de, April 15, 2008, number 068-08) and with the Helsinki Declaration of 1975, as revised in 2000.

All animal trials were performed in accordance with the current ethical standards of the official committee on animal experimentation under the written approvals by Regierung von Oberbayern, poststelle@reg-ob.bayern.de, May 10, 2007 number 55.2-1-54-2531-2-07 and August 8, 2010 number 55.2-1-54-2531-95-10.

### Enriching and Quantifying PDX and LRC from Mouse Bone Marrow

PDX ALL cells were genetically engineered as described using lentiviruses (Terziyska et al., 2012, Vick et al., 2015) to express the transgenes’ truncated NGFR, a red fluorochrome, and luciferase; cells were stained with BrdU and/or CFSE before re-transplantation of fresh cells into mice.

For determining the fraction of dormant PDX ALL cells, mouse bone marrow was harvested from numerous bones and enriched for human PDX ALL cells using NGFR for MACS and the red fluorochrome for flow cytometry cell sorting. LRC were discriminated from non-LRC using CFSE staining as shown in Figure 1D. CFSE mean fluorescence intensity (MFI) was measured at day 3 after injection, when bleaching had ceased, and defined cells before the onset of proliferation (“0 divisions”). Day 3 CFSE MFI was divided by factor 2 to calculate CFSE bisections mimicking cell divisions. Seven CFSE MFI bisections or more were defined as entire loss of the CFSE signal characterizing non-LRC. The LRC gate was set to include all cells harboring high CFSE signal of below three bisections of the maximum CFSE MFI (Schillert et al., 2013) (Figure 1D).

### PDX Single-Cell RNA-Seq Library Construction

Single cells were isolated at 4°C and processed on the Fluidigm C1 platform. In brief, 500 cells were loaded on the 10–17 μm mRNA-seq IFC (Fluidigm) with External RNA Controls Consortium spike-in controls. Cell lysis, reverse transcription, and pre-amplification of cDNA was done on-chip using the SMARTer Ultra Low RNA Kit for C1 (Clontech). Harvested cDNA libraries of the samples (2.5 μL) were used as input for tagmentation with the Nextera XT Sample Preparation Kit (Illumina) at half the volume of Illumina's protocol. Barcoding PCR was performed for 12 cycles. Equal amounts of libraries were pooled.

### RNA-Seq

Single-cell Smart-seq and bulk Smart-seq2 libraries were sequenced at 1 × 50 bases on an Illumina HiSeq1500. SCRB-seq and UMI-seq libraries were sequenced paired-end with 16 cycles on the first read to decode sample barcodes and unique molecular identifiers and 50 cycles on the second read into the cDNA fragment.

## Author Contributions

S.E. and E.Z.Ö. planned, performed, and analyzed the experiments and designed the data presentation; C.Z., E.Z.Ö., S.P., and W.E. generated and analyzed RNA-seq data, W.E. participated in writing the manuscript; S.T. and C.C.A. established the mouse model, first detected LRC, and started their characterization; M.G. started establishing the MRD PDX model; A.S. guided the work of E.Z.Ö.; M.D. performed enrichment of pediatric MRD cells provided by R.P.G.; C.L., V.A.T., and T.E. performed enrichment of adult StemB cells provided by H.P.; H.P.H. and K.So. performed immunohistochemistry of primary bone marrow biopsies; K.Sp. and W.H. provided primary adult samples of Figure S2; B.P., S.K., M.H., and B.K. performed and analyzed gene expression array data; J.H. performed the mathematical analysis of Figure S1E; O.G. participated in designing the experiments, guiding the study, and writing the manuscript; I.J. initiated and guided the study and wrote the manuscript.

## Accession Numbers

RNA-seq data reported in this paper have been deposited in the NCBI's GEO database and are accessible through the GEO Series accession number GEO: GSE83142.

## Supplemental Information

Document S1. Supplemental Experimental Procedures, Figures S1–S8, and Tables S1–S3, S7, and S8

Table S4. List of the 500 Most Differentially Expressed Genes between LRC and Non-LRC in Single-Cell RNA-Seq of ALL-265, Related to Figure 5

Table S5. Integrated LRC Signature, Related to Figure 5

Table S6. KEGG Pathways Enriched with LRC versus Non-LRC Differentially Expressed Genes in Combined Analysis, Related to Figure 5

Table S8. List of the Most Significantly Differentially Expressed Genes between Primary Samples from Five Primary ALL Diagnosis and Three MRD Samples after 33 Days of Treatment, Related to Figure 7

Document S2. Article plus Supplemental Information
