# Supplementary material for: Characterization of Rare, Dormant, and Therapy-Resistant Cells in Acute Lymphoblastic Leukemia
Source: Cancer Cell. 2016 Dec 12;30(6):849–62. doi: 10.1016/j.ccell.2016.11.002 (PMC5156313; doi:10.1016/j.ccell.2016.11.002)
Supplement: Document S1. Supplemental Experimental Procedures, Figures S1–S8, and Tables S1–S3, S7, and S8 [file mmc1.pdf]

Cancer Cell, Volume *30*

Supplemental Information

Characterization of Rare, Dormant, and Therapy-Resistant Cells in Acute Lymphoblastic Leukemia

Sarah Ebinger, Erbey Ziya Özdemir, Christoph Ziegenhain, Sebastian Tiedt, Catarina Castro Alves, Michaela Grunert, Michael Dworzak, Christoph Lutz, Virginia A. Turati, Tariq Enver, Hans-Peter Horny, Karl Sotlar, Swati Parekh, Karsten Spiekermann, Wolfgang Hiddemann, Aloys Schepers, Bernhard Polzer, Stefan Kirsch, Martin Hoffmann, Bettina Knapp, Jan Hasenauer, Heike Pfeifer, Renate Panzer-Grümayer, Wolfgang Enard, Olivier Gires, and Irmela Jeremias

Supplemental Data

A

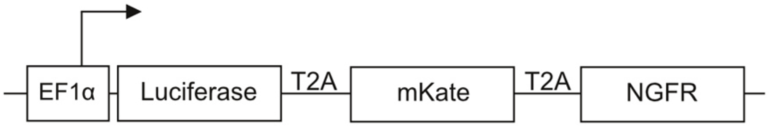

B

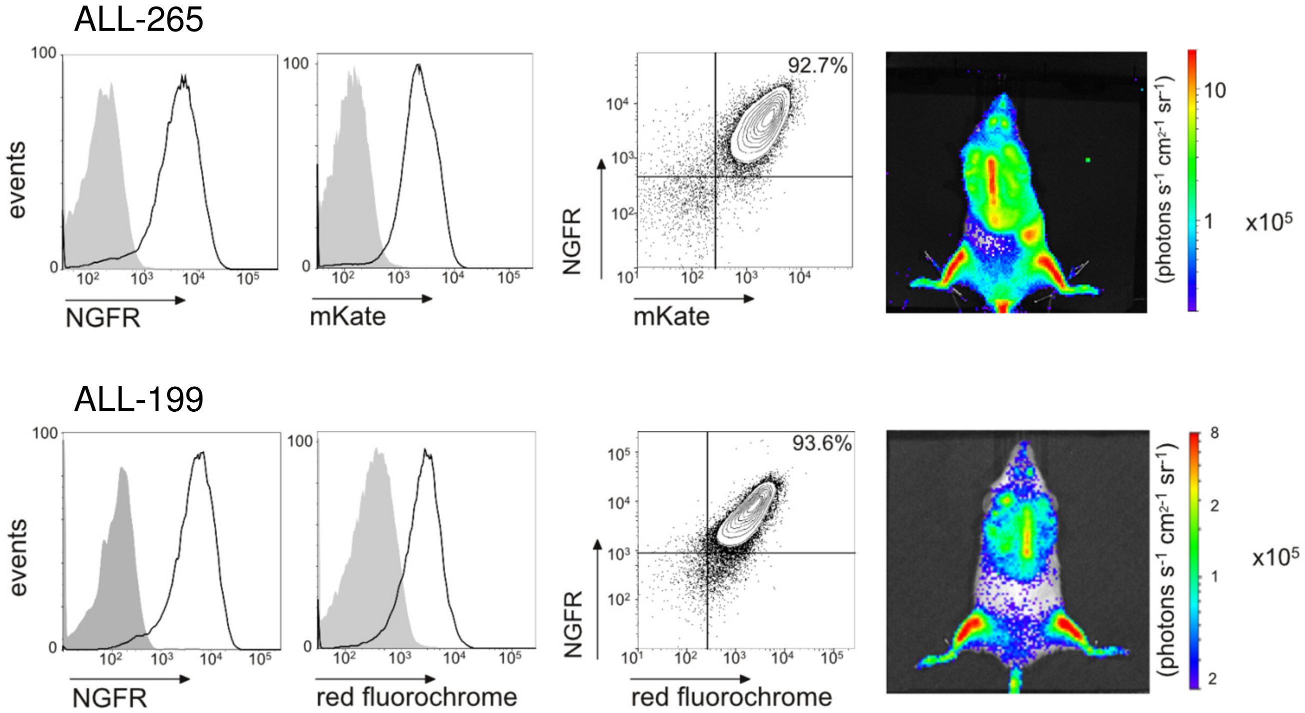

C

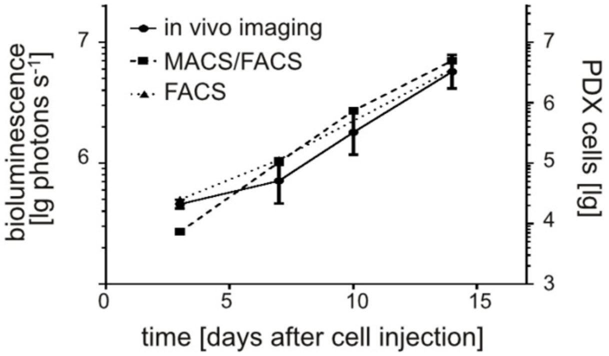

D

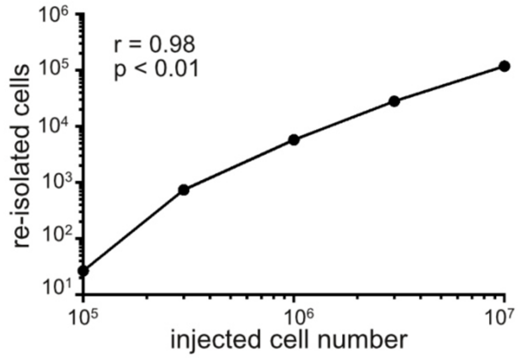

E

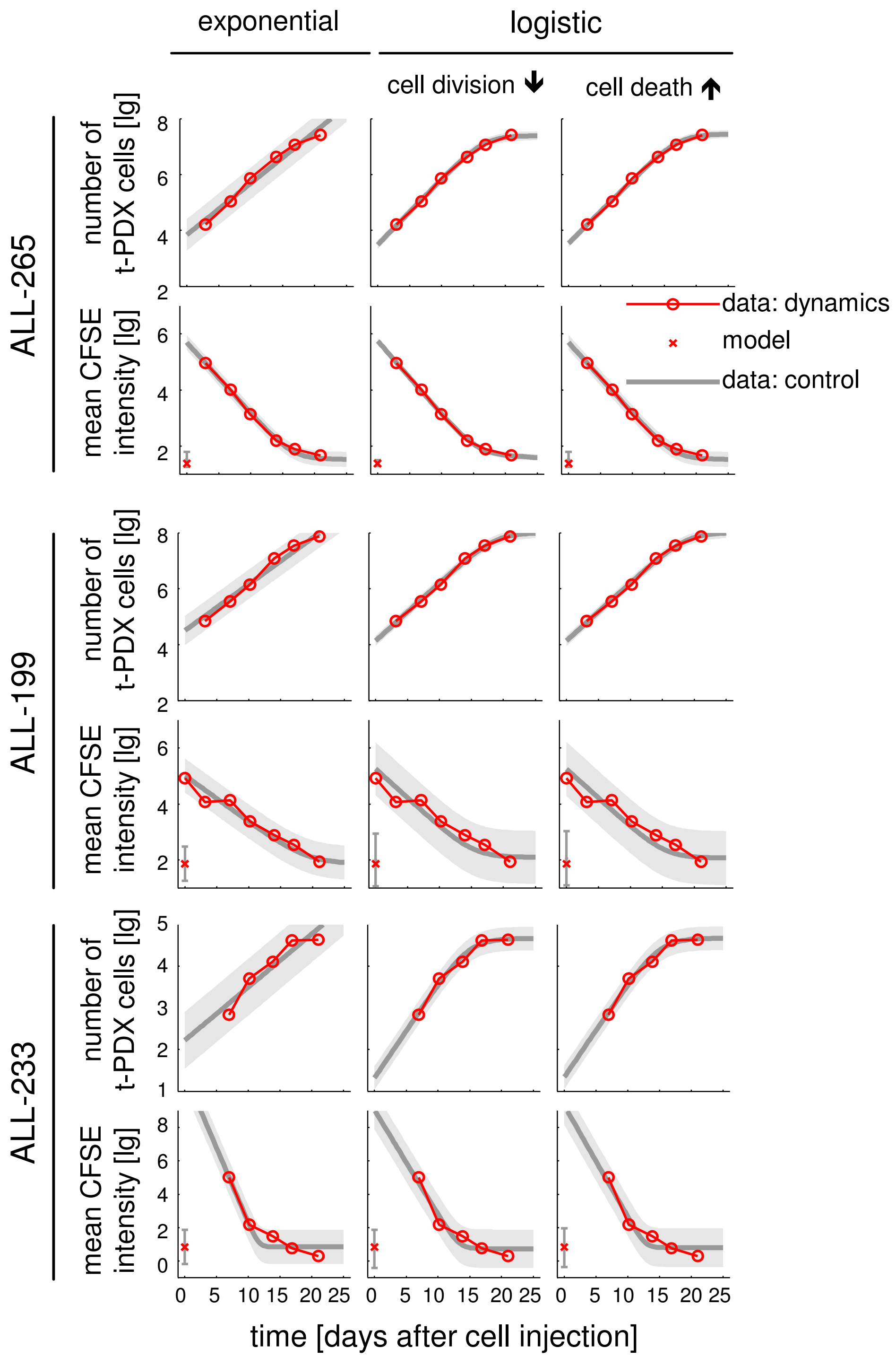

exponential
logistic
cell division
cell death
ALL-265
ALL-199
ALL-233
number of t-PDX cells [lg]
mean CFSE intensity [lg]
data: dynamics
model
data: control
time [days after cell injection]

F

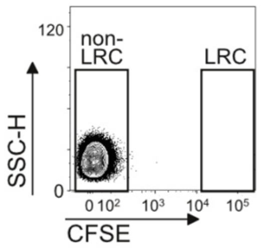

non-LRC
LRC
SSC-H
CFSE

G

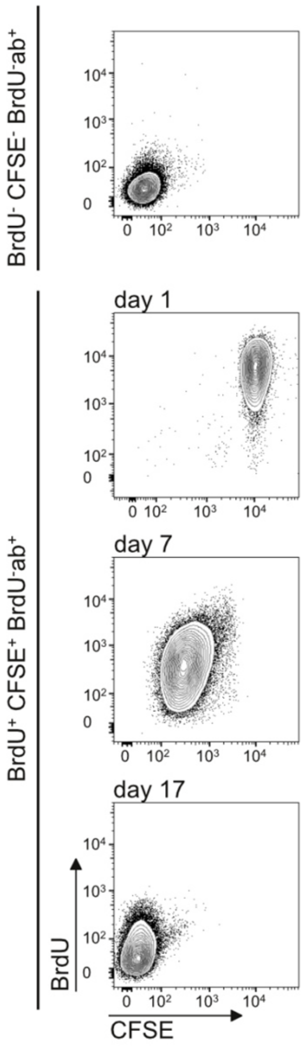

BrdU⁻ CFSE⁻ BrdU-ab⁺
BrdU⁺ CFSE⁺ BrdU-ab⁺
day 1
day 7
day 17
BrdU
CFSE

H

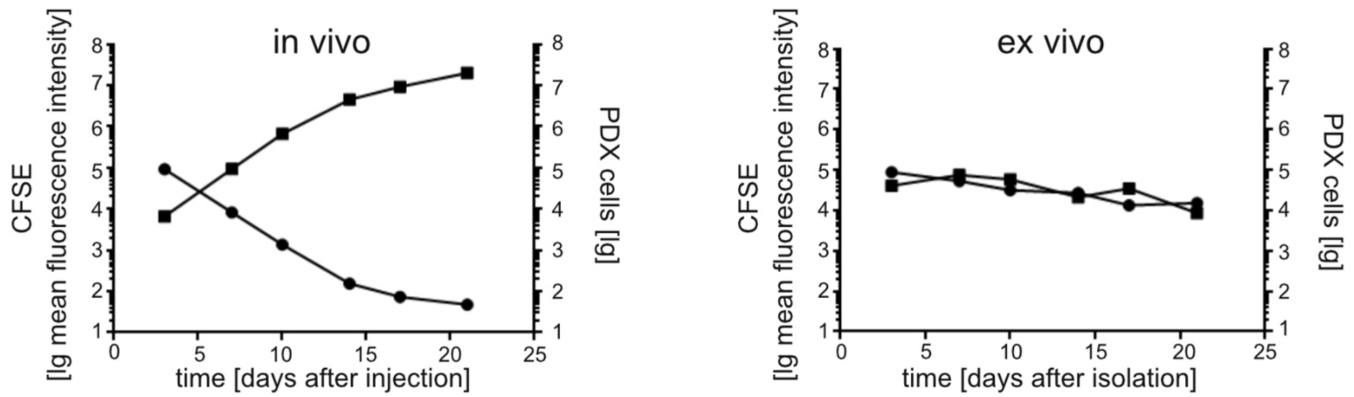

in vivo
ex vivo
CFSE [lg mean fluorescence intensity]
PDX cells [lg]
time [days after injection]
time [days after isolation]

Figure S1, related to Figure 1.
CFSE staining allows reliable monitoring of PDX ALL growth in mice.

(A) Lentiviral construct for equimolar expression of 3 transgenes; arrow indicates start of transcription; EF1α = elongation factor 1-alpha promoter; mKate = red fluorescent protein cloned from sea anemone Entacmaea quadricolor; NGFR = human low affinity nerve growth factor receptor lacking the intracellular signaling domain.
(B) Quality controls on enriched transgenic PDX ALL-265 or ALL-199 cells by flow cytometry or bioluminescence in vivo imaging.
(C) 10^7 ALL-265 cells were injected into groups of mice and one mouse was sacrificed at each time point. In vivo imaging was performed directly before cell harvesting and quantifying PDX cells by flow cytometry with and without prior MACS selection; mean of each group +/- standard error.
(D) Different cell numbers of ALL-199 cells were injected in mice at and re-isolated after 3 days; each dot indicates data from one animal.
(E) The measured numbers of PDX cells and the measured mean fluorescence intensities of CFSE were fitted with three mechanistic ordinary differential equation models assuming: exponential growth; logistic growth caused by a decreased rate of cell division at higher cell densities; and logistic growth caused by a increased rate of cell death at higher cell densities. The measured data (red circles and crosses), the best fit (gray line) and the noise related uncertainty intervals (gray shaded area) are depicted.
(F) No cells devoid of CFSE labeling are found in the LRC gate; flow cytometry analysis at day 0 of unlabeled ALL-265 PDX cells.
(G) Controls for BrdU and CFSE stainings; BrdU indicates feeding of mice and cells with BrdU; BrdU-ab indicates that cells were stained with the anti-BrdU antibody; “+” and “-” indicate that the procedures were performed or not, respectively.
(H) To compere behavior of PDX cells in vivo and ex vivo, 10^7 ALL-265 cells were injected into groups of mice and one mouse was sacrificed at each time point to isolate PDX cells (left panel); 10^7 fresh CFSE labeled ALL-265 PDX cells per ml were cultured on MS-5 feeder cells ex vivo (right panel).

Table S1, related to Figure 1.
Clinical data of patients donating diagnostic ALL cells for xenotransplantation and sample characteristics.

sample	type of ALL	disease stage*	age* [years]	sex	cytogenetics	passaging time§ [days]
**ALL-199**	BCP-ALL pediatric	2nd relapse	8	F	somatic trisomy 21; leukemic homozygous 9p deletion	42
**ALL-233**	BCP-ALL pediatric	initial diagnosis	<1	M	t(2;15)(p13;q15)	76
**ALL-265**	BCP-ALL pediatric	1st relapse	5	F	hyperploidy with additional 6, 13, 14, 17, 18, 21, X chromosome	43
**ALL-435**	BCP-ALL pediatric	initial diagnosis	<1	M	MLL-ENL, t(11;19)	40
**ALL-50**	BCP-ALL pediatric	initial diagnosis	7	F	BCR/ABL positive	45
**ALL-177**	BCP-ALL pediatric	initial diagnosis	8	F	TEL/AML1 positive	130
**ALL-230**	T-ALL pediatric	initial diagnosis	4	M	t(11;14)(p32;q11); rearrangement of TAL1-gene with the T-cell receptor locus	35
**ALL-256**	BCP-ALL adult	initial diagnosis	41	F	trisomy 8; BCR/ABL positive t(9;22)(q34;q11)	75
**ALL-363**	BCP-ALL adult	initial diagnosis	65	M	BCR-ABL positive t(9;22)(q34,q11)	60

BCP=B-cell precursor; *when the primary ALL sample was obtained; §time of passaging through mice refers to the time from injection of the sample until mice had to be sacrificed due to end stage leukemia

Table S2, related to Figure 1.
Two step procedure allows enrichment of minute numbers of PDX cells from mouse bone marrow.

mixed*		recovered		
**mouse bone marrow cells**	**PDX cells**	**number of cells**	**% recovery**	**enrichment Factor§**
$1x10^8$	1,250	1,234	99	81,000
$1x10^8$	12,500	10,262	82	9,700
$1x10^8$	37,500	34,679	92	2,666

*$1x10^8$ mouse bone marrow cells were mixed with different numbers of ALL-265 PDX cells expressing NGFR and mKate; MACS-based enrichment targeting NGFR-expressing cells was followed by flow cytometry-based enrichment targeting mKate-expressing cells; §enrichment factor was calculated as ratio from “number of mouse bone marrow cells” and “recovered number of cells”

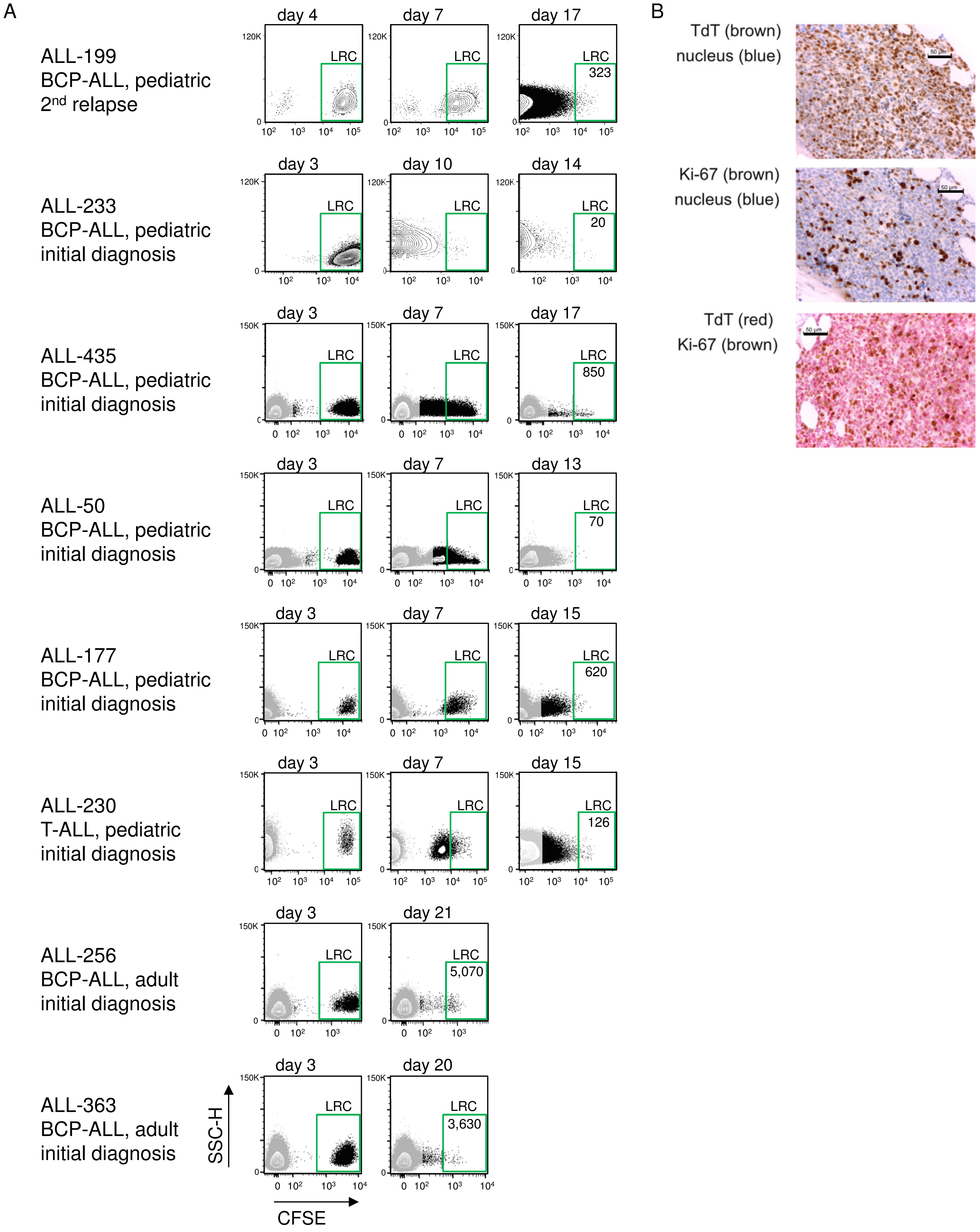
A
ALL-199
BCP-ALL, pediatric
2nd relapse
ALL-233
BCP-ALL, pediatric
initial diagnosis
ALL-435
BCP-ALL, pediatric
initial diagnosis
ALL-50
BCP-ALL, pediatric
initial diagnosis
ALL-177
BCP-ALL, pediatric
initial diagnosis
ALL-230
T-ALL, pediatric
initial diagnosis
ALL-256
BCP-ALL, adult
initial diagnosis
ALL-363
BCP-ALL, adult
initial diagnosis
day 4
day 7
day 17
day 3
day 10
day 14
day 13
day 15
day 21
day 20
LRC
323
20
850
70
620
126
5,070
3,630
SSC-H
CFSE
B
TdT (brown)
nucleus (blue)
Ki-67 (brown)
nucleus (blue)
TdT (red)
Ki-67 (brown)

Figure S2, related to Figure 2.

A rare, long-term dormant subpopulation exists in ALL PDX cells growing in mice.

(A) 10^7 CFSE labeled ALL-199 cells/mouse were injected into 3 mice and PDX cells were enriched from bone marrow of 1 mouse at each time point using MACS sorting targeting NGFR and FACS sorting targeting mKate; LRC and non-LRC were quantified by flow cytometry. One representative out of at least 10 independent experiments is shown. All further PDX samples did not express transgenes. Here, 10% of the entire bone marrow isolate was analyzed without a prior MACS enrichment step. Unstained cells represent mouse bone marrow cells and non-LRC. Day = number of days after injection of CFSE-labeled cells.

(B) Immunohistochemistry was performed using TdT to visualize all ALL blasts and Ki-67 to visualize proliferating cells in the diagnostic BM biopsy from one 69 years old female patient with BCR/ABL positive normal karyotype ALL; double staining (lowest panel) indicates frequent dormant ALL blasts as TdT positive, Ki-67 negative cells. Hemalum staining was used for nuclei; scale bar represents 50 µm.

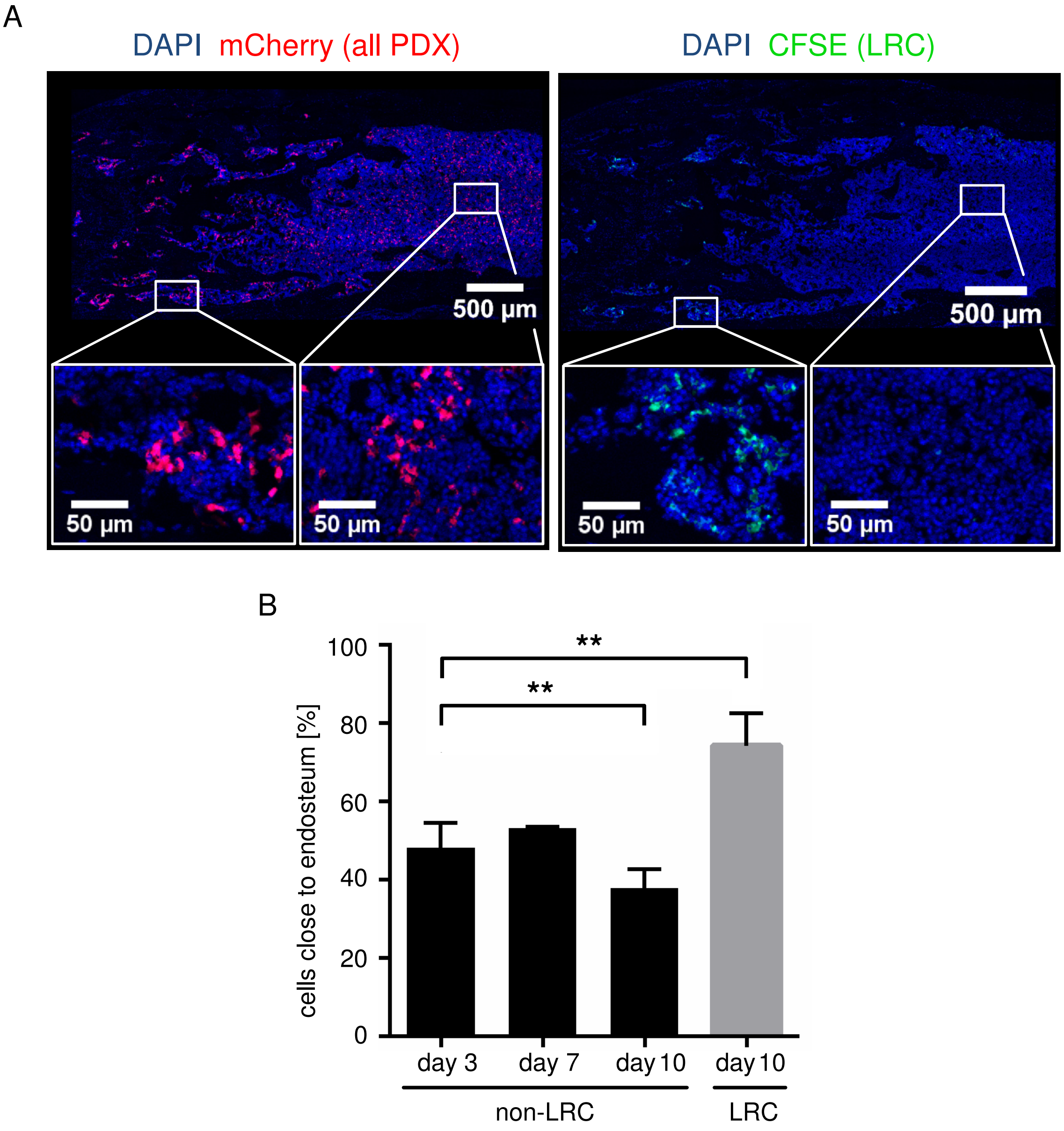

Figure S3, related to Figure 3.
LRC localize to the endosteum in ALL-199.

(A) Immunohistochemistry of consecutive murine bone marrow femur sections 10 days after injection of CFSE-stained PDX ALL-199 cells; mCherry (red) indicates all PDX cells, CFSE (green) indicates LRC.
(B) Kinetic for ALL-199; mean +/- standard error; * $p<0.05$; ** $p<0.01$ by two-tailed unpaired t-test.

Table S3, related to Figure 3.
LRC and non-LRC harbor similar numbers of leukemia initiating cells (LIC).

sample	number of cells injected per mouse*		time [days after injection] 20	28	41	48	62	75
ALL-265	LRC	333	0/2	0/2	0/2	0/2	2/2	2/2
		100	0/5	0/5	0/5	0/5	2/5	3/5
		10						8/19
	LIC frequency		1/40 (CI = 95%; lower = 1/84, upper = 1/19)					
	non-LRC	3333	0/3	1/3	3/3	3/3	3/3	3/3
		1000	0/5	0/5	2/5	4/5	5/5	5/5
		333	0/5	0/5	0/5	0/5	4/5	4/5
		100	0/5	0/5	0/5	0/5	2/5	2/5
		10						8/20
	LIC frequency		1/85 (CI = 95%; lower = 1/179, upper = 1/40)					
	number of cells injected per mouse		time [days after injection] 35	42	49	56	69	77
ALL-199	LRC	333	0/3	0/3	0/3	0/3	2/3	3/3
		100	n.d.	0/4	0/4	0/4	1/4	3/4
	LIC frequency		1/69 (CI = 95%; lower = 1/209, upper = 1/23)					
	non-LRC	1000	1/5	3/5	5/5	5/5	5/5	
		333	0/3	2/3	2/3	3/3	3/3	3/3
		100	0/4	1/4	1/4	3/4	4/4	4/4
	LIC frequency		1/100 or higher					

*LRC and non-LRC obtained 14 days after injection of CFSE labeled ALL-265 or ALL-199 cells were transplanted into secondary recipient mice in limiting dilutions at numbers indicated; bioluminescence in vivo imaging was performed repetitively at the indicated time points to determine engraftment; LIC frequency was calculated using the ELDA software; CI = confidence interval

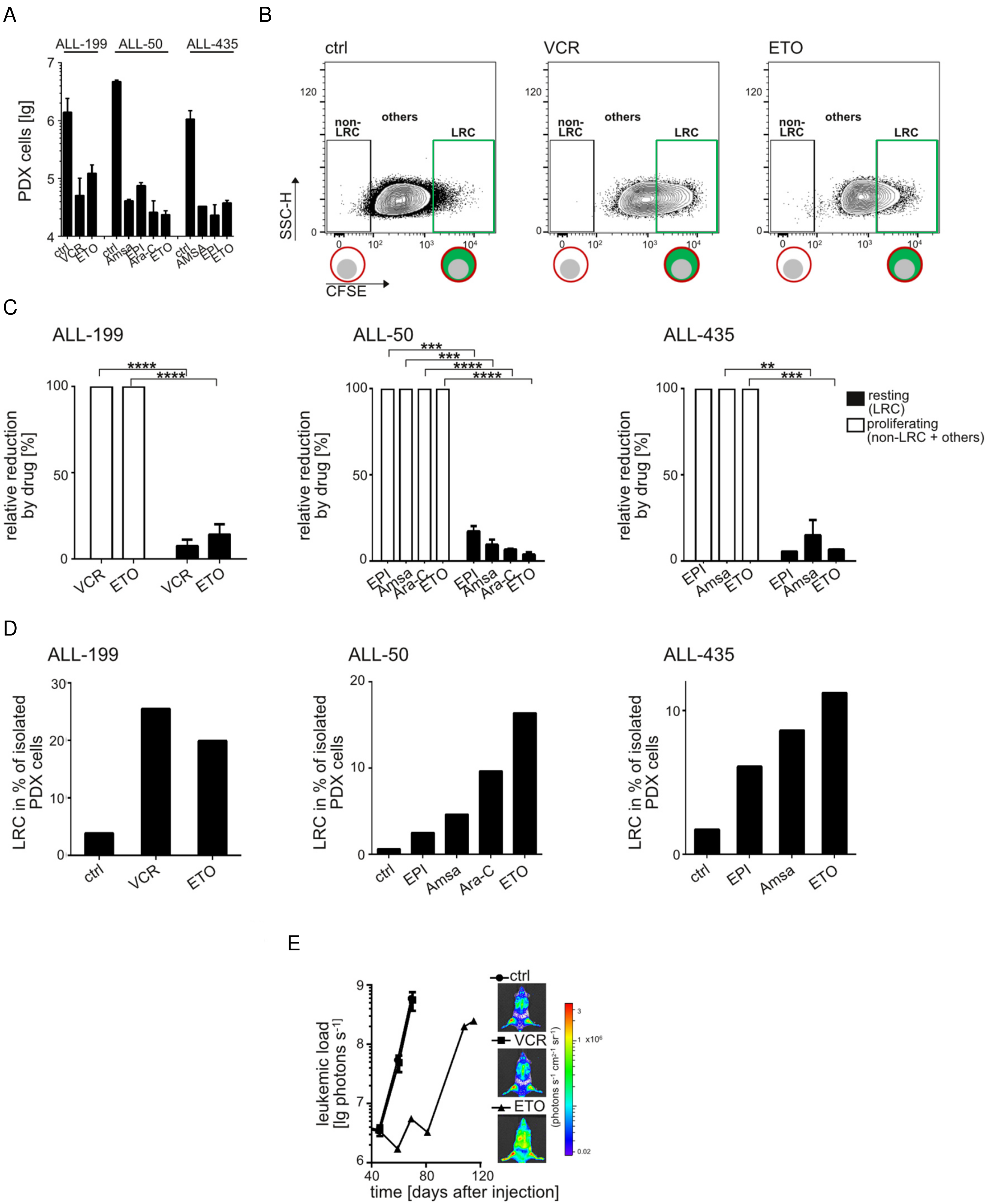

A
ALL-199
ALL-50
ALL-435
PDX cells [lg]
7
6
5
4
ctrl
VCR
ETO
ctrl
Amsa
EPI
Ara-C
ETO
ctrl
AMSA
EPI
ETO
B
ctrl
VCR
ETO
120
non-LRC
others
LRC
SSC-H
0
10^2
10^3
10^4
CFSE
C
ALL-199
ALL-50
ALL-435
relative reduction by drug [%]
100
50
0
****
***
**
resting (LRC)
proliferating (non-LRC + others)
VCR
ETO
EPI
Amsa
Ara-C
D
LRC in % of isolated PDX cells
30
20
10
ctrl
E
ctrl
VCR
ETO
leukemic load [lg photons s-1]
9
8
7
6
40
80
120
time [days after injection]
(photons s-1 cm-2 sr-1)
3
1 x10⁶
0.02

Figure S4, related to Figure 4.
LRC survive systemic drug treatment in vivo.

Mice were injected with 10^7 CFSE-labeled ALL PDX cells/mouse, were treated on day 7 and sacrificed on day 10; LRC and non-LRC were analyzed and re-transplanted into 1-2 secondary recipient mice at 2,000-5,000 LRC per mouse.

(A) Numbers of PDX cells isolated from mice with and without prior systemic drug treatment; mean of each group (n=8-11) +/- standard error.
(B) For ALL-199, a second relapse, 11 mice were treated with a single application of vincristine (VCR, 1.5 mg/kg i.v.), 8 mice were treated with a single application of etoposide (ETO, 75 mg/kg i.p.) and 8 control mice received buffer; shown are original data of representative mice.
(C) Quantification in all mice per group depicted as mean of relative drug effects on LRC compared to non-LRC (100%) +/- standard error. For ALL-50, a sample obtained at initial diagnosis, drugs were applied daily over 3 days and 2 mice were treated with cytarabine (AraC, 150 mg/kg i.p.), 2 mice with ETO (33 mg/kg i.p.), 2 mice with amsacrine (Amsa, 25 mg/kg i.p.) and 2 mice with epirubicine (EPI, 25 mg/kg i.p., single application). For ALL-435, another sample obtained at initial diagnosis, drugs were applied daily over 3 days and 2 mice were treated with ETO (33 mg/kg, i.p.), 2 mice with Amsa (25 mg/kg i.p.) and one mouse with EPI (25 mg/kg i.p., single application). ** $p< 0.01$, *** $p<0.001$, **** $p<0.0001$ by two-tailed unpaired t-test.
(D) Mean relative proportion of LRC in total PDX cells with and without treatment.
(E) To study their stem cell potential, LRC of ALL-199 LRC were isolated after treatment, re-transplanted and growth monitored by in vivo imaging mean of each group (n=1-2) +/- standard error. Imaging pictures from dpi 60 (ctrl, VCR) and dpi 108 (ETO).

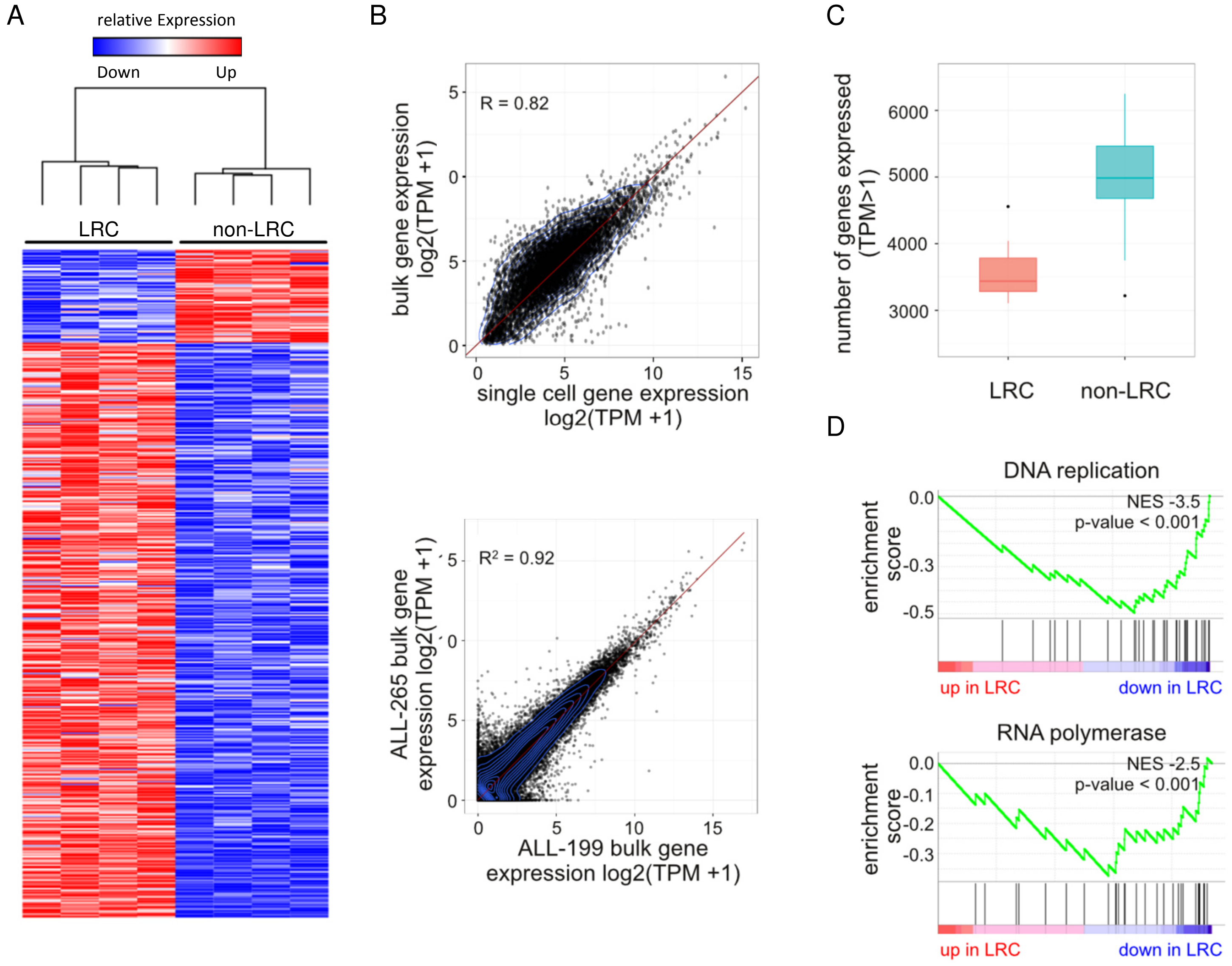

Figure S5, related to Figure 5.
Expression profile of LRC shows distinct changes to non-LRC.

15 days after transplantation of CFSE labeled PDX cells, LRC and non-LRC were subjected to RNA sequencing. For ALL-265, high quality single cell mRNA seq profiles were obtained from 15 LRC and 35 non-LRC cells. To combine single-cell and bulk RNA-seq data, median count data of single-cell experiments were summarized as a single expression profile for each LRC and non-LRC.

(A) Hierarchical clustering and gene expression heatmap across the 500 most differentially expressed genes comparing LRC and non-LRC in ALL-199 ($p < 0.01$).
(B) Comparison of Transcript Per Million (TPM) expression values between bulk versus single-cell ALL-265 (upper) and ALL-265 versus ALL-199 (lower).
(C) Quantification of expressed genes per cell (TPM > 1) in LRC versus non-LRC according to single-cell RNA-seq of ALL-265; shown is the median with upper/lower quartile and maximum/minimum, outliers are shown as dots.
(D) Gene set enrichment analysis for indicated KEGG pathways and the genes differentially regulated in LRC versus non-LRC.

Table S4, related to Figure 5.
List of 500 most differentially expressed genes between LRC and non-LRC in single cell RNA sequencing of ALL-265

Provided as an Excel file.

Table S5, related to Figure 5. Integrated LRC signature.

Provided as an Excel file.

Table S6, related to Figure 5.
KEGG pathways enriched with LRC versus non-LRC differentially expressed genes in combined analysis

Provided as an Excel file.

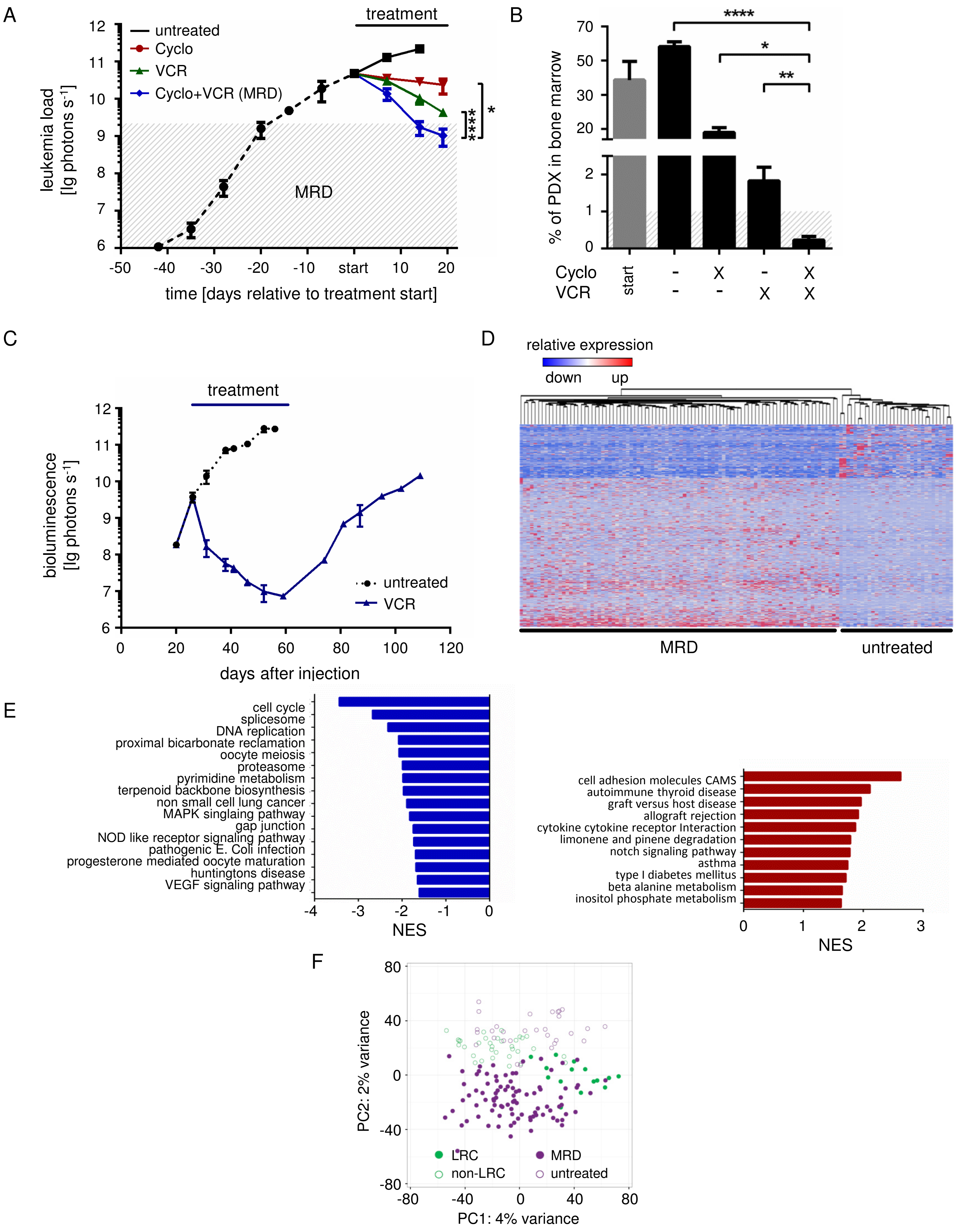
A
treatment
untreated
Cyclo
VCR
Cyclo+VCR (MRD)
leukemia load
[lg photons s-1]
MRD
time [days relative to treatment start]
****
*
B
% of PDX in bone marrow
Cyclo
VCR
start
C
treatment
bioluminescence
[lg photons s-1]
untreated
VCR
days after injection
D
relative expression
down
up
MRD
untreated
E
cell cycle
splicesome
DNA replication
proximal bicarbonate reclamation
oocyte meiosis
proteasome
pyrimidine metabolism
terpenoid backbone biosynthesis
non small cell lung cancer
MAPK singlaing pathway
gap junction
NOD like receptor signaling pathway
pathogenic E. Coli infection
progesterone mediated oocyte maturation
huntingtons disease
VEGF signaling pathway
NES
cell adhesion molecules CAMS
autoimmune thyroid disease
graft versus host disease
allograft rejection
cytokine cytokine receptor Interaction
limonene and pinene degradation
notch signaling pathway
asthma
type I diabetes mellitus
beta alanine metabolism
inositol phosphate metabolism
NES
F
PC2: 2% variance
PC1: 4% variance
LRC
non-LRC
MRD
untreated

Figure S6, related to Figure 6.
Characterization of cells at minimal residual disease.

(A-B) 10^7 ALL-265 cells were injected into 28 mice; when 40 % of bone marrow cells were human, therapy was started using vincristine (VCR, 0.25 mg/kg; n=4) or cyclophosphamide (Cyclo, 100 mg/kg; n=4) or a combination thereof (VCR+Cyclo; n=12), weekly for 3 weeks; VCR+Cyclo combination treatment had reduced tumor burden to minimal residual disease (MRD; < 1% human cells in bone marrow).

(A) Mean of each group +/- standard error; * $p<0.05$, **** $p<0.0001$ by two-tailed unpaired t-test; mice receiving buffer had to be sacrificed after two weeks of treatment due to end stage leukemia.

(B) Percentage of PDX ALL cells in mouse bone marrow as determined by flow cytometry post mortem as mean +/- standard error; * $p<0.05$, ** $p<0.01$, **** $p<0.0001$ by two-tailed unpaired t-test.

(C) To study their behavior after release of treatment pressure, ALL-199 cells were injected into 4 mice per group which were repetitively monitored by in vivo imaging; at substantial tumor burden, mice were treated with Vincristine (VCR) 0.4 mg/kg and left untreated thereafter; mean of each group +/- standard error.

(D-F) ALL-199 cells were injected into 19 mice; when 30 % of bone marrow cells were human, 5 untreated samples were harvested and one mouse were subjected to single cell sequencing; remaining mice received either buffer or vincristine (VCR, 0.25 mg/kg; n=5) or cyclophosphamide (Cyclo, 100 mg/kg; n=3) or a combination thereof (VCR+Cyclo; n=6) weekly for 2 weeks; when VCR+Cyclo combination treatment had reduced tumor burden to minimal residual disease (MRD; < 1% human cells in bone marrow), cells from the 6 VCR+Cyclo mice were isolated and one mouse were subjected to single cell sequencing.

(D) Hierachical clustering and gene expression heatmap across the 500 most differentially expressed genes between MRD cells and untreated cells in ALL-199 single cell RNA sequencing (MRD cells n=90; untreated cells n=32; $p < 0.01$; for gene annotation see Table S7).

(E) Significantly enriched KEGG pathways ($p < 0.05$) in MRD cells versus untreated cells as determined by geneset enrichment analysis.

(F) Principle component analysis of transcriptomes of 32 untreated control ALL-199 single cells and 90 MRD cells together with single cell data from LRC and non-LRC as in Figure 5C.

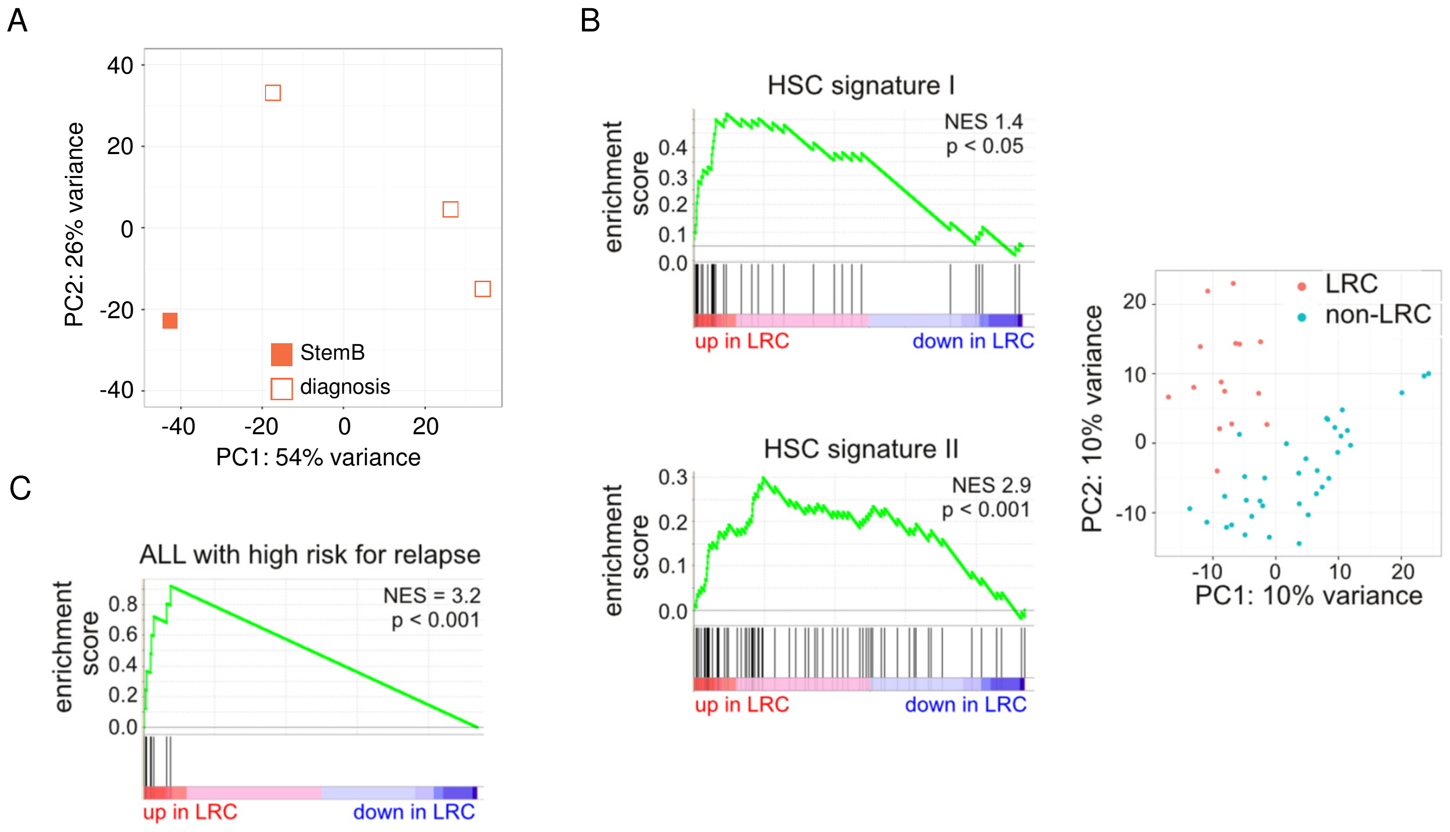

Figure S7, related to Figure 7.
LRC resemble primary MRD cells from patients.

(A) Principal component analysis of the bulk adult StemB sample compared to 3 bulk diagnosis samples of adult patients with BCR-ABL positive ALL.

(B) Gene set enrichment analysis of genes differentially expressed in LRC versus non-LRC and published signatures; HSC signature I = Georgantas et al., 2004; HSC signature II = Eppert et al., 2011 (left panel). Principal component analysis (PCA) of ALL-265 single cells on the basis HSC marker genes (Eppert et al., 2011) (right panel).

(C) Geneset enrichment analysis for a published gene signature prognostic for ALL with high risk of relapse (Kang et al., 2010).

Table S7, related to Figure 7.
Clinical data from BCP ALL patients of transcriptomes at diagnosis and/or MRD.

sample	age#	sex	multi-center study	genetic subtype	flow RG	proto-col RG&	stage after induc-tion II	day of MRD measure-ment §	BM blasts at MRD (%)$	sort
**1**	38	F	GMALL 0703	BCR-ABL	na	VHR	CR	71	0.24	StemB*
**2**	39	M	GMALL 0703	BCR-ABL	na	VHR	CR	71	0.32	StemB*
**1**	4	F	BFM 2009	ETV6/ RUNX1	MR	MR	na	na	na	$CD19^{+}$, $CD10^{+++}$, $CD20^{-}$
**2**	3	F	BFM 2009	ETV6/ RUNX1	MR	SR	na	na	na	$CD19^{+}$, $CD99^{bright}$, $CD10^{+++}$
**3**	5	M	BFM 2009	HD	MR	HR	na	33	0.69	$CD19^{+}$, $CD10^{+}$, $CD123^{+}$
**4**	18	M	BFM 2009	B OTHER	MR	HR	na	33	1.10	$CD19^{+}$, $CD10^{++}$, $CD45^{-/dim}$
**5**	3	F	BFM 2009	HD	MR	MR	na	33	0.13	$CD19^{+}$, $CD10^{++}$, $CD20^{dim}$

#age at diagnosis in years; F=female; M=male; GMALL=German Multicenter ALL Study Group; BFM=Berlin-Frankfurt-Münster; HD=high hyperdiploid karyotype; RG=risk group; na=not applicable; MR=medium risk; VHR=very high risk; SR=standard risk; HR=high risk; &therapy risk group (RG) assignment; §days after onset of treatment; BM=bone marrow; $in BCR-ABL positive samples, MRD was quantified by PCR using the BCR-ABL/ABL ratio; *StemB cells are $CD19^{+}$, $CD34^{+}$, $CD38^{-/low}$ according to Lutz et al., 2013; Hong et al., 2008; Castor et al., 2005

Table S8, related to Figure 7.
List of most significantly differentially expressed genes between primary samples from 5 primary ALL diagnosis and 3 MRD samples after 33 days of treatment.

Provided as an Excel file.

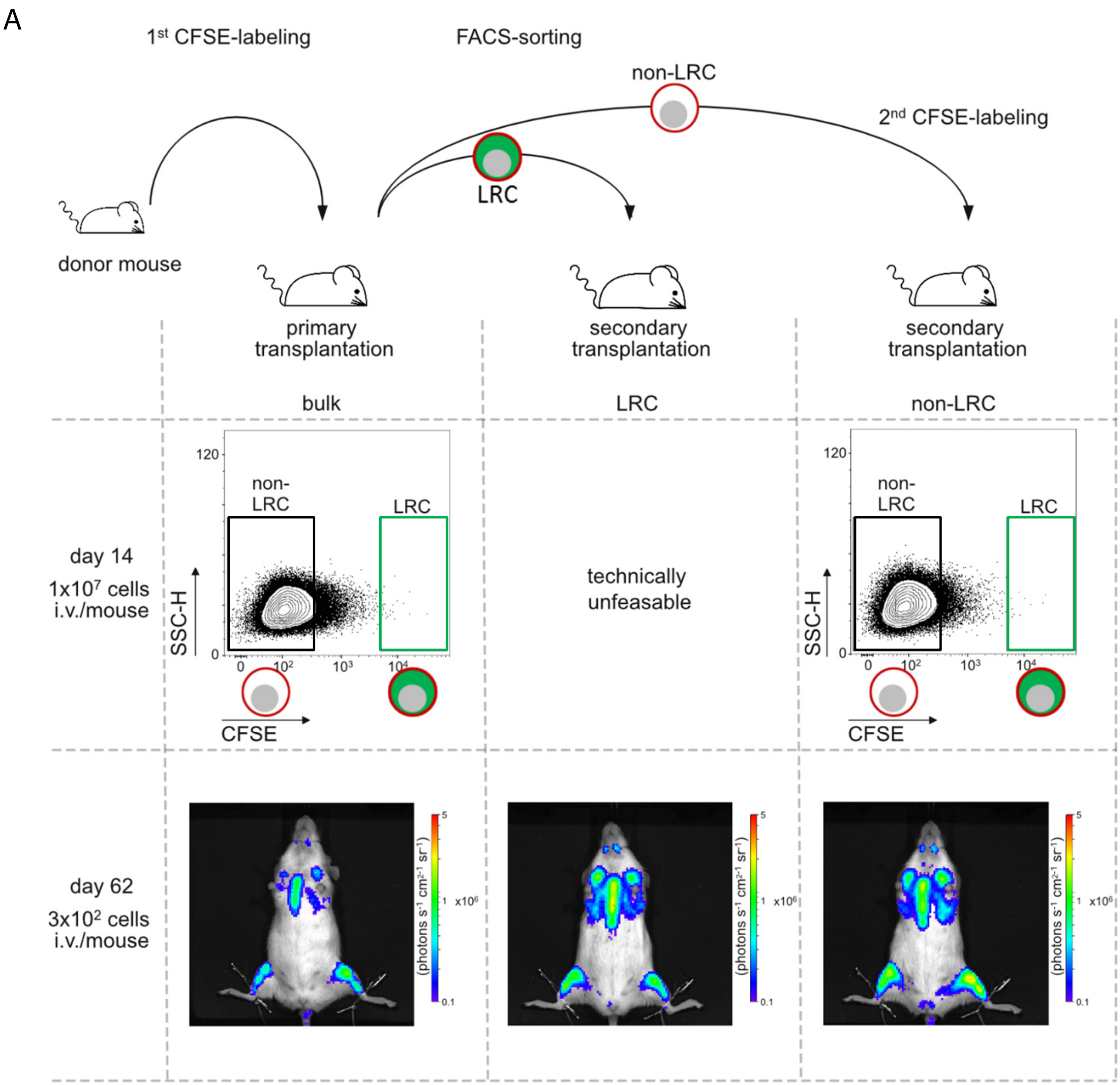
A
1st CFSE-labeling
FACS-sorting
non-LRC
2nd CFSE-labeling
LRC
donor mouse
primary transplantation
secondary transplantation
secondary transplantation
bulk
LRC
non-LRC
day 14
1x10^7 cells i.v./mouse
non-LRC
LRC
SSC-H
CFSE
technically unfeasable
day 62
3x10^2 cells i.v./mouse
(photons s^-1 cm^-2 sr^-1)
x10^6

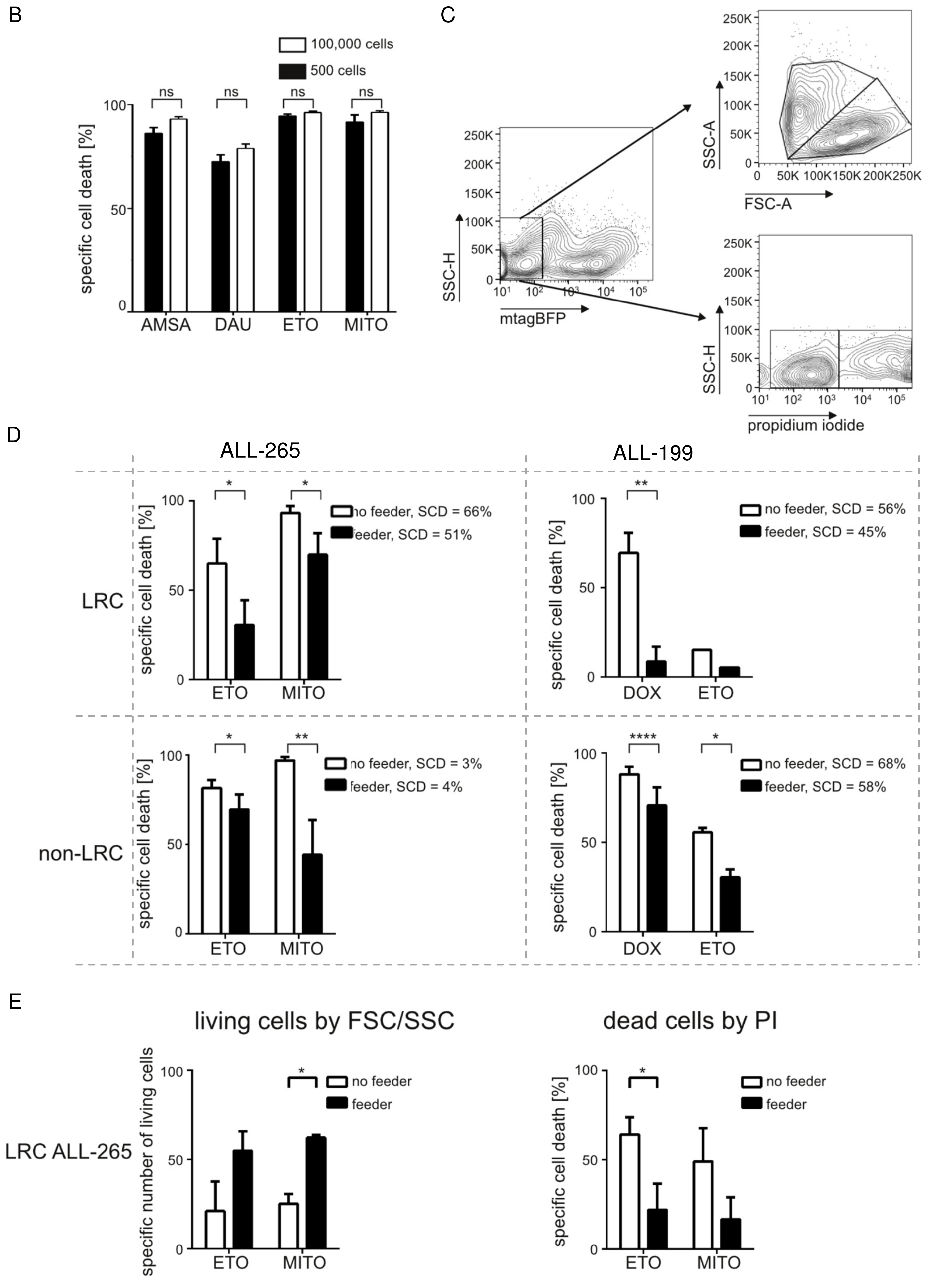

B
100,000 cells
500 cells
ns
specific cell death [%]
100
50
0
AMSA
DAU
ETO
MITO
C
250K
200K
150K
100K
50K
0
SSC-H
10^1 10^2 10^3 10^4 10^5
mtagBFP
SSC-A
0 50K 100K 150K 200K 250K
FSC-A
propidium iodide
D
ALL-265
ALL-199
LRC
non-LRC
no feeder, SCD = 66%
feeder, SCD = 51%
no feeder, SCD = 56%
feeder, SCD = 45%
no feeder, SCD = 3%
feeder, SCD = 4%
no feeder, SCD = 68%
feeder, SCD = 58%
*
**
****
DOX
E
living cells by FSC/SSC
dead cells by PI
LRC ALL-265
specific number of living cells
no feeder
feeder

F

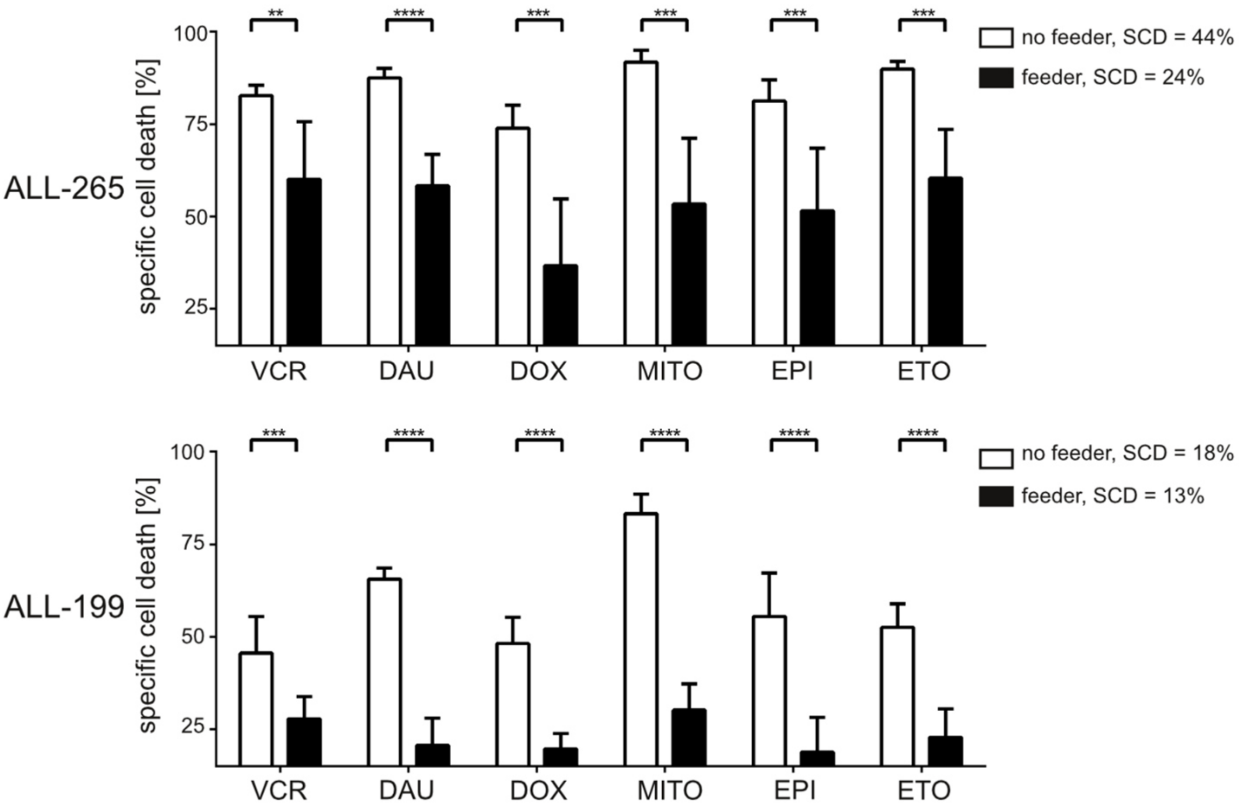
ALL-265
specific cell death [%]
100
75
50
25
**
****
***
***
***
***
VCR
DAU
DOX
MITO
EPI
ETO
no feeder, SCD = 44%
feeder, SCD = 24%
ALL-199
specific cell death [%]
100
75
50
25
***
****
****
****
****
****
VCR
DAU
DOX
MITO
EPI
ETO
no feeder, SCD = 18%
feeder, SCD = 13%

G

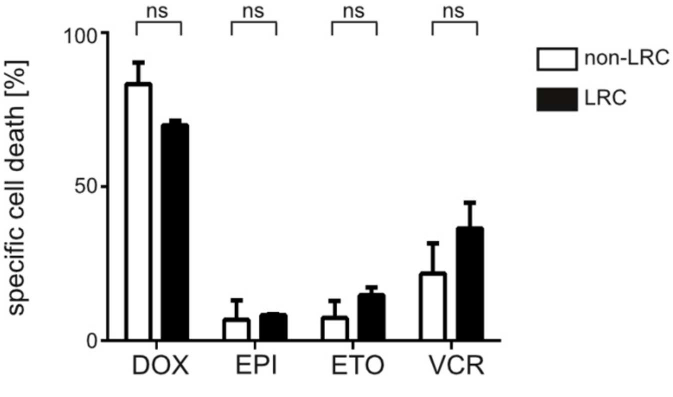
specific cell death [%]
100
50
0
ns
ns
ns
ns
DOX
EPI
ETO
VCR
non-LRC
LRC

Figure S8, related to Figure 7.
Identical growth behavior upon re-transplantation and identical ex vivo drug sensitivity in LRC versus non-LRC.

(A) Upper panel shows experimental procedure; ALL-265 cells were amplified in donor mice, CFSE labeled, re-transplanted into primary recipients and re-isolated after 14 days (left lane). Cells were separated into LRC (middle lane) and non-LRC (right lane) and re-transplanted into secondary recipients which were imaged after 62 days (lower row). non-LRC were additionally re-labeled with CFSE and re-transplanted at high numbers which was unfeasible for LRC due to their minor abundance (middle panel).
(B) 500 or 100,000 freshly isolated non-LRC (ALL-265) were stimulated ex vivo for 48 hours with the following cytotoxic drugs: amsacrine (AMSA; 18 µM), daunorubicine (DAU; 250 nM); etoposide (ETO; 30 µM) and mitoxantrone (MITO; 675 nM); shown is one experiment in triplicates +/- standard error; ns = not significant by two-tailed unpaired t-test. Specific cell death was determined by DAPI staining and specific cell death calculated thereof.
(C-F) Freshly isolated PDX cells were seeded in triplicates in the presence or absence of irradiated MS-5 cells expressing the blue fluorochrome mtagBFP. Cells were stimulated for 48-72 hours and all cells per well were removed by trypsin digestion and analyzed by flow cytometry. * $p<0.05$, ** $p<0.01$, *** $p<0.001$ and **** $p<0.0001$ by two-tailed unpaired t-test. SCD = spontaneous cell death in the absence of cytotoxic drugs.
(C) Feeder cells were excluded by gating on non-blue/mtagBFP-expressing cells; living cells were quantified in absolute and relative amounts using either forward/side scatter analysis or propidium iodide staining with similar results.
(D, E) 700-1,900 fresh LRC or non-LRC were stimulated with the following drugs: Etoposide (ETO; 3 µM) or mitoxantrone (MITO; 0.45 µM) for ALL-265 and etoposide (ETO; 15 µM) or doxorubicine (DOX; 0.5 µM) for ALL-199. E shows results obtained by forward sideward scatter analysis, F shows results obtained by propidium iodide (PI) staining as well as absolute number of surviving cells as estimated in forward/side scatter analysis. Shown are mean of up to 3 independent experiments; +/- standard error.
(F) 100,000 unsorted PDX ALL-265 were stimulated with vincristine (VCR; 0.3 µM), daunorubicine (DAU; 0.25 µM), doxorubicine (DOX; 0.5 µM), mitoxantrone (MITO; 0.45 µM), epirubicine (EPI; 0.4 µM) and etoposide (ETO; 3 µM) for 48 hours; 100,000 unsorted PDX ALL-199 were stimulated with vincristine (VCR; 0.03 µM), daunorubicine (DAU; 0.25 µM), doxorubicine (DOX; 0.5 µM), mitoxantrone (MITO; 0.25 µM), epirubicine (EPI; 0.4 µM) and etoposide (ETO; 3 µM) for 72 hours; mean of 9 data points from 3 independent experiments in triplicates is shown; +/- standard error; Welch's correction war required in two-tailed unpaired t-test for ALL-265 and DAU stimulation in ALL-199.
(G) 14 days after transplantation, LRC or non-LRC were isolated and 500-800 cells stimulated ex vivo for 48 hours with the following cytotoxic drugs: doxorubicine (DOX; 500 nM), epirubicine (EPI; 500 nM); etoposide (ETO; 30 µM) and vincristine (VCR; 300 nM). Specific cell death was determined after 48h by forward-side scatter and by DAPI staining and specific cell death calculated thereof; mean of 6 data points from 2 independent experiments in triplicates is shown +/- standard error; ns = not significant by two-tailed unpaired t-test.

Supplemental Experimental Procedures

The NSG mouse model of individual ALL

ALL blasts were obtained from children and adults treated within clinical multicenter studies. NSG mice (NOD/scid, IL2 receptor gamma chain knockout mice) were obtained from The Jackson Laboratory (Lund, Sweden). The animal model was performed as described (Liem et al., 2004). Briefly, fresh primary ALL cells were isolated by Ficoll gradient centrifugation from peripheral blood or bone marrow aspirates that had been obtained from leftovers of clinical routine sampling before onset of therapy. 10 million ALL cells were injected into 6-12 weeks old NSG mice via the tail vein. Engraftment was monitored by 2-weekly flow cytometry measurement of human cells in peripheral blood starting at week 6. ALL-265 was first engrafted by Jean Pierre Bourquin and Beat Bornhäuser in Zurich. Mice were sacrificed at first clinical signs of disease, as measured by quantification of human cells in peripheral blood or by in vivo imaging. From engrafted mice, PDX ALL cells were harvested from enlarged spleens and either directly re-injected or frozen at -190 °C and re-injected after thawing. Accuracy of sample identity was verified by repetitive finger printing using PCR of mitochondrial DNA (Hutter et al., 2004).

Cloning

The construct encoding for all 3 transgenes (Figure S1A) was generated by cloning a synthesized DNA-fragment (Eurofins Scientific, Luxembourg) encoding for mKate and a truncated form of the human nerve growth factor receptor lacking any intracellular signaling domain (NGFR; construct -mKateT2A-NGFR) into the pCDH-EF1α-extGLucT2A-copGFP Vector (Terziyska et al., 2012), leaving membrane anchored Gaussia luciferase and replacing copGFP gene using BamHI and SalI; T2A or P2A self-cleaving peptides enabled equimolar expression of the transgenes. For immunohistochemistry, cells were additionally transduced with a construct expressing mCherry which was obtained by amplifying mCherry from the pSicoR-U6-EF1a-mCherry Vector (addgene, Cambridge, MA, USA) and cloning it into the pCDH-EF1α-extGluc-T2A-copGFP Vector replacing the copGFP gene using BamHI and SalI.

Lentiviral transduction of ALL PDX cells and enrichment of transgenic cells

ALL-199 and ALL-265 were transduced using pCDH-EF1α-extGlucT2A-mKate-NGFR. Third generation packaging plasmids pMDLg/pRRE, pRSV-Rev and pMD2-G (Dull et al., 1998) were kindly provided by T. Schroeder. High-titer vesicular stomatitis virus (VSV) G protein-pseudotyped lentivector was prepared by transient 4-plasmid transfection of 293T cells using TurboFect Transfection Reagent (Thermo Scientific, Waltham, MA, USA) and supernatant concentration as described (Klier et al., 2008; Terziyska et al., 2012). The functional titer of virus was determined by transduction of NALM-6 B-ALL cell line cells with serial dilutions of the vector stock, followed by analysis of transgene positive cells using flow cytometry.

Generation of transgenic PDX cells was performed as previously described (Terziyska et al., 2012). In brief, PDX cells were transduced over night with lentivirus at MOI > 10 in the presence of 8 µg/ml polybrene. The next day, cells were washed thoroughly and injected into mice. After passaging, cells expressing the transgenes were enriched in two consecutive rounds by flow cytometry using FACSAriaIII (BD Biosciences) and gating on the red fluorochrome before cell re-amplification in mice. Although lentiviral transduction could in principle alter cells due to the transduction process or genomic integration, we could not detect adverse effects so far in comprehensive quality controls (Terziyska et al., 2012).

Bioluminescence in vivo imaging

For bioluminescence in vivo imaging mice were anesthetized with isoflurane and D-Luciferin (BIOMOL GmbH, Hamburg, Germany) dissolved in sterile PBS was used as substrate. Immediately after intravenous tail vein injection of 150 mg/kg of native D-Luciferin per mouse, mice were imaged for 30 seconds or up to 2 minutes using a field of view of 12.5 cm with binning 8, f/stop 1 and open filter setting using the IVIS Lumina II Imaging System (Perkin Elmer, MA, USA). The Living Image software 4.x (Perkin Elmer, MA, USA) was used for data acquisition and quantification of light emission using a scale with a minimum of 1.8×10^4 photons per second per cm^2 per solid angle of 1 steradian (sr) (Terziyska et al., 2012). Mice were considered positive for engraftment, if light emission by the entire mouse exceeded $5x10^5$ photons s^{-1} and positive signals were detected at typical sites at the lower extremities.

Reagents

For flow cytometry, analysis of NGFR, mKate, mCherry, BrdU, Annexin V, DAPI and PI was performed by flow cytometry, using BD LSRFortessa and BD FACSAriaIII (BD Biosciences, Heidelberg, Germany). The following antibodies were used: NGFR-PerCP-Cy5.5 (Biolegend, CA, USA), BrdU-APC, Annexin V-FITC detection kit (both

from BD Biosciences, Heidelberg, Germany). Mouse CD45-APC-Cy7 (Biolegend, San Diego, CA, USA) was used to exclude mouse cells.
BrdU incorporation was detected using the BrdU Flow Kit (BD Biosciences, Heidelberg, Germany). For analysis of cell viability, DAPI and/or PI were added to the cells at a concentration of 1µg/ml. All antibodies and reagents were used according to the manufacturer's instructions.
For chemotherapy treatments in vivo and ex vivo vincristine (VCR; Merck, Darmstadt, Germany), etoposide (ETO; Sigma Aldrich, St. Louis, USA), cyclophosphamide (Cyclo; Baxter, USA), epirubicine (EPI; Sigma Aldrich, St. Louis, USA), amsacrine (Amsa, Sigma Aldrich, St. Louis, USA), cytarabine (Ara-C; cell pharm GmbH, Bad Vilbel, Germany), daunorubicin (DAU; Sigma Aldrich, St. Louis, USA), mitoxantrone (MITO; Sigma Aldrich, St. Louis, USA) or doxorubicin (DOX, Sigma Aldrich, St. Louis, USA) were used.

Labeling of PDX cells with BrdU and CFSE
To label PDX cells with BrdU, donor mice were fed with BrdU (VWR, Radnor, PA, USA) during the 7 last days before cell isolation, at approximately 0.8 mg/kg/d BrdU using BrdU-containing drinking water. Freshly isolated PDX cells were labeled with CFDASE (Life Technologies, Carlsbad, CA, USA) according to manufacturer's instructions. Cells were washed and directly injected into recipient mice. The procedures resulted in both BrdU and CFSE positivity of well above 98% of PDX cells, as validated by flow cytometry. As PDX ALL cells are heterogeneous in size, loss of CFSE appears as continuum in flow cytometry devoid of the distinct peaks known from normal lymphocytes.

Enriching human PDX ALL cells from murine bone marrow
The aim was to isolate and enrich minute numbers of human PDX ALL cells out of a major excess of murine bone marrow cells. The procedure was designed according to published protocols for isolating normal mouse hematopoietic stem cells from murine bone marrow (Takizawa et al., 2011). Our studies concentrated on the first 3 weeks of ALL growth in mice, when low tumor burden is mainly restricted to bone marrow without major involvement of further organs (data not shown).
<u>Isolation of bone marrow cells from mice</u>
To collect as many bone marrow cells as possible from each mouse, the hip, femura, tibiae, spine and sternum were isolated and crushed in a porcelain mortar. The suspension was washed with cold PBS, filtered through a 70 µm cell strainer, washed again with PBS and re-suspended in cold PBS at $1x10^7$ cells/ml.

<u>Step 1: Enriching NGFR expressing PDX cells from the bone marrow suspension</u>
A first enrichment step consisted in magnetic cell separation (MACS) of NGFR-expressing PDX ALL cells from the entire mouse bone marrow isolated. 20 µl per $1x10^7$ cells of anti-human NGFR MicroBeads (Miltenyi Biotech, Bergisch Gladbach, Germany) were added to the isolated mouse bone marrow cell suspension and incubated 10 minutes at 4°C. A maximum of $2x10^8$ cells were loaded onto a LS column (Miltenyi Biotech, Bergisch Gladbach, Germany), prepared according to manufacturer's instructions. Cells were recovered from the column according to manufacturer's instructions and washed with PBS.

<u>Step 2: Enriching and quantifying fluorochrome expressing PDX cells from NGFR-expressing cells</u>
The second consecutive enrichment step consisted in flow cytometry enrichment of red fluorochrome expressing cells out of the cell suspension obtained after MACS enrichment. Cells obtained after MACS enrichment were stained with DAPI to exclude dead cells and with anti-muCD45-APC-Cy7 (anti-mouse CD45) to exclude murine hematopoietic cells. Cells were quantified and sorted using a BD FACSAriaIII (BD Biosciences, Heidelberg, Germany), gating (i) on the lymphocyte gate in forward/side scatter, (ii) the negative gate for both mouse CD45 and DAPI and ultimately (iii) the positive gate for the red fluorochrome.
Alternatively and to quality control for the MACS enrichment step, 10% of the entire population of bone marrow cells was directly analyzed by flow cytometry without prior MACS enrichment and with the identical staining procedure (Figure S1D). The disadvantage of this procedure lies in the prolonged periods of time required for flow cytometric cell enrichment disabling measuring more than 10% of all cells.

Enriching dormant cells (LRC) from human PDX ALL cells
<u>Step 3: Separating PDX ALL cells into LRC and non-LRC</u>
Separating PDX ALL cells into LRC and non-LRC was performed within the flow cytometry enrichment step described above (Step 2) by addition of a 4th gating strategy. Additionally to gating on (i) the lymphocyte gate in

forward/side scatter, (ii) the negative gate for both mouse CD45 and DAPI and (iii) the positive gate for the red fluorochrome, gating (iv) on CFSE was used to discriminate LRC and non-LRC as shown in Figure 1D. To set gate 4, CFSE intensity was measured at day 3 after injection when major bleaching had stopped; maximum CFSE MFI was used to define start of any cell proliferation ("0 divisions"). Maximum CFSE MFI was divided by factor 2 to calculate CFSE bisections mimicking cell divisions. 7 CFSE MFI bisections or more were defined as entire loss of the CFSE signal characterizing non-LRC. The LRC gate was set to include all cells harboring high CFSE signal of below 3 bisections of the maximum CFSE MFI (Schillert et al., 2013) (Figure 1D). All further analyses were done and analyzed with the same instrument settings and gates as determined using the sample on day 3 sample of the experiment.

Ex vivo culture of PDX cells

PDX cells were cultured in RPMI medium supplemented with 20% FSC, 1% pen/strep, 1% gentamycin, 6 mg/l insulin, 3 mg/l transferrin, 4 µg/l selenium (ITS-G, Gibco, San Diego, CA, USA), 2 mM glutamine, 1 mM sodium pyruvate, 50 µM α-thioglycerol (Sigma-Aldrich, St. Louis, MO, USA).

Limiting dilution transplantation assay (LDTA)

For LDTAs, NSG mice were injected intravenously with different amounts of PDX cells from ALL-265 or ALL-199. Development of leukemia was monitored by bioluminescence in vivo imaging every 7 to 14 days after cell injection. LIC frequencies were determined according to Poisson statistics, using the ELDA software application (http://bioinf.wehi.edu.au/software/elda/) (Hu and Smyth, 2009).

Drug stimulation ex vivo

500 LRC and 500 or 100,000 non-LRC were cultured in 100 µl medium in 96-well plates, in cell concentrations of 5,000 cells/ml or 10^6 cells/ml. Cytotoxic drugs were added in triplicates at the clinically relevant concentrations described in each Figure legend. Cell death was measured after 48h by forward-side scatter and DAPI or propidium iodide staining in a flow cytometer. Specific cell death induced by each drug was calculated as follows: specific cell death = [(cell death(stimulated) – cell death(control) / (100 – cell death(control))] * 100.

For co-cultures, MS-5 cells stably expressing mtagBFP as blue fluorochrome were irradiated in suspension with 60 Gy and seeded at 10^4 per well in a 96 well plate; 700 - 1,900 freshly isolated PDX cells were incubated with and without feeder cells in 100 µl medium for 24-48h stimulated with conventional cytotoxic drugs at clinically relevant concentrations; entirely all cells of each well were removed using trypsin digestion and stained with propidium iodide; feeder cells were excluded by gating on non-blue-expressing cells independently from CFSE or propidium iodide staining; absolute numbers of living PDX cells were measured using forwardside scatter analysis and cell death was additionally measured by propidium iodide staining in flow cytometry.

In vivo treatment of mice

For treatment of LRC, NSG mice were injected i.v. with $1x10^7$ PDX cells. 7 days after cell injection, control animals received physiological salt solution i.p., while treatment group mice were injected with chemotherapeutic drugs as indicated in Figure legends. Mice were taken down 3 days later, bone marrow was collected, and PDX cells were isolated and analyzed for CFSE label retention. For calculation of relative drug effect on LRC compared to non-LRC (Figure 4D), first absolute number of control LRC or non-LRC were divided by the absolute number of treated LRC or non-LRC, respectively. In a second step, relative cell reduction in non-LRC was set to 100% and cell reduction in LRC was calculated relative to non-LRC. A maximum of 4 animals could be included into the same experiment as a maximum of 4 animals could be analyzed for CFSE distribution at the same day.

To obtain cells at minimal residual disease, $1x10^6$ ALL-199 or ALL-265 were injected into 19 NSG mice and leukemic growth was followed by weekly in vivo imaging. Treatment was started at an average of $1x10^{11}$ photons s^{-1}, when untreated cells were recovered from 5 mice. Mice were divided into different treatment groups which were treated as indicated in Figures legends.

Immunostaining of bone marrow sections

Mouse femurs were fixed in zinc formalin fixative for 1 day at 4°C. Bones were washed with PBS and decalcified with Osteosoft (Merck) for 3 days at 4°C, infiltrated with 30% sucrose for 1 day at 4°C, embedded in O.C.T. compound (Sakura) and stored at -80°C. Cryosections of decalcified bones were obtained by using the CryoJane tape transfer system (Leica). For immunostaining, sections were permeabilized and blocked with 5% goat serum and 0.1% Tween-20 serum in PBS for 45 min at room temperature. Primary antibodies were applied for 1 day at 4°C and

followed by secondary antibody incubation for 45 min at room temperature. Sections were finally stained with 10 mg/ml DAPI for 15 min and the slides were mounted with prolong gold (Invitrogen). Washing in between each staining steps was performed. Primary antibodies were rabbit-anti-FITC (ThermoFisher; 1:100) and rabbit-anti-mCherry (Abcam; 1:100) and goat-anti-rabbit with Alexa 594 (Invitrogen) was used as secondary antibody. Images were acquired on a Leica SP5 confocal microscope and analyzed with ImageJ. CFSE signal intensity was adapted to the mCherry signal by adjusting the 8 bit threshold for quantification of the LRC population based on FACS data. The endosteal region was defined as less than 100 µm from bone matrix (Nombela-Arrieta et al., 2013). Cells of interests were counted semi-automatically by the program ImageJ. Relative endosteal cells were calculated as absolute cell numbers in the endosteal region divided by absolute cell numbers in entire bone marrow section. Mean and standard error were calculated from at least 3 sections of each femur from 2 independent mice.
For immunhistology of primary bone marrow biopsies, bone marrow biopsies were fixed and stained using the avidin-biotin-peroxidase complex (ABC) method (Hsu et al., 1981) and anti-TdT antibody (Leica, Germany) and anti-Ki-67 antibody (Dako, Germany).

Flow cytometric cell enrichment of StemB cells from BCR-ABL positive ALL
Thawed mononuclear bone marrow cells were handled on ice and stained with CD3-FITC, CD19-PE, CD34-APC, CD38-PECy7 (all Becton Dickinson) and DAPI 0.1 µg/ml; StemB cells expressing $CD3^-$ $CD34^+$ $CD38^{-/low}$ $CD19^+$ cells were enriched using the FACSAria™ (Becton Dickinson) according to (Castor et al., 2005; Hong et al., 2008; Lutz et al., 2013).

Flow cytometric cell enrichment of diagnostic and MRD pediatric BCP-ALL cells
Thawed mononuclear bone marrow cells were handled on ice and stained using antibodies appropriate for minimal residual disease (MRD) detection against CD10, CD19, CD20, CD34, CD38, CD45, CD58, CD99, and CD123. Leukemic blasts were enriched to >95% purity using a FACSAria™ Fusion cell sorter equipped with an automatic cell deposition unit (ACDU; Becton Dickinson); data analysis was performed using the FACSDiva™ software (Becton Dickinson).

Bulk RNA sequencing library construction
PDX LRC and non-LRC cell populations were sorted into lysis buffer composed of 0.2 % Triton X-100 (Sigma) and 2 U/µl of RNAse Inhibitor (Life Technologies). ERCC spike-in controls (Life Technologies) were added to the cell lysis mix at 1:5,000 dilution. RNA was cleaned-up from the crude lysate with Agencourt RNAclean XP SPRI beads (Beckman-Coulter). cDNA was synthesized and pre-amplified from 5 µl of lysate according to the Smart-seq2 protocol (Picelli et al., 2013).
For each pediatric ALL MRD and PDX MRD sample, 2000 cells were sorted into TCL buffer (Qiagen). RNA was cleaned up using Agencourt RNAclean XP SPRI beads from half of the lysate and used to generate UMI-seq libraries as previously described (Parekh et al., 2016).
For all libraries, 1 ng of pre-amplified cDNA was used as input for tagmentation by the Nextera XT Sample Preparation Kit (Illumina), where a second amplification round was performed for 12 cycles.

RNA sequencing library construction of primary StemB single cells
Single adult StemB cells were deposited in 96-well plates containing 5 µl lysis buffer composed of a 1:500 dilution of Phusion HF buffer (NEB). Single-cell RNA-seq libraries were constructed using the SCRB-seq method according to (Soumillon et al., 2014).

RNA-seq analysis
All sequencing reads were demultiplexed from the Nextera (i5 and i7) indices.
For Smart-seq libraries, demultiplexed reads were aligned to the human genome (hg19) and ERCC reference using NextGenMap (Sedlazeck et al., 2013). Count data was generated from mapped reads using featureCounts (Liao et al., 2014) on ENSEMBL gene models (GRCh38.74).
For UMI-seq and SCRB-seq libraries, read pairs were processed by tagging the cDNA read with barcode and UMI sequences using the Drop-seq tools pipeline (Macosko et al., 2015). Tagged reads were aligned to the human genome (hg19) using STAR (Dobin et al., 2013) and sample-wise count tables generated using Drop-seq tools.

To remove noise from lowly expressed genes, count data sets were subjected to data-driven gene filtering using the HTSFilter R package (Rau et al., 2013). For PDX single cell sequencing libraries, only those cell data sets were used

which came from viable cells, obtained at least 1 million reads and detected at least 3000 genes (TPM > 1). For combined bulk (1x ALL-265; 4x ALL-265) and single cell (1x ALL-265) analysis (Figure 5), filtered single cell datasets were included summarized by gene-wise median read count as one LRC and non-LRC replicate. Differential expression (DE) analysis was done in the DESeq2 R package (Love et al., 2014) using the Wald test.
A combined LRC signature (ALL-265 & ALL-199; 250 genes; FC > 1; padj < 0.05) was obtained from this data.
Overrepresentation of significantly differentially expressed genes in KEGG pathways was tested by a fixed network enrichment analysis (FNEA) implemented in the neaGUI R package (Alexeyenko et al., 2012).
We applied hierarchical clustering gene-wise and sample-wise with complete linkage based on Euclidian distances of variance stabilized counts of DE genes (500 genes with lowest padj, FDR adjustment (Benjamini and Hochberg, 1995)) and plotted as heatmap. The reference expression value is the expression average of non-LRC cells.
Principal Component Analysis (PCA) of LRC PDX cells was performed on variance stabilized counts of the 500 most variable genes to display the main variance of the samples.
To analyse combined data from all obtained single-cells, count data was normalized accounting for batch effects using SCONE (Risso et al., 2014). PCA and k-means clustering of combined single-cell data was performed on all shared detected genes.
Gene set enrichment analysis was performed using GSEA Desktop Application. For ranking all genes, a metric score was calculated by multiplying their log fold changes with the –log10(p_adj) values and submitted to the Pre-Ranked GSEA tool. The statistical significance was determined by 1000 gene set per mutations (Subramanian et al., 2005).

Dynamical modelling

The growth behavior of ALL cells in bone marrow has been analyzed using mechanistic ordinary differential equation models describing the population growth and the CFSE dilution. To gain insights into the in vivo growth behavior of ALL cells, we compared three alternative models. The first model assumed exponential growth, the second model assumed logistic growth caused by a decreased rate of cell division at higher cell densities, and the third model assumed logistic growth caused by an increased rate of cell death at higher cell densities.
The state variables of all three models are the cell number $n(t)$ and the mean fluorescence intensity $m(t)$. The governing equations for $n(t)$ and $m(t)$,

$$dn/dt = (\alpha(n)-\beta(n))\, n, \qquad N(0) = n_0,$$
$$dm/dt = -\,(\alpha(n)+k)\, m, \qquad m(0) = m_0,$$

have been deduced from existing partial differential equation models (Hasenauer et al., 2012). In this governing equations $\alpha(n)$ denotes the rate of cell division, $\beta(n)$ denotes the rate of cell death, and k denotes the rate of CFSE degradation.
The three model alternatives only differed in the parameterization of rates of cell division and cell death, $\alpha(n)$ and $\beta(n)$. For the exponential growth model all rates were constant, $\alpha(n) = \alpha_0$ and $\beta(n) = \beta_0$. For the logistic growth model with decreasing cell division at higher cell densities $\alpha(n) = \alpha_0\,(1-n/n_\alpha)$ and constant $\beta(n) = \beta_0$ were used. For the logistic growth model with increasing cell death at higher cell densities constant $\alpha(n) = \alpha_0$ and $\beta(n) = \beta_0\,(1+n/n_\beta)$ were used. As the measurement of the mean intensity induced by CFSE is corrupted by the cell's autofluorescence, we measure $m'(t) = m + m_a$, in which m_a denotes the average autofluorescence.
The parameters of the three models were determined from measurement of $n(t)$ and $m'(t)$ using maximum likelihood estimation, assuming normally distributed measurement noise. For model comparison the Akaike information criterion (AIC) was used.

Statistics

All statistical analyses were calculated using GraphPad Prism 6 software. Two-tailed unpaired t-test was applied to evaluate differences after drug treatment. F-test was applied to compare standard deviations; in cases, when standard deviations differed significantly, Welch's correction was applied. LIC frequencies were calculated according to Poisson statistics using the ELDA software application (http://bioinf.wehi.edu.au/software/elda) (Hu and Smyth, 2009).

Supplemental References

Alexeyenko, A., Lee, W., Pernemalm, M., Guegan, J., Dessen, P., Lazar, V., Lehtio, J., and Pawitan, Y. (2012). Network enrichment analysis: extension of gene-set enrichment analysis to gene networks. BMC Bioinformatics *13*, 226.

Benjamini, Y., and Hochberg, Y. (1995). Controlling the False Discovery Rate: A Practical and Powerful Approach to Multiple Testing. Journal of the Royal Statistical Society Series B, Methodological *57*, 289-300.

Castor, A., Nilsson, L., Astrand-Grundstrom, I., Buitenhuis, M., Ramirez, C., Anderson, K., Strombeck, B., Garwicz, S., Bekassy, A. N., Schmiegelow, K.*, et al.* (2005). Distinct patterns of hematopoietic stem cell involvement in acute lymphoblastic leukemia. Nat Med *11*, 630-637.

Dobin, A., Davis, C. A., Schlesinger, F., Drenkow, J., Zaleski, C., Jha, S., Batut, P., Chaisson, M., and Gingeras, T. R. (2013). STAR: ultrafast universal RNA-seq aligner. Bioinformatics *29*, 15-21.

Dull, T., Zufferey, R., Kelly, M., Mandel, R. J., Nguyen, M., Trono, D., and Naldini, L. (1998). A Third-Generation Lentivirus Vector with a Conditional Packaging System. Journal of Virology *72*, 8463-8471.

Hasenauer, J., Schittler, D., and Allgöwer, F. (2012). Analysis and Simulation of Division- and Label-Structured Population Models. Bull Math Biol *74*, 2692-2732.

Hsu, S. M., Raine, L., and Fanger, H. (1981). Use of avidin-biotin-peroxidase complex (ABC) in immunoperoxidase techniques: a comparison between ABC and unlabeled antibody (PAP) procedures. J Histochem Cytochem *29*, 577-580.

Hu, Y., and Smyth, G. K. (2009). ELDA: Extreme limiting dilution analysis for comparing depleted and enriched populations in stem cell and other assays. J Immunol Methods *347*, 70-78.

Hutter, G., Nickenig, C., Garritsen, H., Hellenkamp, F., Hoerning, A., Hiddemann, W., and Dreyling, M. (2004). Use of polymorphisms in the noncoding region of the human mitochondrial genome to identify potential contamination of human leukemia-lymphoma cell lines. Hematol J *5*, 61-68.

Klier, M., Anastasov, N., Hermann, A., Meindl, T., Angermeier, D., Raffeld, M., Fend, F., and Quintanilla-Martinez, L. (2008). Specific lentiviral shRNA-mediated knockdown of cyclin D1 in mantle cell lymphoma has minimal effects on cell survival and reveals a regulatory circuit with cyclin D2. Leukemia *22*, 2097-2105.

Liao, Y., Smyth, G. K., and Shi, W. (2014). featureCounts: an efficient general purpose program for assigning sequence reads to genomic features. Bioinformatics *30*, 923-930.

Liem, N. L. M., Papa, R. A., Milross, C. G., Schmid, M. A., Tajbakhsh, M., Choi, S., Ramirez, C. D., Rice, A. M., Haber, M., Norris, M. D.*, et al.* (2004). Characterization of childhood acute lymphoblastic leukemia xenograft models for the preclinical evaluation of new therapies. Blood *103*, 3905-3914.

Love, M. I., Huber, W., and Anders, S. (2014). Moderated estimation of fold change and dispersion for RNA-seq data with DESeq2. Genome Biol *15*, 550.

Macosko, E. Z., Basu, A., Satija, R., Nemesh, J., Shekhar, K., Goldman, M., Tirosh, I., Bialas, A. R., Kamitaki, N., Martersteck, E. M.*, et al.* (2015). Highly Parallel Genome-wide Expression Profiling of Individual Cells Using Nanoliter Droplets. Cell *161*, 1202-1214.

Nombela-Arrieta, C., Pivarnik, G., Winkel, B., Canty, K. J., Harley, B., Mahoney, J. E., Park, S. Y., Lu, J., Protopopov, A., and Silberstein, L. E. (2013). Quantitative imaging of haematopoietic stem and progenitor cell localization and hypoxic status in the bone marrow microenvironment. Nat Cell Biol *15*, 533-543.

Parekh, S., Ziegenhain, C., Vieth, B., Enard, W., and Hellmann, I. (2016). The impact of amplification on differential expression analyses by RNA-seq. Scientific reports *6*, 25533.

Picelli, S., Bjorklund, A. K., Faridani, O. R., Sagasser, S., Winberg, G., and Sandberg, R. (2013). Smart-seq2 for sensitive full-length transcriptome profiling in single cells. Nat Methods *10*, 1096-1098.

Rau, A., Gallopin, M., Celeux, G., and Jaffrezic, F. (2013). Data-based filtering for replicated high-throughput transcriptome sequencing experiments. Bioinformatics *29*, 2146-2152.

Risso, D., Ngai, J., Speed, T. P., and Dudoit, S. (2014). Normalization of RNA-seq data using factor analysis of control genes or samples. Nat Biotechnol *32*, 896-902.

Sedlazeck, F. J., Rescheneder, P., and von Haeseler, A. (2013). NextGenMap: fast and accurate read mapping in highly polymorphic genomes. Bioinformatics *29*, 2790-2791.

Soumillon, M., Cacchiarelli, D., Semrau, S., Oudenaarden, A. v., and Mikkelsen, T. S. (2014). Characterization of directed differentiation by high-throughput single-cell RNA-Seq. bioRxiv.

Subramanian, A., Tamayo, P., Mootha, V. K., Mukherjee, S., Ebert, B. L., Gillette, M. A., Paulovich, A., Pomeroy, S. L., Golub, T. R., Lander, E. S., and Mesirov, J. P. (2005). Gene set enrichment analysis: a knowledge-based approach for interpreting genome-wide expression profiles. Proc Natl Acad Sci U S A *102*, 15545-15550.
