# Supplementary material for: Characterization of Rare, Dormant, and Therapy-Resistant Cells in Acute Lymphoblastic Leukemia
Source: Cancer Cell. 2016 Dec 12;30(6):849–62. doi: 10.1016/j.ccell.2016.11.002 (PMC5156313; doi:10.1016/j.ccell.2016.11.002)
Supplement: Document S2. Article plus Supplemental Information [file mmc6.pdf]

Cancer Cell

Article

Characterization of Rare, Dormant, and Therapy-Resistant Cells in Acute Lymphoblastic Leukemia

Graphical Abstract

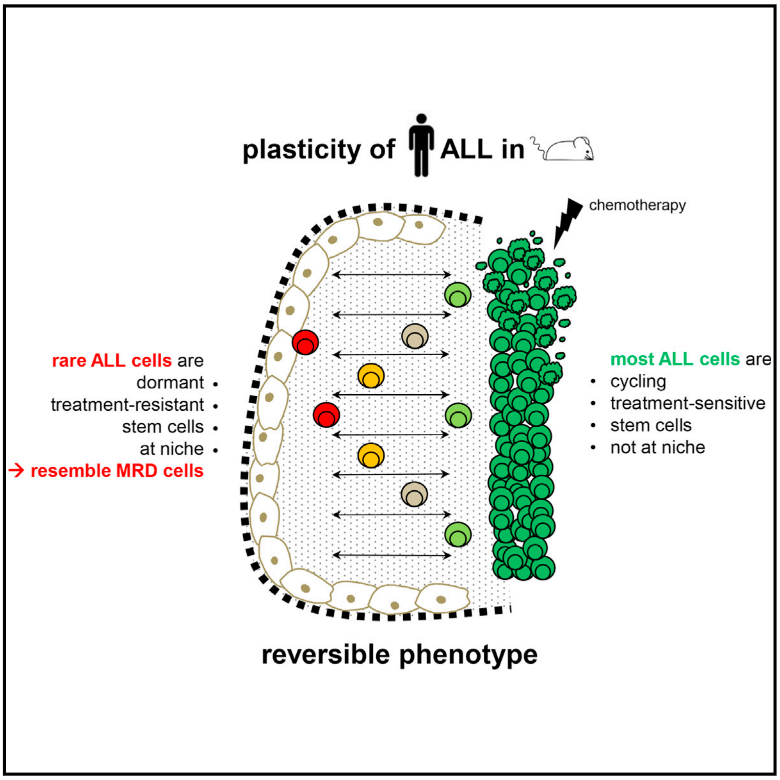

Authors

Sarah Ebinger, Erbey Ziya Özdemir, Christoph Ziegenhain, ..., Wolfgang Enard, Olivier Gires, Irmela Jeremias

Correspondence

irmela.jeremias@helmholtz-muenchen.de

In Brief

Ebinger et al. identify a rare subpopulation of acute lymphoblastic leukemia (ALL) cells that have the combined properties of long-term dormancy, treatment resistance, and leukemia initiation. RNA sequencing results show that these cells are similar to ALL cells isolated from patients at minimal residual disease.

Highlights

- Patients' ALL cells growing in mice contain a rare unfavorable subpopulation
- Unfavorable cells display treatment resistance, dormancy, and stemness
- Unfavorable cells mimic patients' primary cells at minimal residual disease
- Retrieving unfavorable cells from their environment sensitizes them for treatment

Accession Numbers

GSE83142

Ebinger et al., 2016, Cancer Cell *30*, 849–862
December 12, 2016 © 2016 The Author(s). Published by Elsevier Inc.
http://dx.doi.org/10.1016/j.ccell.2016.11.002

Cancer Cell
Article

Characterization of Rare, Dormant, and Therapy-Resistant Cells in Acute Lymphoblastic Leukemia

Sarah Ebinger,[1,15] Erbey Ziya Özdemir,[1,15] Christoph Ziegenhain,[2,15] Sebastian Tiedt,[1,15] Catarina Castro Alves,[1,15] Michaela Grunert,[1] Michael Dworzak,[3] Christoph Lutz,[4] Virginia A. Turati,[5] Tariq Enver,[5] Hans-Peter Horny,[6] Karl Sotlar,[6] Swati Parekh,[2] Karsten Spiekermann,[7,8] Wolfgang Hiddemann,[7,8] Aloys Schepers,[1] Bernhard Polzer,[9] Stefan Kirsch,[9] Martin Hoffmann,[9] Bettina Knapp,[10] Jan Hasenauer,[10,11] Heike Pfeifer,[12] Renate Panzer-Grümayer,[3] Wolfgang Enard,[2] Olivier Gires,[13] and Irmela Jeremias[1,8,14,16,*]

[1]Department of Gene Vectors, Helmholtz Zentrum München, German Center for Environmental Health (HMGU), 81377 Munich, Germany
[2]Anthropology and Human Genomics, Department Biology II, Faculty of Biology, Ludwig-Maximilians-Universität München, 82152 Martinsried, Germany
[3]Children's Cancer Research Institute and St. Anna Kinderspital, Department of Pediatrics, Medical University of Vienna, 1090 Vienna, Austria
[4]Department of Medicine V, University of Heidelberg, 69120 Heidelberg, Germany
[5]University College London Cancer Institute, London WC1E, UK
[6]Institute of Pathology, Ludwig-Maximilians-Universität München, 80337 Munich, Germany
[7]Department of Internal Medicine III, University Hospital Grosshadern, Ludwig-Maximilians-Universität München, 81377 Munich, Germany
[8]German Consortium for Translational Cancer Research (DKTK), Partnering Site, Munich, 81377 Munich, Germany
[9]Project Group Personalized Tumor Therapy, Fraunhofer Institute for Toxicology and Experimental Medicine ITEM, 93053 Regensburg, Germany
[10]Institute of Computational Biology, Helmholtz Zentrum München, German Center for Environmental Health (HMGU), 85764 Neuherberg, Germany
[11]Department of Mathematics, Technische Universität München (TUM), 85748 Munich, Germany
[12]Department of Medicine, Hematology and Oncology, Goethe University, 60590 Frankfurt, Germany
[13]Department of Otorhinolaryngology, Head and Neck Surgery, Grosshadern Medical Center, Ludwig-Maximilians-Universität München, 81377 Munich, Germany
[14]Department of Pediatrics, Dr. von Hauner Children's Hospital, Ludwig Maximilians University München, 80337 Munich, Germany
[15]Co-first author
[16]Lead Contact
*Correspondence: irmela.jeremias@helmholtz-muenchen.de
http://dx.doi.org/10.1016/j.ccell.2016.11.002

SUMMARY

Tumor relapse is associated with dismal prognosis, but responsible biological principles remain incompletely understood. To isolate and characterize relapse-inducing cells, we used genetic engineering and proliferation-sensitive dyes in patient-derived xenografts of acute lymphoblastic leukemia (ALL). We identified a rare subpopulation that resembled relapse-inducing cells with combined properties of long-term dormancy, treatment resistance, and stemness. Single-cell and bulk expression profiling revealed their similarity to primary ALL cells isolated from pediatric and adult patients at minimal residual disease (MRD). Therapeutically adverse characteristics were reversible, as resistant, dormant cells became sensitive to treatment and started proliferating when dissociated from the in vivo environment. Our data suggest that ALL patients might profit from therapeutic strategies that release MRD cells from the niche.

Significance

After initially successful chemotherapy, relapse frequently jeopardizes the outcome of cancer patients. To improve the prognosis of ALL patients, treatment strategies that eliminate tumor cells at minimal residual disease (MRD) and prevent relapse are required. Toward a better understanding of the underlying biology, we established preclinical mouse models mimicking MRD and relapse in patients. Primary and surrogate MRD cells shared major similarities in expression profiles, demonstrating the suitability of our model. MRD cells revealed major functional plasticity in vivo and treatment resistance was reversible; MRD cells became sensitive toward treatment once released from their in vivo environment. Effective therapeutic strategies might aim at dissociating persistent cells from their protective niche to prevent relapse in ALL patients.

© 2016 The Author(s). Published by Elsevier Inc.
This is an open access article under the CC BY license (http://creativecommons.org/licenses/by/4.0/).

INTRODUCTION

Relapse represents a major threat for patients with cancer. After initially successful treatment, rare tumor cells might survive and re-initiate the malignant disease with dismal outcome. Acute lymphoblastic leukemia (ALL) is associated with poor prognosis in infants and adult patients and is the most frequent malignancy in children (Inaba et al., 2013). In many patients, the majority of ALL cells respond to chemotherapy but a minority display resistance, survive therapy, and cause relapse with poor outcome (Gokbuget et al., 2012).

Despite its clinical importance, basic biologic conditions underlying relapse remain partially elusive. For example, it is unclear whether relapse-inducing cells exist before onset of treatment or develop as result of therapy, and whether permanent or reversible characteristics determine relapse-inducing cells (Kunz et al., 2015). Of translational importance, understanding basic mechanisms opens perspectives for effective therapies to eradicate relapse-inducing cells.

Relapse-inducing cells, by their clinical definition, self-renew and give rise to entire tumors indicating tumor-initiating potential, a typical characteristic of cancer stem cells (Essers and Trumpp, 2010). In numerous tumor entities including acute myeloid leukemia, cancer stem cells were identified as a biologically distinct subpopulation that displays specific surface markers, has leukemia-inducing potential in mice, and gives rise to a hierarchy of descendant cells that lack such properties (Bonnet and Dick, 1997; Visvader and Lindeman, 2008). In ALL, however, many different subpopulations display stem cell properties; neither a stem cell hierarchy nor phenotypic markers defining stem cells could be identified (Kong et al., 2008; le Viseur et al., 2008; Rehe et al., 2013). Thus, up to now, stemness represents an insufficient criterion to define the subpopulation of relapse-inducing cells in ALL.

An additional feature of relapse-inducing cells is their treatment resistance, as, again by definition, they survive chemotherapy and eventually give rise to relapse with decreased chemosensitivity. Resistance against chemotherapy is closely related to dormancy as chemotherapy mainly targets proliferation-associated processes that are inactive in dormant cells (Clevers, 2011; Zhou et al., 2009). Dormant cells, by definition, do not divide or divide very slowly over prolonged periods of time, might survive chemotherapy, persist in minimal residual disease (MRD), and give rise to relapse (Schillert et al., 2013; Schrappe, 2014). Indeed, an increased frequency of non-dividing tumor cells has been described in patients after chemotherapy for defined subtypes of ALL (Lutz et al., 2013).

So far, technical obstacles have hampered characterizing phenotypic and functional features of relapse-inducing cells in ALL in detail. Established ALL cell lines represent inappropriate models as they display continuous proliferation. In patients, relapse-inducing cells are very rare and defining cell surface markers that reliably identify these rare ALL cells from the multiplicity of normal bone marrow cells remains intricate, at least in certain ALL subtypes (Hong et al., 2008; Ravandi et al., 2016). Moreover, primary ALL cells do not grow ex vivo, disabling their amplification in culture.

An attractive possibility to experimentally study patients' tumor cells in vivo is the patient-derived xenograft (PDX) model, which uses immuno-compromised mice to expand tumor cells from patients (Kamel-Reid et al., 1989). As shown previously, PDX ALL cells retain important characteristics of primary ALL cells (Castro Alves et al., 2012; Schmitz et al., 2011; Terziyska et al., 2012). While PDX models are mostly used for preclinical treatment trials (Gao et al., 2015; Townsend et al., 2016), we used them here to study relapse-inducing cells in ALL.

RESULTS

To characterize the challenging subpopulation of relapse-inducing cells in ALL, we used the individualized xenograft mouse model as a preclinical model, molecular cell marking as an unbiased approach, and in vivo dormancy as a functional benchmark. To mimic the heterogeneity of ALL, samples from nine different ALL patients were studied including children and adults, B cell precursor-ALL and T-ALL, first diagnosis, and relapse (Table S1).

Molecular Marking Allows Unbiased, Sensitive Isolation of Rare PDX ALL Cells

To study ALL growth starting very early after disease onset in the PDX mouse model, the technical challenge consisted in reliably enriching very low numbers of human ALL cells from mouse bone marrow. As expression levels of endogenous surface antigens across potentially relevant, but yet undefined, subpopulations are unknown, we used lentiviral transduction for unbiased molecular marking and in vivo imaging (Figure 1A).

PDX ALL cells were lentivirally transduced to express a luciferase for in vivo imaging (Terziyska et al., 2012), an artificial antigen (truncated nerve growth factor receptor [NGFR]) for magneto-activated cell sorting (Fehse et al., 1997) and a red fluorochrome for cell sorting by flow cytometry (Figures S1A and S1B). Transgenes allowed effective and reliable enrichment of minute numbers of PDX cells from mouse bone marrow in this two-step procedure. Quantification of PDX cells isolated with the magnetic-activated cell sorting (MACS)/fluorescence-activated cell sorting approach closely correlated with other methods monitoring leukemic proliferation, such as in vivo imaging and flow cytometry-based quantification of leukemia cells (Figure S1C). Quality controls showed that the procedure was highly efficient and reliable with minor cell loss (Table S2).

The procedure enabled addressing basic questions with translational potential in ALL biology. Homing capacity of PDX cells to mouse bone marrow differed by more than two orders of magnitude between the nine samples studied (Figure 1B). Homing efficiency decreased significantly when smaller cell numbers were injected (Figure S1D). These data argue in favor of sample-specific characteristics determining homing, and against the presence of a preformed, fixed number of leukemia homing sites within the niche. Spontaneous growth of PDX ALL cells in mouse bone marrow was logarithmic over the first 2 weeks of in vivo growth (Figures 1C and S1C). Growth slowed down thereafter and as early as at 10% blasts in bone marrow, when space restriction appears unlikely to be causative. Model selection indicated overall logistic growth which is typical for insufficient nutrient supply (Figure S1E). Thus, PDX ALL cells show sample-specific homing followed by logistic growth in mouse bone marrow.

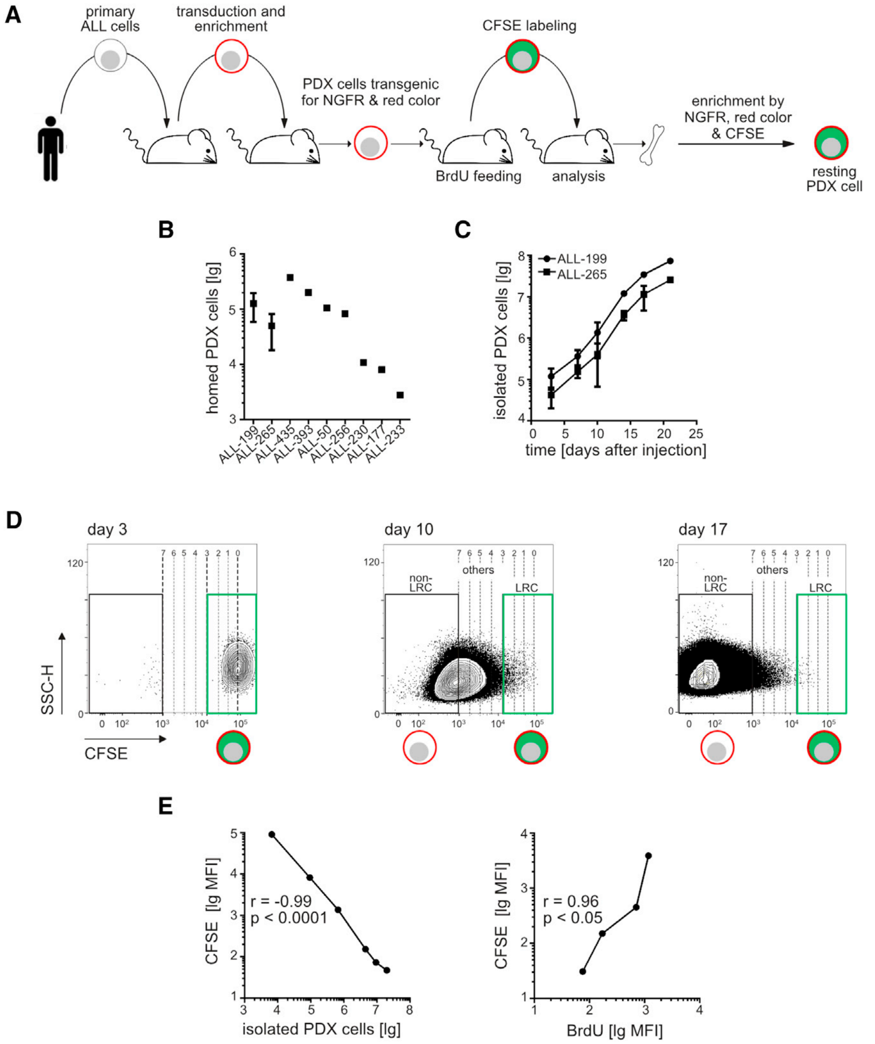

Figure 1. CFSE Staining Allows Reliable Monitoring of PDX ALL Growth in Mice

(A) Experimental procedure of generating PDX ALL cells expressing several transgenes, staining with CFSE, and enriching rare transgenic, CFSE-stained PDX cells from mouse bone marrow.

(B) Of each PDX sample, 10^7 triple transgenic PDX cells were injected intravenously into mice and re-isolated from the bone marrow 3 days later; each dot represents data from one mouse, except that a mean of eight mice plus SE is shown for samples ALL-199 and ALL-265.

(C) 10^7 CFSE-stained PDX cells/mouse were injected and PDX cells were quantified in up to 11 mice per time point; shown is mean and SE.

(D) Gating strategy defining LRC, non-LRC, and others. MFI of CFSE at the start of the experiment (3 days after cell injection) was divided by factor 2 to model bisections; upon no more than three bisections, cells were considered as LRC, upon more than seven bisections as non-LRC; intermediate cells were considered as others.

(E) Similar experiment as in (C), except that the donor mouse was fed with BrdU in the last 7 days before cell harvesting. Each dot represents data from one mouse.

See also Figure S1, Tables S1, and Table S2.

CFSE Staining Allows Reliable Monitoring of PDX ALL Growth in Mice

Proliferation-dependent dyes such as bromodeoxyuridine (BrdU) and carboxyfluorescein diacetate succinimidyl ester (CFSE) remain stable in mice over several months, enabling the characterization of a heterogeneous growth pattern in normal hematopoiesis (Takizawa et al., 2011). We adapted the use of these dyes in PDX tumor models. As BrdU staining requires the permeabilization and destroying of cells, fluorescent CFSE was mainly used as it allows flow cytometric enrichment of living cells for functional experiments including re-transplantation. Loss of CFSE was used to distinguish subpopulations of slowly and rapidly growing cells (Figures 1D and S1F) that were called label-retaining cells (LRC) and non-label-retaining cells (non-LRC), respectively (Takizawa et al., 2011). LRC were defined as those cells that had undergone at most three CFSE bisections resembling cell divisions (see the Supplemental Experimental Procedures for details). Loss of CFSE tightly correlated with increase in PDX cell numbers and loss of BrdU (Figures 1E and S1G) and confirmed that PDX ALL cells grow in vivo, but not ex vivo (Figure S1H). Thus, CFSE staining represents a reliable approach to monitor proliferation of PDX ALL cells in mice.

A Rare, Long-Term Dormant Subpopulation Exists in ALL PDX Cells

Importantly, CFSE staining disclosed the existence of a rare fraction of PDX ALL cells that hardly divided over prolonged periods of time (Figure 2A). LRC, by definition, had undergone no more than three cell divisions within 21 days, during which the leukemia burden had risen by several orders of magnitude so that mice would succumb to leukemia within a few days. In all nine PDX ALL samples studied, LRC were identified after prolonged periods of leukemic growth; (Figures 2B and S2A).

Thus, similarly to normal hematopoiesis (Trumpp et al., 2010), PDX ALL contains a rare subpopulation of LRC. LRC might resemble the dormant tumor cells described in ALL patients (Figure S2B) (Lutz et al., 2013). As an advantage over work with primary cells, our preclinical approach allows repetitive work on pure, vivid LRC, which gave us the chance to functionally and phenotypically characterize this interesting population.

LRC Localize to the Endosteum, but Are Not Enriched for Stem Cells

Both normal hematopoietic stem cells and leukemia stem cells were reported to preferentially localize close to the endosteum, where a supportive niche might exist (Morrison and Spradling, 2008). We also found that LRCs preferentially localized close to the endosteum (Figures 3A–3C and S3), suggesting that they might use the same niche as normal hematopoietic stem cells and cancer stem cells.

We therefore asked whether LRC might resemble cancer stem cells. To compare leukemia-initiating potential between LRC and

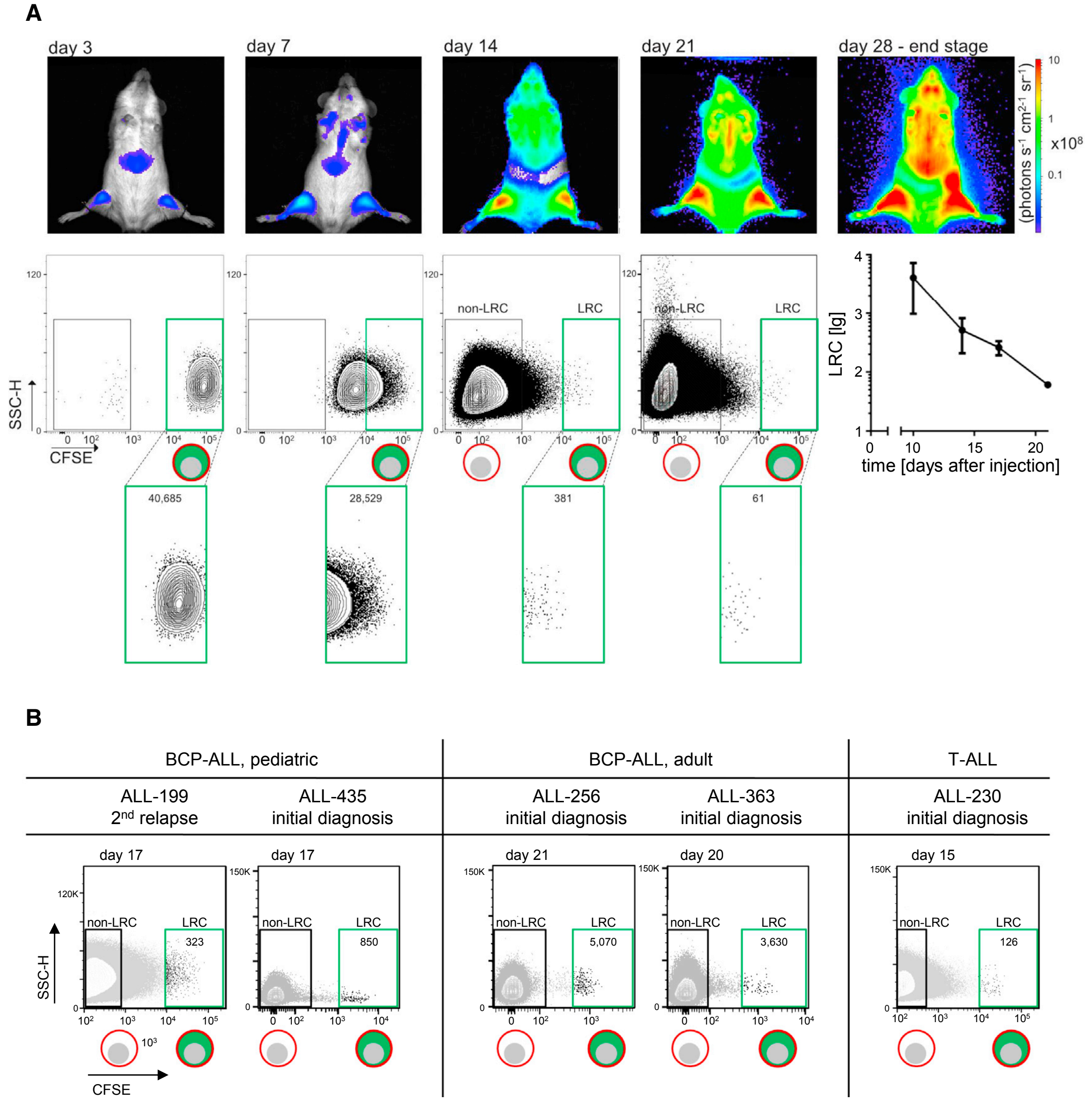

Figure 2. A Rare, Long-Term Dormant Subpopulation Exists in ALL PDX Cells

(A) 10^7 CFSE-stained PDX ALL-265 cells were injected into each of six mice; bioluminescence in vivo imaging was performed prior to quantifying LRC in one mouse per time point; LRC numbers are indicated and summarized in the line graph as a mean of up to ten mice ± SE.

(B) Identification of LRC in PDX cells from all different ALL patients. Experiments were performed as in (A).

See also Figure S2.

non-LRC, we performed limiting dilution transplantation assays and monitored engraftment by bioluminescence in a total of 83 mice (Table S3). To our surprise, we found highly similar stem cell frequencies in LRC and non-LRC and similar engraftment rates after transplantation of, e.g., ten cells per mouse (Figure 3D). The 95% confidence interval of the estimated frequency of leukemia-inducing cells ranged between 1/19 and 1/84 cells for LRC and between 1/40 and 1/179 cells in non-LRC of ALL-265 (Table S3). Similar findings were obtained for ALL-199 (Table S3). Thus, although only LRC display typical characteristics of stem cells such as reduced proliferation rate and localization close to the endosteum, LRC and non-LRC exhibited similar leukemia-initiating potential.

LRC Survive Systemic Drug Treatment In Vivo

Dormant cells are known for their resistance against drug treatment, complicating elimination by anti-cancer therapy (Essers and Trumpp, 2010). We compared in vivo drug response of LRC and non-LRC by transplanting CFSE-labeled PDX ALL cells, treating mice with systemic chemotherapy on day 7 and analyzing surviving LRC and non-LRC on day 10 (Figure 4A). Chemotherapy reduced the overall leukemic burden by over 90% (Figures 4B and S4A) and eradicated most non-LRC. As a prominent difference, most LRC survived chemotherapy so that LRC increased in relative proportions (Figures 4C–4E and S4B–S4D). A 10- to 100-fold less efficient elimination of LRC compared with non-LRC became obvious across all PDX ALL

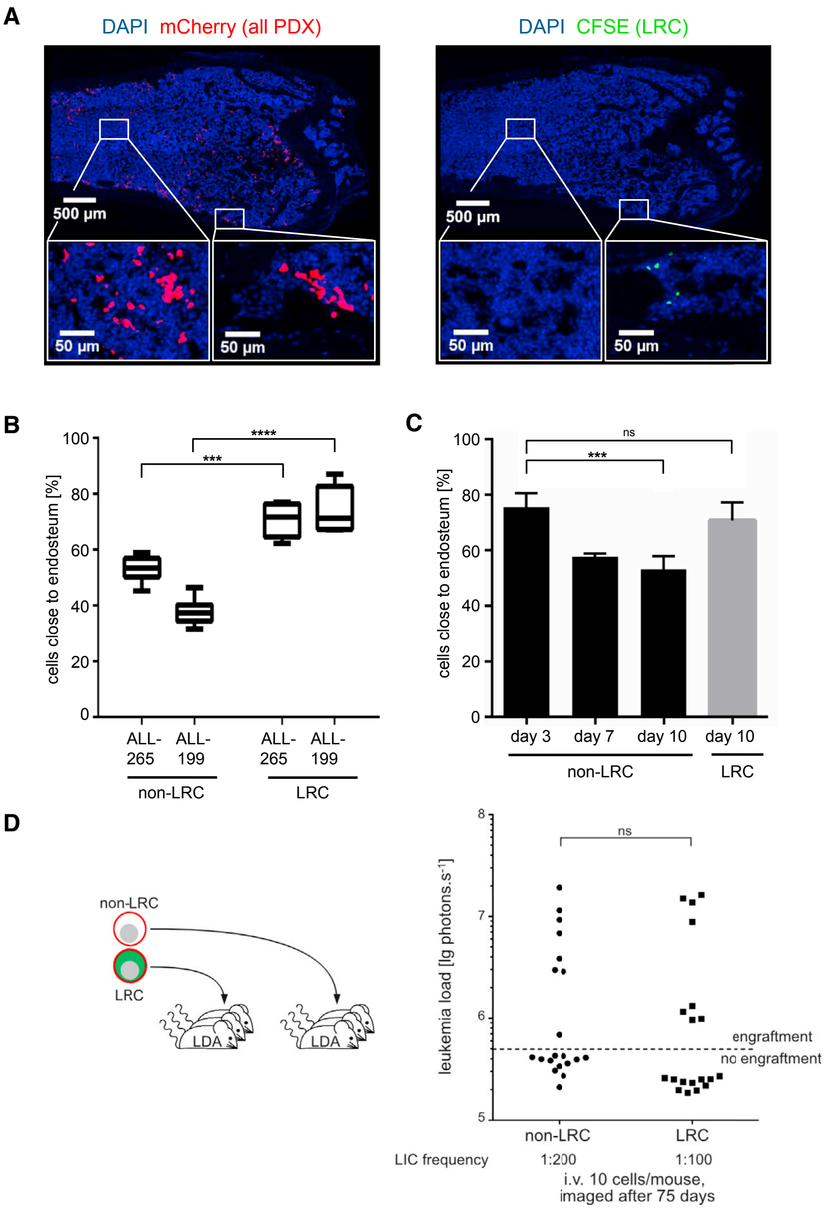

Figure 3. LRC Localize to the Endosteum, but Are Not Enriched for Stem Cells

(A) Immunohistochemistry of consecutive mouse bone marrow femur sections 10 days after injection of CFSE-stained PDX ALL-265 cells; mCherry (red; left panel) indicates all PDX cells, CFSE (green; right panel) indicates LRC.

(B) All sections from day 10 were quantified defining the endosteal region as less than 100 µm from bone matrix; shown is the median with upper/lower quartile and maximum/minimum of two to three sections from two femurs in two mice per data point; ***$p < 0.001$, ****$p < 0.0001$ by two-tailed unpaired t test.

(C) Kinetic for ALL-265 as mean ± SE; ***$p < 0.01$ by two-tailed unpaired t test.

(D) Ten LRC or non-LRC were injected into each of 39 mice and engraftment was determined by in vivo imaging at day 75; each dot represents one mouse; dashed line represents detection threshold (5×10^5 photons s^{-1}); ns: not significant as determined by two-tailed unpaired t test.

See also Figure S3 and Table S3.

samples tested that were derived from either primary disease or relapse, suggesting that this phenomenon is not restricted to a certain disease stage. Treatment-surviving LRC harbored leukemia-initiating potential as they gave rise to leukemias upon retransplantation at a kinetic similar to that of untreated LRC (Figures 4F and S4E).

Taken together, LRC share the most important functional features that impede the cure of cancer: (1) dormancy, (2) in vivo drug resistance, and (3) leukemia-initiating potential. LRC might thus serve as preclinical surrogate for relapse-inducing cells in ALL.

Expression Profile of LRC Shows Distinct Changes to Non-LRC

We then evaluated whether LRC adequately resemble challenging cells in patients. For a broad, unbiased comparison between LRC and non-LRC, RNA sequencing (RNA-seq) was performed on single cells and bulk populations (Figure 5A). Data from single cells correlated with data from bulk populations and different ALL PDX samples showed similar expression profiles (Figures S5A and S5B). Preliminary expression arrays on pools of 40 LRC and non-LRC showed mainly similar results (data not shown).

Single LRC differed consistently from single non-LRC as revealed by clustering differently expressed genes (Figures 5B and Table S4) and by a principle component analysis of the most variable genes (Figure 5C). Single LRC also had an overall reduced RNA content (Figure S5C), indicating a less active metabolism that is a prerequisite of dormant cells. We combined single-cell and bulk data of all six sample pairs to identify differently expressed genes (Table S5). Enrichment analysis revealed that genes expressed less in LRC were most strongly enriched in cell cycle and DNA replication and that genes more expressed in LRC were most strongly enriched in cell adhesion (Figures 5D, S5D, and Table S6). Hence, expression profiling of single cells and in bulk confirmed the quiescent state of LRC and an LRC signature of at least 2-fold differently expressed genes ranked by their significance (Figures 5E and Table S5) was used for further comparisons.

LRC Resemble MRD Cells in the PDX Mouse Model

Relapse often results from treatment-resistant tumor cells that survive chemotherapy and persist at MRD. MRD cells contain a major fraction of dormant tumor cells (Lutz et al., 2013). Here, we hypothesized that LRC might represent surrogates for MRD cells.

To experimentally test this hypothesis, we established a preclinical model of MRD for ALL-265 and ALL-199. When untreated

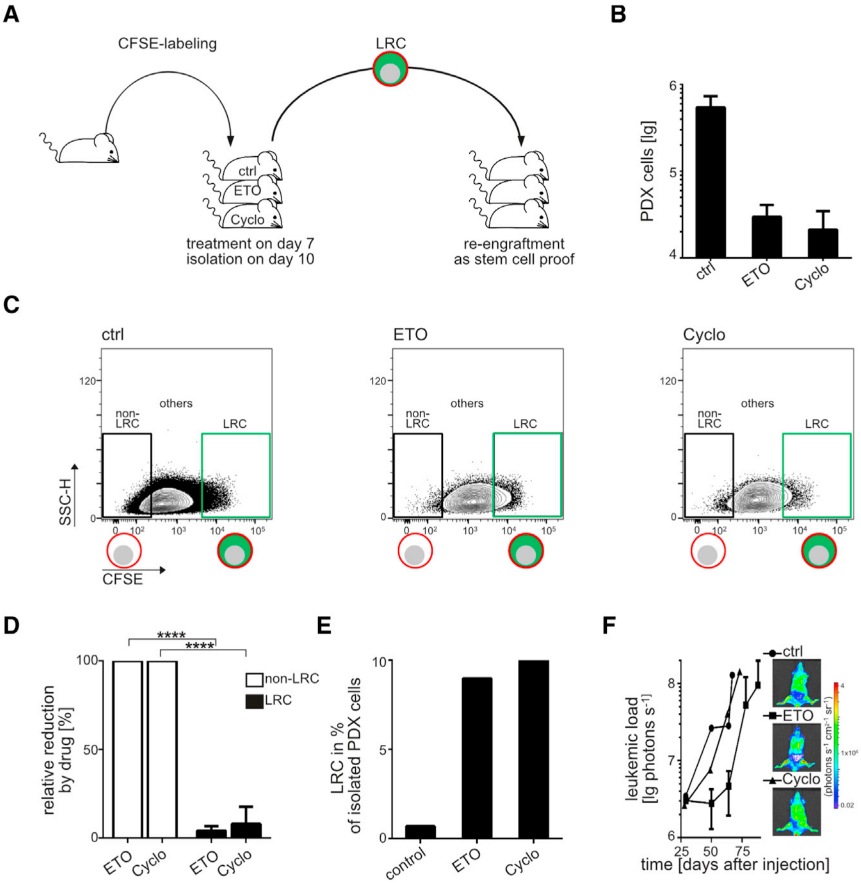

Figure 4. LRC Survive Systemic Drug Treatment In Vivo

(A) Each mouse was injected with 10^7 CFSE-stained ALL-265 PDX cells and treated with buffer, etoposide (ETO, 50 mg/kg, intraperitoneally [i.p.]), or cyclophosphamide (Cyclo, 150 mg/kg, i.p.) on day 7. Mice were euthanized on day 10; LRC were analyzed and re-transplanted into secondary recipients.

(B) Living PDX cells from mice in (A) were quantified and presented as mean of each group (n = 4–5) ± SE.

(C) Original data for one representative mouse per treatment.

(D) Mean of all four to five mice per treatment, depicted as relative drug effect on LRC compared with non-LRC (100%) ± SE; ****p < 0.0001 by two-tailed unpaired t test.

(E) Mean relative proportion of LRC of total PDX cells.

(F) LRC isolated were re-transplanted and mice monitored by in vivo imaging; mean of each group (n = 1–2) ± SE.

See also Figure S4.

control samples were harvested at advanced leukemia, they contained a leukemic burden of ~30% human blasts in mouse bone marrow, mimicking the situation at diagnosis. Remaining mice received a systemic treatment with conventional chemotherapeutic drugs over 2–3 weeks (Figure 6A), which needs careful dosing as supportive therapy is mainly unfeasible in mice. A combination treatment of vincristine and cyclophosphamide reduced tumor burden substantially according to in vivo imaging (Figures 6B, 6C, and S6A). Postmortem analysis revealed that chemotherapy had reduced leukemic burden by more than two orders of magnitude to ~0.1% leukemia cells in bone marrow. This resembled not only complete morphologic, but also complete molecular remission criteria (Figures 6D and S6B). MRD cells revealed relapse-inducing potential as they re-grew in mice when treatment was stopped (Figure S6C).

MRD cells were isolated from mouse bone marrow using expressed transgenes as above, and RNA sequencing of single cells and bulk samples was performed. Resulting transcriptomes showed marked differences between MRD and untreated control cells (Figure S6D). Enrichment analysis revealed significantly reduced expression of MYC and E2F target genes in MRD compared with untreated cells. Genes expressed less in MRD cells were most strongly enriched in cell cycle and DNA replication, while genes expressed more in MRD cells were most strongly enriched in cell adhesion (Figures 6E and S6E). This suggests a dormant phenotype of MRD cells similar to the dormant phenotype seen in LRC (Figure 5D). KEGG pathway analysis highlighted that MRD cells were of dormant nature and expressed increased adhesion molecules (Figure S6E). Indeed, single MRD cells clustered together with single LRC in a principal component analysis separated from non-LRC and cells from untreated mice (Figure S6F). Accordingly, the LRC signature (Figure 5E and Table S5) was strongly enriched in MRD cells and genes in MRD and LRC cells were similarly regulated compared with their respective controls (Figure 6F). This suggests that LRC mimic MRD cells in our preclinical mouse model.

LRC Resemble Primary MRD Cells from Patients

To relate these findings to the clinical situation, expression profiles from primary tumor cells from five children and two adults with B cell precursor (BCP) ALL were profiled at diagnosis and at MRD (Figure 7A and Table S7). Children and adults were treated according to the BFM-2009 and GMALL-0703 protocols, respectively, and MRD cells were enriched by flow cytometry at days 33 and 71 of treatment, respectively. In adults, we chose BCR-ABL-positive ALL and enriched the subpopulation of StemB cells at MRD, as Lutz et al. (2013) had shown that these cells exhibit a dormant phenotype. As dormancy in StemB cells might have persisted for a long period during treatment in patients, LRC might especially resemble StemB cells at MRD. We could obtain single-cell transcriptomes from one patient and one bulk transcriptome from another patient. K-means clustering and principal component analysis revealed that single StemB cells clustered together with single LRC and MRD cells, while single non-LRC clustered together with single untreated control cells (Figures 7B and 7C). The bulk StemB sample was distinct from diagnostic tumor cells of untreated adult patients with BCR-ABL-positive ALL (Figure S7A). Although limited by small cell and sample numbers, the data indicate that LRC resemble

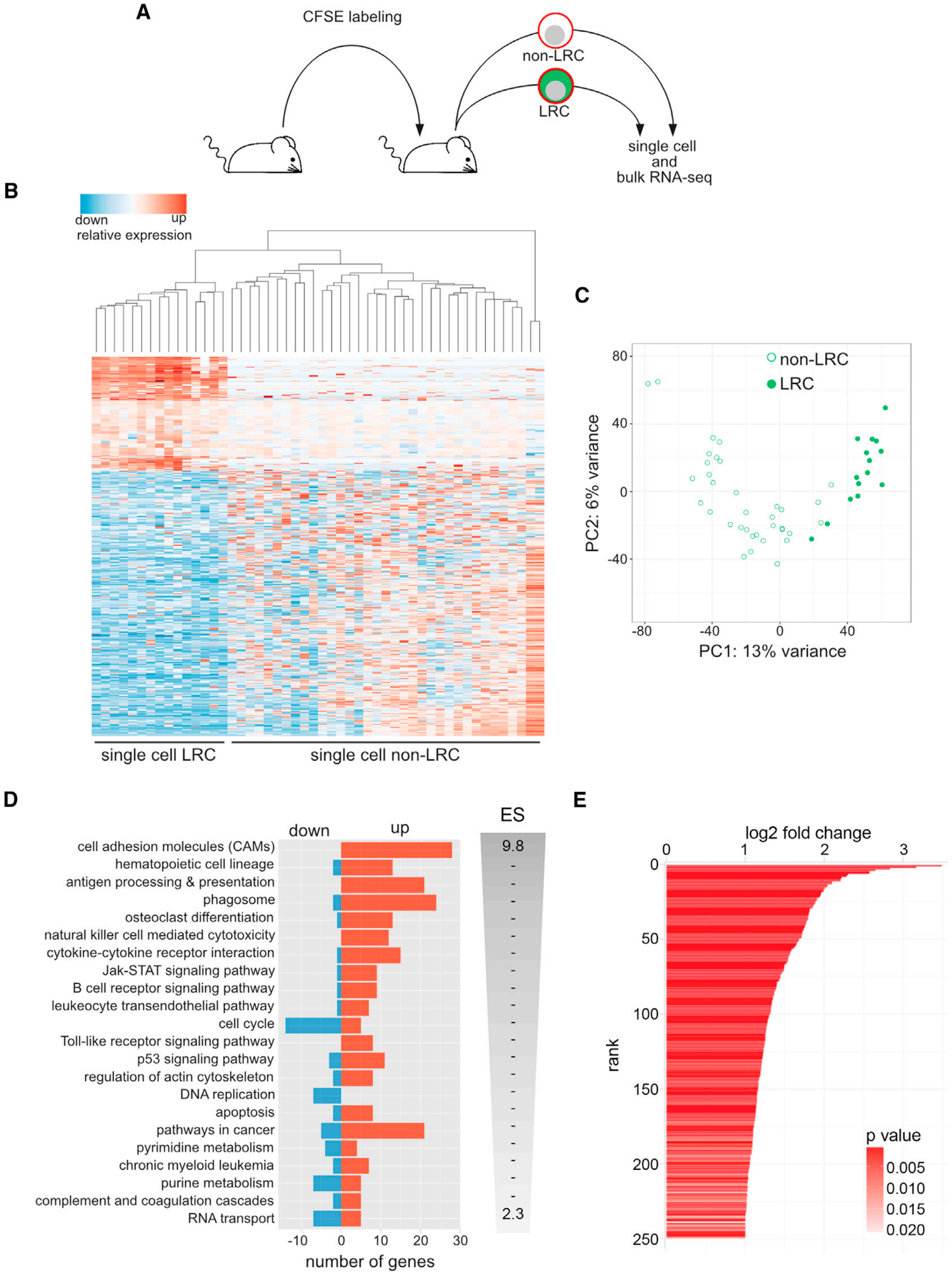

Figure 5. Expression Profile of LRC Shows Distinct Changes to Non-LRC

(A) Fifteen days after transplantation, ALL-265 LRC or non-LRC were isolated and single-cell mRNA-seq was performed in 15 LRC and 35 non-LRC.

(B) Hierarchical clustering and gene expression heatmap across the 500 most differentially expressed genes (false discovery rate [FDR] <0.01) in 15 LRC and 35 non-LRC single cells. Values are plotted relative to the average of non-LRC.

(C) Principal component analysis of the 500 most variable genes in all 50 single cells.

(D) Significantly enriched KEGG pathways (FDR <0.05) as determined by fixed network enrichment analysis (FNEA); bars show the number of significantly up- or downregulated genes in the corresponding pathway and are ordered according to the enrichment score (ES).

(E) LRC signature genes (FDR < 0.05 and log2 fold-change >1) were derived from integrated bulk and single-cell RNA-seq analysis from six animals carrying either ALL-265 or ALL-199 and are shown ranked by fold-change and colored by significance.

See also Figure S5, Tables S4, S5, and S6.

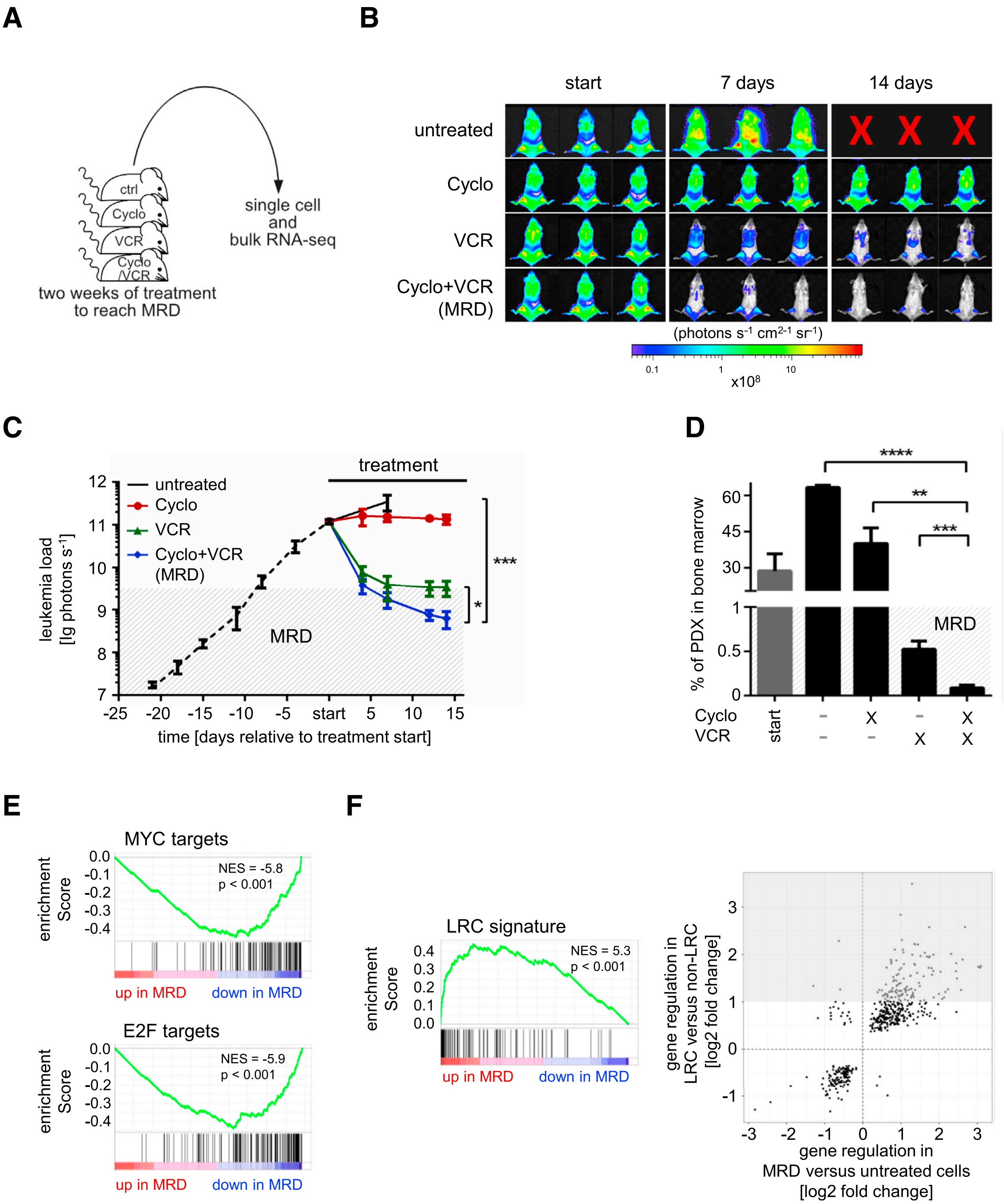

Figure 6. LRC Resemble MRD Cells in the PDX Mouse Model

(A) 10^7 ALL-199 cells were injected into 19 mice; when 30% of bone marrow cells were human, PDX cells were enriched from five mice and used as untreated control samples; cells of one mouse were subjected to single-cell sequencing; the remaining mice received buffer, vincristine (VCR, 0.25 mg/kg; n = 5), cyclophosphamide (Cyclo, 100 mg/kg; n = 3), or a combination thereof (VCR + Cyclo; n = 6) weekly for 2 weeks; when VCR + Cyclo combination treatment had reduced tumor burden to MRD (<1% human cells in bone marrow), PDX cells were enriched and cells of one VCR + Cyclo mouse were subjected to single cell mRNA-seq.

(B) In vivo imaging data of three representative mice per group.

(C) Mean of each group ± SE; *p < 0.05, ***p < 0.001 by two-tailed unpaired t test; mice receiving buffer had to be euthanized after 1 week of treatment due to end-stage leukemia.

(D) Percentage of PDX ALL cells in mouse bone marrow as determined by flow cytometry postmortem as mean ± SE; **p < 0.01, ***p < 0.001, ****p < 0.0001 by two-tailed unpaired t test.

(E) MRD cells show reduced expression of MYC- and E2F-target genes in gene set enrichment analysis (GSEA) (Liberzon et al., 2015).

(legend continued on next page)

the dormant subpopulation of StemB cells in adult ALL patients at MRD.

This is also supported when comparing the LRC profiles with further published transcriptomes. Genes differently expressed in CD34-positive chronic myeloid leukemia cells (Graham et al., 2007), in leukemia stem cells (Saito et al., 2010), in hematopoietic stem cells (Eppert et al., 2011; Georgantas et al., 2004), as well as in pediatric ALL cells with high risk of relapse (Kang et al., 2010) were all significantly enriched in LRC versus non-LRC cells (Figures 7D, S7B, and S7C).

To further analyze the similarity of LRC to MRD cells from patients, we generated bulk transcriptomes of primary samples from five children with BCP-ALL before the onset of treatment and three matched MRD samples collected 33 days after the onset of treatment. Expression profiles differed significantly between diagnosis and MRD (Figure 7E and Table S8) and MRD cells regulated genes in the same direction as LRC compared with their respective controls, as revealed by a significant overlap of up- and downregulated genes (hypergeometric test, $p = 1.9 \times 10^{-23}$) and by a significant enrichment of the LRC signature ($p < 0.001$; Figure 7F). Finally, we combined these transcriptomes with all bulk samples isolated from the LRC and MRD mouse models and analyzed them unsupervised in a principal component analysis (Figure 7G). The first principal component separated all dormant and drug-resistant cells (PDX-LRC, PDX-MRD, and primary MRD) from all control cells (PDX-non-LRC, PDX untreated, and primary diagnosis).

In summary, we show that a distinct subpopulation of LRC exists in our ALL PDX model that combines the unfavorable characteristics of stemness, drug resistance, and dormancy. These LRC show high similarities to MRD cells in our mouse model and to MRD cells in ALL patients. Hence, LRC might represent preclinical surrogates for relapse-inducing cells in patients and could be used to develop therapeutic strategies to prevent relapse.

Release from the Environment Induces Proliferation in LRC

As the first step toward therapies, we studied whether unfavorable drug resistance and dormancy represented permanent or reversible features in LRC. Dormancy and drug resistance might exist as genuine, constant biological characteristics of a special ALL subpopulation or as reversible functional phenotypes of putatively every ALL cell depending on the context.

To address this question, LRC and non-LRC were dissociated from their environment, isolated, and re-transplanted into recipient mice (Figures 8A and S8A). When non-LRC were re-stained with CFSE and re-transplanted at high numbers, they gave rise to an identical LRC population as re-transplanted bulk cells (Figures 8B and S8A); transplantation of high cell numbers of LRC was impossible, as only low numbers of LRC can be recovered from mice. When low cell numbers were re-transplanted, LRC, non-LRC, and bulk cells initiated identical leukemic growth in mice as monitored by bioluminescence in vivo imaging (Figures 8C and S8A). These data indicate that dormancy represents a reversible feature of LRC, as LRC lose their dormant nature once they are retrieved from their specific environment and transferred into a different surrounding.

Release from the Environment Sensitizes LRC and MRD Cells for Drug Treatment

As dormancy emerged as a reversible phenotype, we asked whether drug resistance might be equally reversible. Isolated LRC and non-LRC or MRD and previously untreated cells from the PDX mouse model were treated ex vivo with common ALL chemotherapy drugs or drug controls. Here, the technical challenge lay in the very minor cell numbers of LRC and MRD that can be isolated from mice and used for ex vivo experiments (Figure S8B). Co-culture with feeder cells resembling bone marrow stroma reduced drug response in all samples, suggesting the influence of the bone marrow environment on drug resistance (Figures S8C–S8F) (Tesfai et al., 2012). Ex vivo, neither LRC nor MRD cells displayed increased drug resistance compared with their respective controls (Figures 8D and S8G).

Taken together, LRC and MRD cells showed a marked gain in drug sensitivity ex vivo compared with in vivo after isolation from the bone marrow environment. Both LRC and MRD cells lost their enhanced drug resistance, distinguishing them from non-LRC or untreated cells, once they were retrieved from their in vivo environment and cultured ex vivo (Figure 8E). Dormancy was reversible in LRC and drug resistance was reversible in both LRC and MRD cells. As LRC might represent surrogates for relapse-inducing cells in patients, our data suggest that the interaction between LRC and their environment represents an attractive therapeutic target for preventing relapse. Relapse-inducing cells might gain sensitivity toward treatment once mobilized from their in vivo environment.

DISCUSSION

The present work aimed at a better understanding of the cells that induce relapse in ALL and thereby limit prognosis of patients. We identified a rare, long-term dormant subpopulation termed LRC exhibiting the adverse characteristics of dormancy, in vivo drug resistance, and leukemia-initiating properties. LRC highly resemble primary MRD cells from adult and pediatric patients with ALL. MRD cells require preferential eradication by anti-leukemia treatment. LRC in preclinical models can now be used as surrogates for relapse-inducing cells in patients for developing therapies to prevent relapse. Upon removal from their in vivo environment, LRC lost dormancy and drug resistance, suggesting a reversible nature of adverse characteristics and an important role for the interaction between ALL and the environment. The data suggest that drug resistance and dormancy are linked and represent an acquired stem-like phenotype. Our data imply developing treatment approaches that dissociate ALL cells from their protective niche to sensitize them toward anti-leukemia treatment.

(F) GSEA was performed comparing LRC signature with transcriptomes of MRD versus untreated cells (mean of data for ALL-199; left panel). Scatterplot of fold-changes for genes differentially expressed (FDR < 0.05) between both LRC versus non-LRC and MRD versus untreated control cells; grey area indicates LRC signature (right panel).
See also Figure S6.

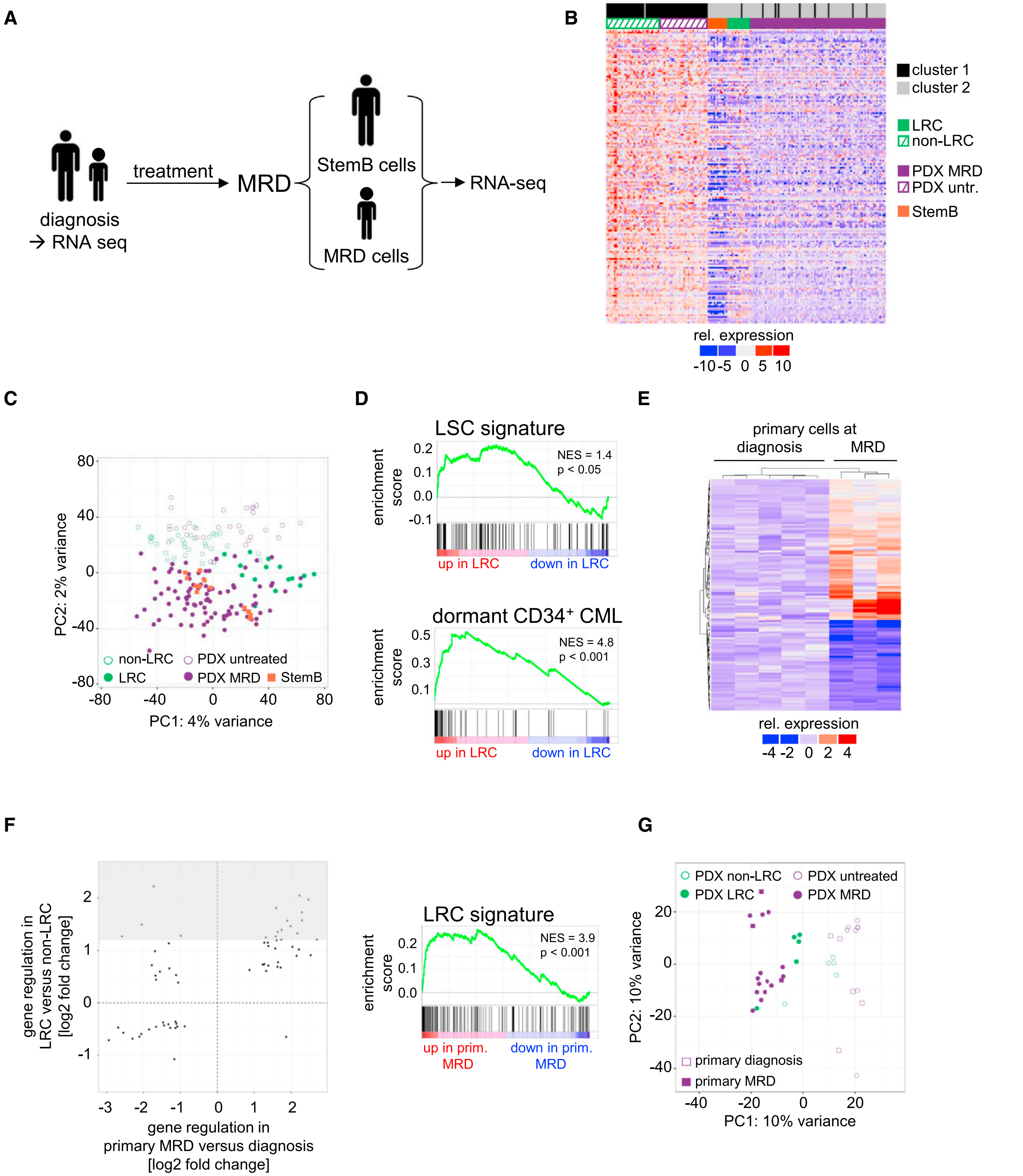

Figure 7. LRC Resemble Primary MRD Cells from Patients

(A) Adult or pediatric ALL patients were treated according to GMALL-0703 or BFM-2009 protocols for 71 or 33 days, respectively; at MRD, the subgroup of StemB cells (in samples from adults) or all remaining ALL cells (in samples from children) were enriched out of normal bone marrow; cells at diagnosis and at MRD were subjected to RNA-seq.

(B) K-means clustering of gene expression values of 167 highly differentially expressed genes (FDR < 0.001) of all data from single cells.

(C) Principal-component analysis (PCA) of single cell transcriptomes using all shared expressed genes; each symbol indicates a single cell.

(D) GSEA comparing the LRC signature with signatures of leukemia stem cells (Saito et al., 2010) and dormant CD34-positive chronic myeloid leukemia (CML) (Graham et al., 2007).

(legend continued on next page)

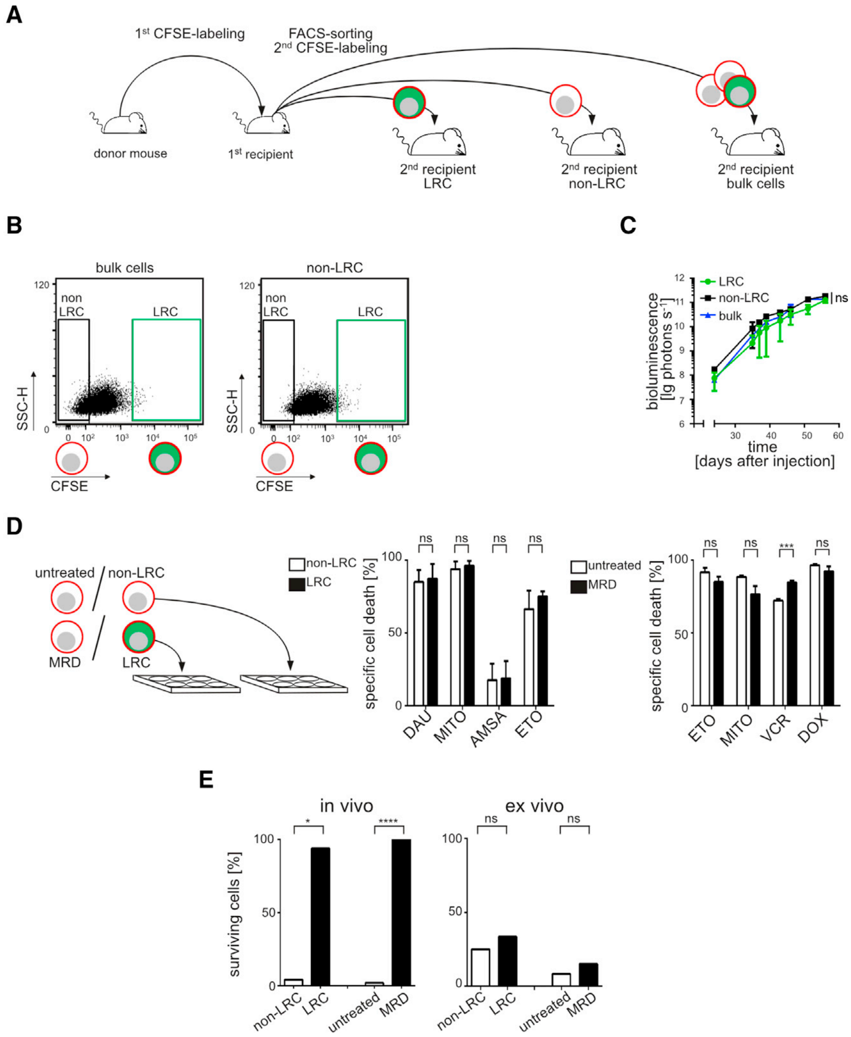

Figure 8. Release from the Environment Induces Proliferation in LRC and Sensitizes LRC and MRD Cells toward Drug Treatment

(A) From a first recipient mouse carrying CFSE-stained ALL-199 cells, LRC, non-LRC, and bulk cells were obtained at day 10; bulk cells and non-LRC were re-labeled with CFSE, re-transplanted in second recipient mice at high numbers, and re-analyzed at day 10 using flow cytometry; bulk cells, LRC, and non-LRC were re-transplanted at low numbers into groups of mice and leukemia growth was monitored over time.

(B) CFSE staining at day 10 in secondary recipient mice receiving high cell numbers.

(C) Growth curve in secondary recipients; mean ± SE; ns, no statistical significance by Kruskal-Wallis test and Dunn's multiple comparison test. One out of two independent experiments is shown.

(D) Fourteen days after transplantation, LRC or non-LRC were isolated and 500–800 cells treated ex vivo for 48 hr with daunorubicin (DAU; 250 nM), mitoxantrone (MITO; 675 nM), amsacrine (AMSA; 18 nM), or etoposide (ETO; 300 nM). Spontaneous cell death in the absence of cytotoxic drugs was 60%; a mean of eight data points from three independent experiments in triplicates or duplicates is shown for DAU and MITO and one experiment in triplicates is shown for AMSA and ETO. Four thousand untreated cells and MRD cells were treated ex vivo for 48 hr with 15 μM ETO, 450 μM MITO, 300 nM VCR, or 500 nM DOX. Cell death was measured by flow cytometry; spontaneous cell death in the absence of cytotoxic drugs was 33%; shown is one experiment in triplicate; mean ± SE; ns, not significant, ***$p < 0.001$ by two-tailed unpaired t test.

(E) Summary of ALL-265 data from Figure 4C (n = 5), S6 (n = 3), and 8D (n = 3); ns, not significant, *$p < 0.05$ and ****$p < 0.0001$ by two-tailed unpaired t test.

Here, we provide a preclinical tool to study dormant human ALL cells in vivo and show that long-term resting cells exist in ALL. This fact was previously unknown, as primary patients' samples allow quantifying non-cycling cells in a snapshot at a given moment, but fail to distinguish between short- and long-term resting cells (Lutz et al., 2013). As monitoring functionally defined cellular subpopulations such as LRC in longitudinal studies is still impossible in patients, our preclinical model enables the gaining of insights into ALL biology that cannot be obtained in patients: here the presence of long-term resting cells in ALL. Beyond its use in preclinical treatment trials, PDX models harbor major potential in basic research and enable unique insights into disease biology.

The emergence of relapse is a complex process involving genetic and non-genetic factors. Early relapse might be caused by a putatively pre-existing clone with additional mutations responsible for drug resistance, especially in adult patients. The genetic stability of most cases of ALL suggests that many relapses may not be mediated by mutational mechanisms. Late relapse might be caused by persisting, dormant tumor cells in the absence of additional mutations, and relapse cells often respond to the identical drugs used to treat the primary disease. LRC represent surrogates for late relapse and relapse in the absence of additional mutations, as often seen in children.

The fact that LRC exist might explain why ALL patients benefit from maintenance therapy, even in prognostically favorable,

(E) All genes differentially expressed (padj < 0.05) between primary samples from five children before onset of treatment to three matched MRD samples 33 days after onset of treatment.

(F) Scatterplot of fold-changes for genes differentially expressed between both LRC versus non-LRC and primary MRD versus primary diagnostic cells, grey area indicates LRC signature (left panel); GSEA comparing the LRC signature with differentially expressed genes between primary MRD and primary diagnostic cells (right panel).

(G) PCA of bulk samples transcriptomes using all shared expressed genes; each symbol indicates a single sample.

See also Figure S7, Tables S7, and S8.

chemo-sensitive ALL subtypes. ALL patients are routinely treated with oral low-dose chemotherapy from end of intensive chemotherapy until, e.g., 2 years after diagnosis, and maintenance therapy improves patients' prognosis (Schrappe et al., 2000). Low-dose maintenance therapy might act by removing LRC-type ALL cells with relapse-inducing potential that remained quiescent over prolonged periods of time and turned on their cell cycle at late time points in the months following intensive chemotherapy.

Tumor cells often display both dormancy and drug resistance. It is unclear whether either dormancy or drug resistance is pivotal in respect to the other, so that dormancy is a consequence of resistance or vice versa (Blatter and Rottenberg, 2015). Our two complementary mouse models show that LRC were defined by their dormant nature and displayed drug resistance, while MRD cells were defined by their ability to survive drug treatment and displayed a dormant phenotype. Thus, both characteristics might be equally sufficient to determine each other and coincide interdependently.

Our study shows that ALL consists of functionally heterogeneous cells regarding proliferation rate and drug resistance, similar to the functional heterogeneity shown in other tumor entities (Kreso et al., 2013). As LRC did not substantially participate in proliferation during growth of leukemia over weeks, in our model LRC existed before onset of therapy and were not developed as a consequence of treatment. As both LRC and non-LRC contain similar amounts of stem cells, but show different sensitivity toward drug treatment in vivo, our data imply that stemness and drug resistance are not directly connected in ALL.

So how does a rare subpopulation acquire the three clinically challenging features dormancy, resistance, and stemness? LRC might represent a cell subpopulation with genuinely different biology harboring distinct intrinsic, constant characteristics, or being an LRC might represent a reversible, temporary, functional phenotype depending on circumstances. In the first case, LRC and non-LRC might be organized in a hierarchical way similar to the known stem cell hierarchy existing in many tumors including AML (Kreso and Dick, 2014). In the second case, ALL cells might mimic the phenotypic reversibility of normal hematopoiesis, where long-term dormant hematopoietic stem cells start cycling in response to stress for a defined period of time and turn back into dormancy later (Trumpp et al., 2010).

Our data favor the second scenario as LRC exhibit their specific characteristics as reversible, temporary, transient functional phenotypes. Re-transplantation experiments showed that formerly dormant LRC started proliferating as soon as they were dissociated from their in vivo environment and transferred into next recipient mice. Upon re-transplantation, LRC converted into non-LRC, while certain non-LRC converted into LRC. Both LRC and non-LRC thus harbored plasticity to switch between slow and rapid proliferation depending on the current context. This fact might explain the area of overlap between LRC and non-LRC detected in single-cell RNA sequencing.

Besides proliferation, drug resistance also proved to be a transient characteristic. Drug-treatment experiments showed that LRC lost their in vivo drug resistance upon ex vivo culture. The discrepancy between drug sensitivity ex vivo and in vivo might at least partly explain the limited predictability of ex vivo drug-screening tests for the outcome of cancer patients (Wilding and Bodmer, 2014). Thus, localization of LRC to the bone marrow niche influences both dormancy and drug resistance.

These insights have translational implications. For diagnostics, as LRC lose their clinically relevant characteristics upon release from their niche, rapid sample processing might be critical for reliable profiling, which represents a challenge in clinical routine (Bacher et al., 2010). Our data at least in part explain the limited power of in vitro assays using, e.g., proliferating cell lines, for studies on MRD cells or primary leukemia cells for drug testing in the absence of feeders. Most importantly for putative treatment strategies, the transient nature of the adverse characteristics of LRC suggests aiming at removing MRD cells from their protective environment to sensitize them toward treatment (Essers et al., 2009; Essers and Trumpp, 2010). The interaction between MRD cells and their bone marrow niche represents a promising target for therapeutic approaches to prevent relapse. Beyond the tumor cell itself, its interaction with the environment represents a suitable therapeutic target. As a caveat, a persistent therapeutic inhibition of the bone marrow niche might be required over prolonged periods of time, as in principle each and every remaining non-LRC ALL cell could convert into a drug-resistant LRC, as soon as it gets access to the protective niche.

At this point, we can only speculate which signals might determine whether an ALL cell behaves like an LRC or a non-LRC. In theory, external as well as internal factors or conditions might be influential; stimuli might be sent or received either stochastically or within a well-regulated process. As our studies were restricted to bone marrow, the bone marrow niche is a likely candidate for a regulatory function and requires investigatory work (Raaijmakers, 2011). Further research is required to address these important questions. Obvious candidates for therapeutic intervention are cell surface molecules expressed on LRC, the inhibition of which might release cells from their environment. Similarly, niche cells could be targeted to aim at reducing environmental support.

Our study shows that ALL growing in vivo contains a rare subpopulation of LRC that exhibits typical challenging adverse characteristics of relapse induction, which proved to be of a reversible nature. Our model might help to develop future anti-leukemia treatment strategies allowing the eradication of the precarious subpopulation of drug-resistant stem cells to prevent relapse and improve the prognosis of patients with ALL.

EXPERIMENTAL PROCEDURE

Ethical Statements

Written informed consent was obtained from all patients and from parents/carers in the cases where the patients were minors. The study was performed in accordance with the ethical standards of the responsible committee on human experimentation (written approval by Ethikkommission des Klinikums der Ludwig-Maximilians-Universität München, Ethikkommission@med.unimuenchen.de, April 15, 2008, number 068-08) and with the Helsinki Declaration of 1975, as revised in 2000.

All animal trials were performed in accordance with the current ethical standards of the official committee on animal experimentation under the written approvals by Regierung von Oberbayern, poststelle@reg-ob.bayern.de, May 10, 2007 number 55.2-1-54-2531-2-07 and August 8, 2010 number 55.2-1-54-2531-95-10.

Enriching and Quantifying PDX and LRC from Mouse Bone Marrow

PDX ALL cells were genetically engineered as described using lentiviruses (Terziyska et al., 2012; Vick et al., 2015) to express the transgenes' truncated NGFR, a red fluorochrome, and luciferase; cells were stained with BrdU and/or CFSE before retransplantation of fresh cells into mice.

For determining the fraction of dormant PDX ALL cells, mouse bone marrow was harvested from numerous bones and enriched for human PDX ALL cells using NGFR for MACS and the red fluorochrome for flow cytometry cell sorting. LRC were discriminated from non-LRC using CFSE staining as shown in Figure 1D. CFSE mean fluorescence intensity (MFI) was measured at day 3 after injection, when bleaching had ceased, and defined cells before the onset of proliferation ("0 divisions"). Day 3 CFSE MFI was divided by factor 2 to calculate CFSE bisections mimicking cell divisions. Seven CFSE MFI bisections or more were defined as entire loss of the CFSE signal characterizing non-LRC. The LRC gate was set to include all cells harboring high CFSE signal of below three bisections of the maximum CFSE MFI (Schillert et al., 2013) (Figure 1D).

PDX Single-Cell RNA-Seq Library Construction

Single cells were isolated at 4°C and processed on the Fluidigm C1 platform. In brief, 500 cells were loaded on the 10–17 μm mRNA-seq IFC (Fluidigm) with External RNA Controls Consortium spike-in controls. Cell lysis, reverse transcription, and pre-amplification of cDNA was done on-chip using the SMARTer Ultra Low RNA Kit for C1 (Clontech). Harvested cDNA libraries of the samples (2.5 μL) were used as input for tagmentation with the Nextera XT Sample Preparation Kit (Illumina) at half the volume of Illumina's protocol. Barcoding PCR was performed for 12 cycles. Equal amounts of libraries were pooled.

RNA-Seq

Single-cell Smart-seq and bulk Smart-seq2 libraries were sequenced at 1 × 50 bases on an Illumina HiSeq1500. SCRB-seq and UMI-seq libraries were sequenced paired-end with 16 cycles on the first read to decode sample barcodes and unique molecular identifiers and 50 cycles on the second read into the cDNA fragment.

ACCESSION NUMBERS

RNA-seq data reported in this paper have been deposited in the NCBI's GEO database and are accessible through the GEO Series accession number GEO: GSE83142.

SUPPLEMENTAL INFORMATION

Supplemental Information includes Supplemental Experimental Procedures, eight figures, and eight tables and can be found with this article online at http://dx.doi.org/10.1016/j.ccell.2016.11.002.

AUTHOR CONTRIBUTIONS

S.E. and E.Z.Ö. planned, performed, and analyzed the experiments and designed the data presentation; C.Z., E.Z.Ö., S.P., and W.E. generated and analyzed RNA-seq data, W.E. participated in writing the manuscript; S.T. and C.C.A. established the mouse model, first detected LRC, and started their characterization; M.G. started establishing the MRD PDX model; A.S. guided the work of E.Z.Ö.; M.D. performed enrichment of pediatric MRD cells provided by R.P.G.; C.L., V.A.T., and T.E. performed enrichment of adult StemB cells provided by H.P.; H.P.H. and K.So. performed immunohistochemistry of primary bone marrow biopsies; K.Sp. and W.H. provided primary adult samples of Figure S2; B.P., S.K., M.H., and B.K. performed and analyzed gene expression array data; J.H. performed the mathematical analysis of Figure S1E; O.G. participated in designing the experiments, guiding the study, and writing the manuscript; I.J. initiated and guided the study and wrote the manuscript.

ACKNOWLEDGMENTS

We thank Jean Pierre Bourquin and Beat Bornhäuser for providing engrafted sample ALL-265 and Cornelia Eckert and the I-BFM study group for providing the clinical data on sample ALL-265. We thank Markus G. Manz for helpful scientific discussions, Hitoshi Takizawa for help in establishing CFSE staining, Susanne Suhendra for sorting pediatric MRD cells, Volker Eckstein, Panagiotis Gitsioudis, and Linda Manta for their help in sorting stemB cells, Michael Hagemann and his team for excellent animal care, Lothar Strobl for help in array analysis, Kai Höfig for help with qPCR, Andreas Sendelhofert for establishing immunohistochemistry, Annette Frank and Volker Groiβ for help in mice experiments, Liliana Mura and Fabian Klein for technical support, and Michela Carlet, Cornelia Finkenzeller, and Binje Vick for helpful discussions. The work was supported by ERC Consolidator Grant 681524; Deutsche José Carreras Leukämie-Stiftung (R 10/26); the Collaborative Research Centers 684 Molecular Mechanisms of Normal and Malignant Hematopoiesis, project A22 and 1243 Genetic and Epigenetic Evolution of Hematopoetic Neoplasms, project A05; the German Consortium for Translational Cancer Research (DKTK); Bettina Bräu-Stiftung; and Dr. Helmut Legerlotz Stiftung (all to I.J.); Collaborative Research Center 1243, project A14, to W.E.; project 07 to K.S., and project 08 to W.H.; the German Federal Ministry of Education and Research (BMBF) within the Virtual Liver Project (grant no. 0315766) to J.H.; and the German Research Foundation grants SPP1395/InKoMBio Busch 900/6-1 to B.K. and DFG GI-540-3/1 to O.G.

Received: August 19, 2015
Revised: June 11, 2016
Accepted: October 31, 2016
Published: December 1, 2016

REFERENCES

Bacher, U., Kohlmann, A., and Haferlach, T. (2010). Gene expression profiling for diagnosis and therapy in acute leukaemia and other haematologic malignancies. Cancer Treat Rev. *36*, 637–646.

Blatter, S., and Rottenberg, S. (2015). Minimal residual disease in cancer therapy – small things make all the difference. Drug Resist Updat *21-22*, 1–10.

Bonnet, D., and Dick, J.E. (1997). Human acute myeloid leukemia is organized as a hierarchy that originates from a primitive hematopoietic cell. Nat. Med. *3*, 730–737.

Castro Alves, C., Terziyska, N., Grunert, M., Gündisch, S., Graubner, U., Quintanilla-Martinez, L., and Jeremias, I. (2012). Leukemia-initiating cells of patient-derived acute lymphoblastic leukemia xenografts are sensitive toward TRAIL. Blood *119*, 4224–4227.

Clevers, H. (2011). The cancer stem cell: premises, promises and challenges. Nat. Med. *17*, 313–319.

Eppert, K., Takenaka, K., Lechman, E.R., Waldron, L., Nilsson, B., van Galen, P., Metzeler, K.H., Poeppl, A., Ling, V., Beyene, J., et al. (2011). Stem cell gene expression programs influence clinical outcome in human leukemia. Nat. Med. *17*, 1086–1093.

Essers, M.A.G., and Trumpp, A. (2010). Targeting leukemic stem cells by breaking their dormancy. Mol. Oncol. *4*, 443–450.

Essers, M.A., Offner, S., Blanco-Bose, W.E., Waibler, Z., Kalinke, U., Duchosal, M.A., and Trumpp, A. (2009). IFNalpha activates dormant haematopoietic stem cells in vivo. Nature *458*, 904–908.

Fehse, B., Uhde, A., Fehse, N., Eckert, H.G., Clausen, J., Ruger, R., Koch, S., Ostertag, W., Zander, A.R., and Stockschlader, M. (1997). Selective

immunoaffinity-based enrichment of CD34+ cells transduced with retroviral vectors containing an intracytoplasmatically truncated version of the human low-affinity nerve growth factor receptor (deltaLNGFR) gene. Hum. Gene Ther. *8*, 1815–1824.

Gao, H., Korn, J.M., Ferretti, S., Monahan, J.E., Wang, Y., Singh, M., Zhang, C., Schnell, C., Yang, G., Zhang, Y., et al. (2015). High-throughput screening using patient-derived tumor xenografts to predict clinical trial drug response. Nat. Med. *21*, 1318–1325.

Georgantas, R.W., 3rd, Tanadve, V., Malehorn, M., Heimfeld, S., Chen, C., Carr, L., Martinez-Murillo, F., Riggins, G., Kowalski, J., and Civin, C.I. (2004). Microarray and serial analysis of gene expression analyses identify known and novel transcripts overexpressed in hematopoietic stem cells. Cancer Res. *64*, 4434–4441.

Gokbuget, N., Stanze, D., Beck, J., Diedrich, H., Horst, H.A., Huttmann, A., Kobbe, G., Kreuzer, K.A., Leimer, L., Reichle, A., et al. (2012). Outcome of relapsed adult lymphoblastic leukemia depends on response to salvage chemotherapy, prognostic factors, and performance of stem cell transplantation. Blood *120*, 2032–2041.

Graham, S.M., Vass, J.K., Holyoake, T.L., and Graham, G.J. (2007). Transcriptional analysis of quiescent and proliferating CD34+ human hemopoietic cells from normal and chronic myeloid leukemia sources. Stem Cells *25*, 3111–3120.

Hong, D., Gupta, R., Ancliff, P., Atzberger, A., Brown, J., Soneji, S., Green, J., Colman, S., Piacibello, W., Buckle, V., et al. (2008). Initiating and cancer-propagating cells in TEL-AML1-associated childhood leukemia. Science *319*, 336–339.

Inaba, H., Greaves, M., and Mullighan, C.G. (2013). Acute lymphoblastic leukaemia. Lancet *381*, 1943–1955.

Kamel-Reid, S., Letarte, M., Sirard, C., Doedens, M., Grunberger, T., Fulop, G., Freedman, M., Phillips, R., and Dick, J. (1989). A model of human acute lymphoblastic leukemia in immune-deficient SCID mice. Science *246*, 1597–1600.

Kang, H., Chen, I.M., Wilson, C.S., Bedrick, E.J., Harvey, R.C., Atlas, S.R., Devidas, M., Mullighan, C.G., Wang, X., Murphy, M., et al. (2010). Gene expression classifiers for relapse-free survival and minimal residual disease improve risk classification and outcome prediction in pediatric B-precursor acute lymphoblastic leukemia. Blood *115*, 1394–1405.

Kong, Y., Yoshida, S., Saito, Y., Doi, T., Nagatoshi, Y., Fukata, M., Saito, N., Yang, S.M., Iwamoto, C., Okamura, J., et al. (2008). CD34+CD38+CD19+ as well as CD34+CD38-CD19+ cells are leukemia-initiating cells with self-renewal capacity in human B-precursor ALL. Leukemia *22*, 1207–1213.

Kreso, A., and Dick, J.E. (2014). Evolution of the cancer stem cell model. Cell Stem Cell *14*, 275–291.

Kreso, A., O'Brien, C.A., van Galen, P., Gan, O.I., Notta, F., Brown, A.M., Ng, K., Ma, J., Wienholds, E., Dunant, C., et al. (2013). Variable clonal repopulation dynamics influence chemotherapy response in colorectal cancer. Science *339*, 543–548.

Kunz, J.B., Rausch, T., Bandapalli, O.R., Eilers, J., Pechanska, P., Schuessele, S., Assenov, Y., Stutz, A.M., Kirschner-Schwabe, R., Hof, J., et al. (2015). Pediatric T-cell lymphoblastic leukemia evolves into relapse by clonal selection, acquisition of mutations and promoter hypomethylation. Haematologica *100*, 1442–1450.

le Viseur, C., Hotfilder, M., Bomken, S., Wilson, K., Röttgers, S., Schrauder, A., Rosemann, A., Irving, J., Stam, R.W., Shultz, L.D., et al. (2008). In childhood acute lymphoblastic leukemia, blasts at different stages of immunophenotypic maturation have stem cell properties. Cancer Cell *14*, 47–58.

Liberzon, A., Birger, C., Thorvaldsdottir, H., Ghandi, M., Mesirov, J.P., and Tamayo, P. (2015). The molecular signatures database (MSigDB) hallmark gene set collection. Cell Syst *1*, 417–425.

Lutz, C., Woll, P.S., Hall, G., Castor, A., Dreau, H., Cazzaniga, G., Zuna, J., Jensen, C., Clark, S.A., Biondi, A., et al. (2013). Quiescent leukaemic cells account for minimal residual disease in childhood lymphoblastic leukaemia. Leukemia *27*, 1204–1207.

Morrison, S.J., and Spradling, A.C. (2008). Stem cells and niches: mechanisms that promote stem cell maintenance throughout life. Cell *132*, 598–611.

Raaijmakers, M.H. (2011). Niche contributions to oncogenesis: emerging concepts and implications for the hematopoietic system. Haematologica *96*, 1041–1048.

Ravandi, F., Jorgensen, J.L., O'Brien, S.M., Jabbour, E., Thomas, D.A., Borthakur, G., Garris, R., Huang, X., Garcia-Manero, G., Burger, J.A., et al. (2016). Minimal residual disease assessed by multi-parameter flow cytometry is highly prognostic in adult patients with acute lymphoblastic leukaemia. Br. J. Haematol. *172*, 392–400.

Rehe, K., Wilson, K., Bomken, S., Williamson, D., Irving, J., den Boer, M.L., Stanulla, M., Schrappe, M., Hall, A.G., et al. (2013). Acute B lymphoblastic leukaemia-propagating cells are present at high frequency in diverse lymphoblast populations. EMBO Mol. Med. *5*, 38–51.

Saito, Y., Kitamura, H., Hijikata, A., Tomizawa-Murasawa, M., Tanaka, S., Takagi, S., Uchida, N., Suzuki, N., Sone, A., Najima, Y., et al. (2010). Identification of therapeutic targets for quiescent, chemotherapy-resistant human leukemia stem cells. Sci. Transl Med. *2*, 17ra19.

Schillert, A., Trumpp, A., and Sprick, M.R. (2013). Label retaining cells in cancer – the dormant root of evil? Cancer Lett. *341*, 73–79.

Schmitz, M., Breithaupt, P., Scheidegger, N., Cario, G., Bonapace, L., Meissner, B., Mirkowska, P., Tchinda, J., Niggli, F.K., Stanulla, M., et al. (2011). Xenografts of highly resistant leukemia recapitulate the clonal composition of the leukemogenic compartment. Blood *118*, 1854–1864.

Schrappe, M. (2014). Detection and management of minimal residual disease in acute lymphoblastic leukemia. Hematology *2014*, 244–249.

Schrappe, M., Reiter, A., Zimmermann, M., Harbott, J., Ludwig, W.D., Henze, G., Gadner, H., Odenwald, E., and Riehm, H. (2000). Long-term results of four consecutive trials in childhood ALL performed by the ALL-BFM study group from 1981 to 1995. Berlin-Frankfurt-Munster. Leukemia *14*, 2205–2222.

Takizawa, H., Regoes, R.R., Boddupalli, C.S., Bonhoeffer, S., and Manz, M.G. (2011). Dynamic variation in cycling of hematopoietic stem cells in steady state and inflammation. J. Exp. Med. *208*, 273–284.

Terziyska, N., Alves, C.C., Groiss, V., Schneider, K., Farkasova, K., Ogris, M., Wagner, E., Ehrhardt, H., Brentjens, R.J., zur Stadt, U., et al. (2012). In vivo imaging enables high resolution preclinical trials on patients' leukemia cells growing in mice. PLoS One *7*, e52798.

Tesfai, Y., Ford, J., Carter, K.W., Firth, M.J., O'Leary, R.A., Gottardo, N.G., Cole, C., and Kees, U.R. (2012). Interactions between acute lymphoblastic leukemia and bone marrow stromal cells influence response to therapy. Leuk. Res. *36*, 299–306.

Townsend, E.C., Murakami, M.A., Christodoulou, A., Christie, A.L., Koster, J., DeSouza, T.A., Morgan, E.A., Kallgren, S.P., Liu, H., Wu, S.C., et al. (2016). The public repository of xenografts enables discovery and randomized phase II-like trials in mice. Cancer Cell *29*, 574–586.

Trumpp, A., Essers, M., and Wilson, A. (2010). Awakening dormant haematopoietic stem cells. Nat. Rev. Immunol. *10*, 201–209.

Vick, B., Rothenberg, M., Sandhofer, N., Carlet, M., Finkenzeller, C., Krupka, C., Grunert, M., Trumpp, A., Corbacioglu, S., Ebinger, M., et al. (2015). An advanced preclinical mouse model for acute myeloid leukemia using patients' cells of various genetic subgroups and in vivo bioluminescence imaging. PLoS One *10*, e0120925.

Visvader, J.E., and Lindeman, G.J. (2008). Cancer stem cells in solid tumours: accumulating evidence and unresolved questions. Nat. Rev. Cancer *8*, 755–768.

Wilding, J.L., and Bodmer, W.F. (2014). Cancer cell lines for drug discovery and development. Cancer Res. *74*, 2377–2384.

Zhou, B.-B.S., Zhang, H., Damelin, M., Geles, K.G., Grindley, J.C., and Dirks, P.B. (2009). Tumour-initiating cells: challenges and opportunities for anticancer drug discovery. Nat. Rev. Drug Discov. *8*, 806–823.

Cancer Cell, Volume *30*

Supplemental Information

Characterization of Rare, Dormant, and Therapy-Resistant Cells in Acute Lymphoblastic Leukemia

Sarah Ebinger, Erbey Ziya Özdemir, Christoph Ziegenhain, Sebastian Tiedt, Catarina Castro Alves, Michaela Grunert, Michael Dworzak, Christoph Lutz, Virginia A. Turati, Tariq Enver, Hans-Peter Horny, Karl Sotlar, Swati Parekh, Karsten Spiekermann, Wolfgang Hiddemann, Aloys Schepers, Bernhard Polzer, Stefan Kirsch, Martin Hoffmann, Bettina Knapp, Jan Hasenauer, Heike Pfeifer, Renate Panzer-Grümayer, Wolfgang Enard, Olivier Gires, and Irmela Jeremias

Supplemental Data

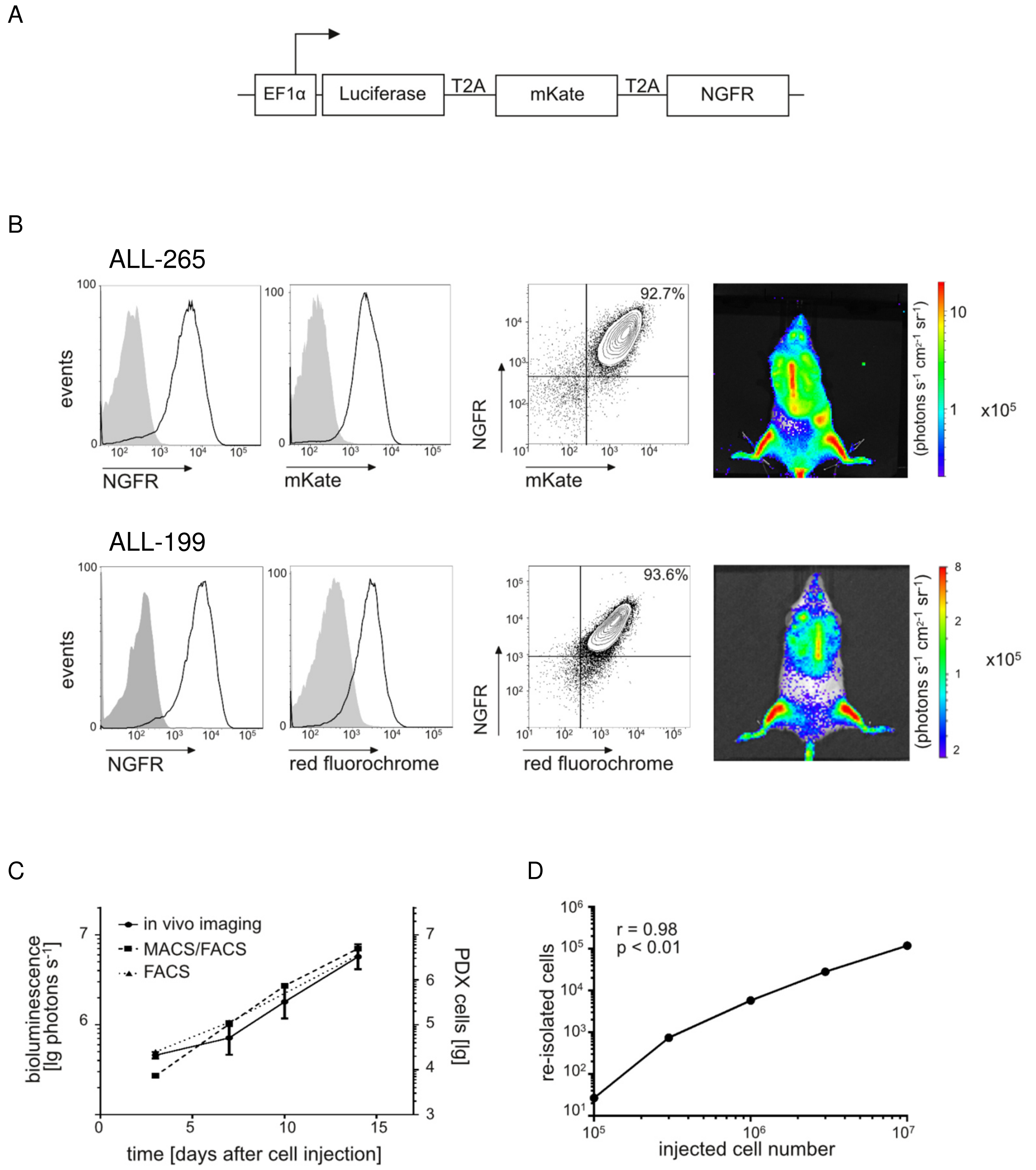

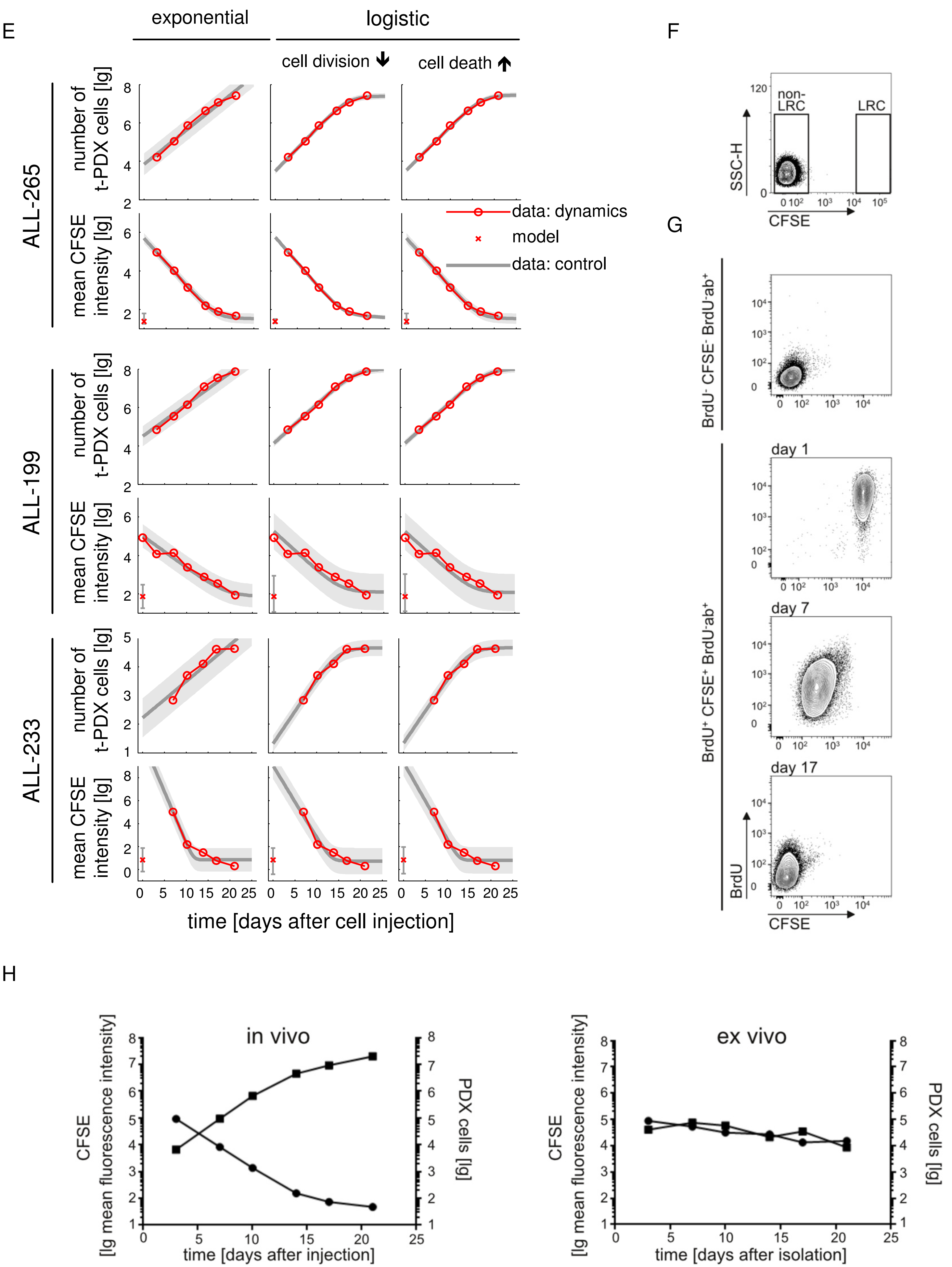
E
exponential
logistic
cell division
cell death
ALL-265
ALL-199
ALL-233
number of t-PDX cells [lg]
mean CFSE intensity [lg]
data: dynamics
model
data: control
time [days after cell injection]
F
non-LRC
LRC
SSC-H
CFSE
G
BrdU⁻ CFSE⁻ BrdU-ab⁺
BrdU⁺ CFSE⁺ BrdU-ab⁺
day 1
day 7
day 17
BrdU
CFSE
H
in vivo
ex vivo
CFSE [lg mean fluorescence intensity]
PDX cells [lg]
time [days after injection]
time [days after isolation]

Figure S1, related to Figure 1.
CFSE staining allows reliable monitoring of PDX ALL growth in mice.

(A) Lentiviral construct for equimolar expression of 3 transgenes; arrow indicates start of transcription; EF1α = elongation factor 1-alpha promoter; mKate = red fluorescent protein cloned from sea anemone Entacmaea quadricolor; NGFR = human low affinity nerve growth factor receptor lacking the intracellular signaling domain.

(B) Quality controls on enriched transgenic PDX ALL-265 or ALL-199 cells by flow cytometry or bioluminescence in vivo imaging.

(C) 10^7 ALL-265 cells were injected into groups of mice and one mouse was sacrificed at each time point. In vivo imaging was performed directly before cell harvesting and quantifying PDX cells by flow cytometry with and without prior MACS selection; mean of each group +/- standard error.

(D) Different cell numbers of ALL-199 cells were injected in mice at and re-isolated after 3 days; each dot indicates data from one animal.

(E) The measured numbers of PDX cells and the measured mean fluorescence intensities of CFSE were fitted with three mechanistic ordinary differential equation models assuming: exponential growth; logistic growth caused by a decreased rate of cell division at higher cell densities; and logistic growth caused by a increased rate of cell death at higher cell densities. The measured data (red circles and crosses), the best fit (gray line) and the noise related uncertainty intervals (gray shaded area) are depicted.

(F) No cells devoid of CFSE labeling are found in the LRC gate; flow cytometry analysis at day 0 of unlabeled ALL-265 PDX cells.

(G) Controls for BrdU and CFSE stainings; BrdU indicates feeding of mice and cells with BrdU; BrdU-ab indicates that cells were stained with the anti-BrdU antibody; “+” and “-” indicate that the procedures were performed or not, respectively.

(H) To compere behavior of PDX cells in vivo and ex vivo, 10^7 ALL-265 cells were injected into groups of mice and one mouse was sacrificed at each time point to isolate PDX cells (left panel); 10^7 fresh CFSE labeled ALL-265 PDX cells per ml were cultured on MS-5 feeder cells ex vivo (right panel).

Table S1, related to Figure 1.
Clinical data of patients donating diagnostic ALL cells for xenotransplantation and sample characteristics.

sample	type of ALL	disease stage*	age* [years]	sex	cytogenetics	passaging time§ [days]
**ALL-199**	BCP-ALL pediatric	2nd relapse	8	F	somatic trisomy 21; leukemic homozygous 9p deletion	42
**ALL-233**	BCP-ALL pediatric	initial diagnosis	<1	M	t(2;15)(p13;q15)	76
**ALL-265**	BCP-ALL pediatric	1st relapse	5	F	hyperploidy with additional 6, 13, 14, 17, 18, 21, X chromosome	43
**ALL-435**	BCP-ALL pediatric	initial diagnosis	<1	M	MLL-ENL, t(11;19)	40
**ALL-50**	BCP-ALL pediatric	initial diagnosis	7	F	BCR/ABL positive	45
**ALL-177**	BCP-ALL pediatric	initial diagnosis	8	F	TEL/AML1 positive	130
**ALL-230**	T-ALL pediatric	initial diagnosis	4	M	t(11;14)(p32;q11); rearrangement of TAL1-gene with the T-cell receptor locus	35
**ALL-256**	BCP-ALL adult	initial diagnosis	41	F	trisomy 8; BCR/ABL positive t(9;22)(q34;q11)	75
**ALL-363**	BCP-ALL adult	initial diagnosis	65	M	BCR-ABL positive t(9;22)(q34,q11)	60

BCP=B-cell precursor; *when the primary ALL sample was obtained; §time of passaging through mice refers to the time from injection of the sample until mice had to be sacrificed due to end stage leukemia

Table S2, related to Figure 1.
Two step procedure allows enrichment of minute numbers of PDX cells from mouse bone marrow.

mixed*		recovered		
**mouse bone marrow cells**	**PDX cells**	**number of cells**	**% recovery**	**enrichment Factor§**
$1x10^8$	1,250	1,234	99	81,000
$1x10^8$	12,500	10,262	82	9,700
$1x10^8$	37,500	34,679	92	2,666

*$1x10^8$ mouse bone marrow cells were mixed with different numbers of ALL-265 PDX cells expressing NGFR and mKate; MACS-based enrichment targeting NGFR-expressing cells was followed by flow cytometry-based enrichment targeting mKate-expressing cells; §enrichment factor was calculated as ratio from “number of mouse bone marrow cells” and “recovered number of cells”

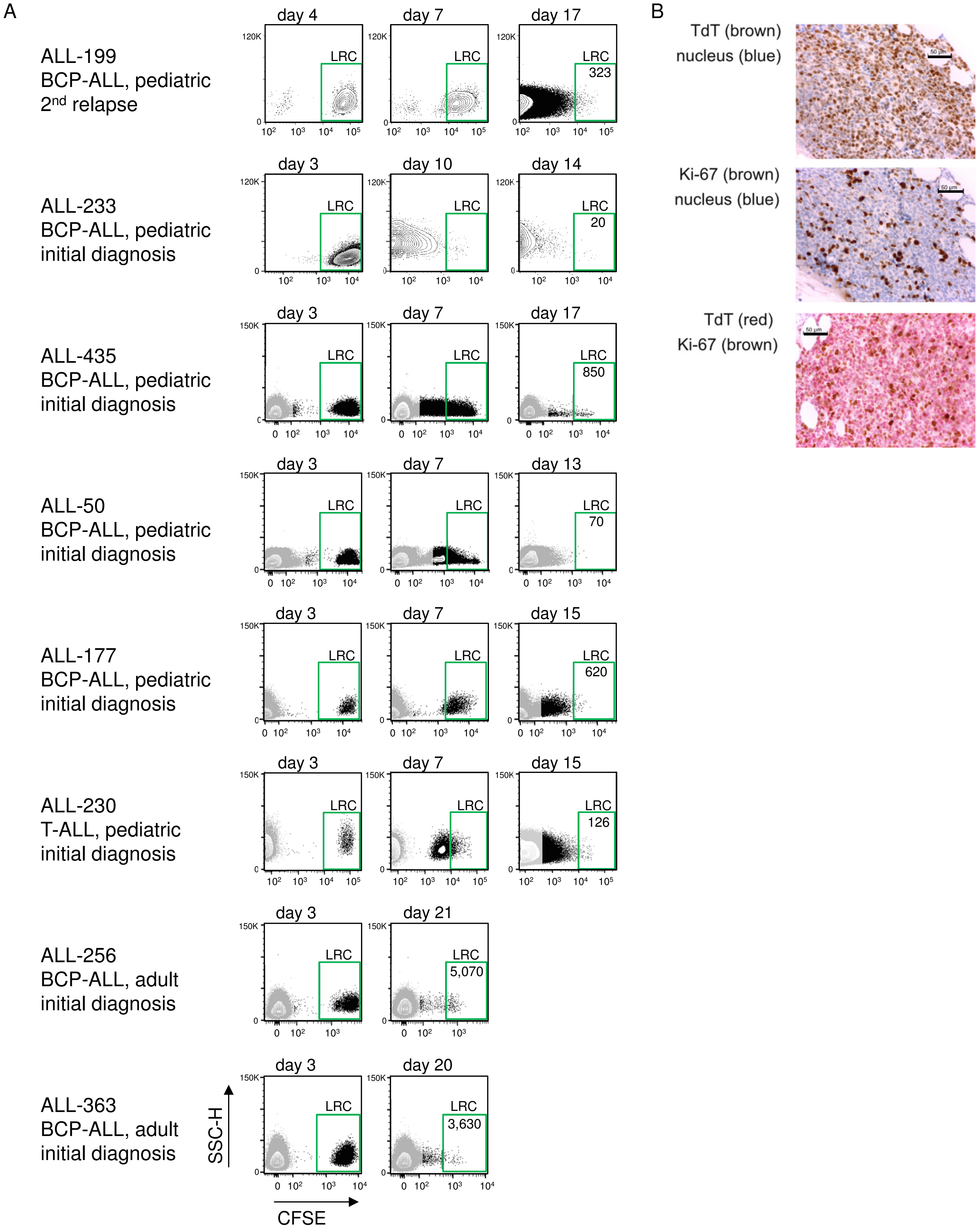
A
ALL-199
BCP-ALL, pediatric
2nd relapse
ALL-233
BCP-ALL, pediatric
initial diagnosis
ALL-435
BCP-ALL, pediatric
initial diagnosis
ALL-50
BCP-ALL, pediatric
initial diagnosis
ALL-177
BCP-ALL, pediatric
initial diagnosis
ALL-230
T-ALL, pediatric
initial diagnosis
ALL-256
BCP-ALL, adult
initial diagnosis
ALL-363
BCP-ALL, adult
initial diagnosis
day 4
day 7
day 17
LRC
323
day 3
day 10
day 14
20
day 13
850
70
day 15
620
126
day 21
5,070
day 20
3,630
SSC-H
CFSE
B
TdT (brown)
nucleus (blue)
Ki-67 (brown)
nucleus (blue)
TdT (red)
Ki-67 (brown)

Figure S2, related to Figure 2.
A rare, long-term dormant subpopulation exists in ALL PDX cells growing in mice.

(A) 10^7 CFSE labeled ALL-199 cells/mouse were injected into 3 mice and PDX cells were enriched from bone marrow of 1 mouse at each time point using MACS sorting targeting NGFR and FACS sorting targeting mKate; LRC and non-LRC were quantified by flow cytometry. One representative out of at least 10 independent experiments is shown. All further PDX samples did not express transgenes. Here, 10% of the entire bone marrow isolate was analyzed without a prior MACS enrichment step. Unstained cells represent mouse bone marrow cells and non-LRC. Day = number of days after injection of CFSE-labeled cells.
(B) Immunohistochemistry was performed using TdT to visualize all ALL blasts and Ki-67 to visualize proliferating cells in the diagnostic BM biopsy from one 69 years old female patient with BCR/ABL positive normal karyotype ALL; double staining (lowest panel) indicates frequent dormant ALL blasts as TdT positive, Ki-67 negative cells. Hemalum staining was used for nuclei; scale bar represents 50 µm.

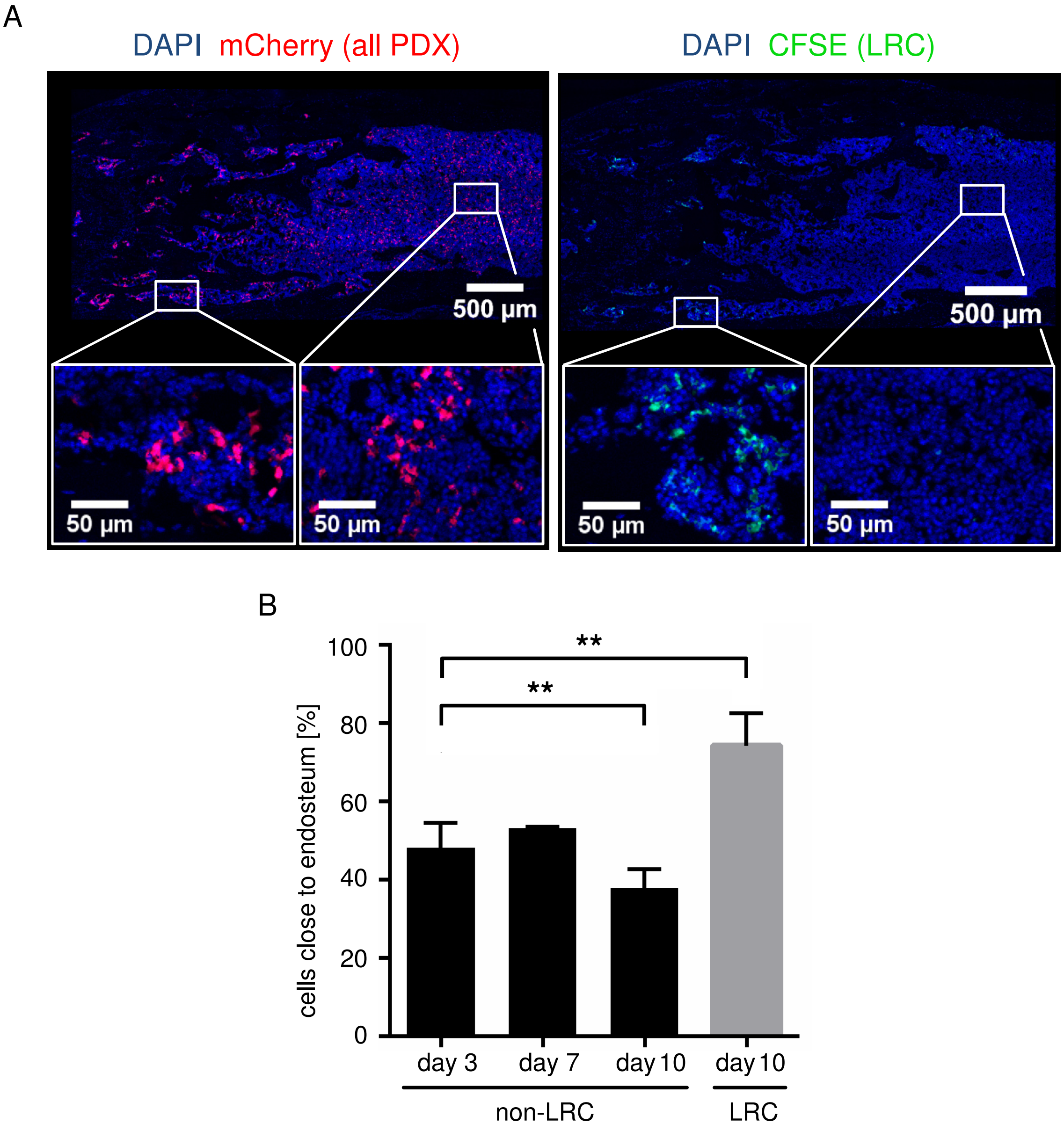

Figure S3, related to Figure 3.
LRC localize to the endosteum in ALL-199.

(A) Immunohistochemistry of consecutive murine bone marrow femur sections 10 days after injection of CFSE-stained PDX ALL-199 cells; mCherry (red) indicates all PDX cells, CFSE (green) indicates LRC.
(B) Kinetic for ALL-199; mean +/- standard error; * $p<0.05$; ** $p<0.01$ by two-tailed unpaired t-test.

Table S3, related to Figure 3.
LRC and non-LRC harbor similar numbers of leukemia initiating cells (LIC).

sample	number of cells injected per mouse*		time [days after injection] 20	28	41	48	62	75
ALL-265	LRC	333	0/2	0/2	0/2	0/2	2/2	2/2
		100	0/5	0/5	0/5	0/5	2/5	3/5
		10						8/19
	LIC frequency		1/40 (CI = 95%; lower = 1/84, upper = 1/19)					
	non-LRC	3333	0/3	1/3	3/3	3/3	3/3	3/3
		1000	0/5	0/5	2/5	4/5	5/5	5/5
		333	0/5	0/5	0/5	0/5	4/5	4/5
		100	0/5	0/5	0/5	0/5	2/5	2/5
		10						8/20
	LIC frequency		1/85 (CI = 95%; lower = 1/179, upper = 1/40)					
	number of cells injected per mouse		time [days after injection] 35	42	49	56	69	77
ALL-199	LRC	333	0/3	0/3	0/3	0/3	2/3	3/3
		100	n.d.	0/4	0/4	0/4	1/4	3/4
	LIC frequency		1/69 (CI = 95%; lower = 1/209, upper = 1/23)					
	non-LRC	1000	1/5	3/5	5/5	5/5	5/5	
		333	0/3	2/3	2/3	3/3	3/3	3/3
		100	0/4	1/4	1/4	3/4	4/4	4/4
	LIC frequency		1/100 or higher					

*LRC and non-LRC obtained 14 days after injection of CFSE labeled ALL-265 or ALL-199 cells were transplanted into secondary recipient mice in limiting dilutions at numbers indicated; bioluminescence in vivo imaging was performed repetitively at the indicated time points to determine engraftment; LIC frequency was calculated using the ELDA software; CI = confidence interval

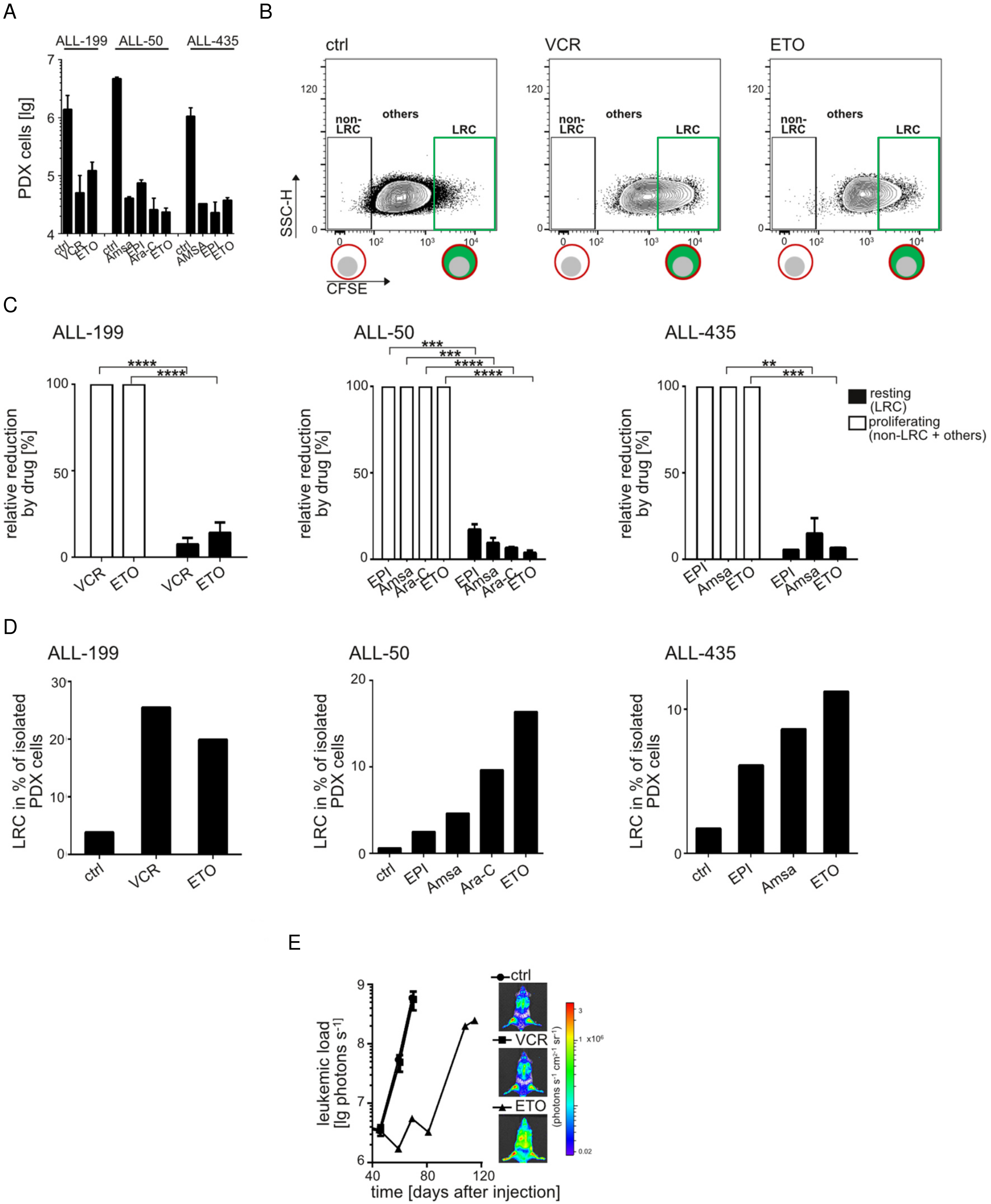
A
ALL-199
ALL-50
ALL-435
PDX cells [lg]
ctrl
VCR
ETO
Amsa
EPI
Ara-C
AMSA
B
ctrl
VCR
ETO
non-LRC
others
LRC
SSC-H
CFSE
C
ALL-199
ALL-50
ALL-435
relative reduction by drug [%]
resting (LRC)
proliferating (non-LRC + others)
****
***
**
D
ALL-199
ALL-50
ALL-435
LRC in % of isolated PDX cells
E
leukemic load [lg photons s-1]
time [days after injection]
ctrl
VCR
ETO
(photons s-1 cm-2 sr-1)
x10^6

Figure S4, related to Figure 4.
LRC survive systemic drug treatment in vivo.

Mice were injected with 10^7 CFSE-labeled ALL PDX cells/mouse, were treated on day 7 and sacrificed on day 10; LRC and non-LRC were analyzed and re-transplanted into 1-2 secondary recipient mice at 2,000-5,000 LRC per mouse.

(A) Numbers of PDX cells isolated from mice with and without prior systemic drug treatment; mean of each group (n=8-11) +/- standard error.
(B) For ALL-199, a second relapse, 11 mice were treated with a single application of vincristine (VCR, 1.5 mg/kg i.v.), 8 mice were treated with a single application of etoposide (ETO, 75 mg/kg i.p.) and 8 control mice received buffer; shown are original data of representative mice.
(C) Quantification in all mice per group depicted as mean of relative drug effects on LRC compared to non-LRC (100%) +/- standard error. For ALL-50, a sample obtained at initial diagnosis, drugs were applied daily over 3 days and 2 mice were treated with cytarabine (AraC, 150 mg/kg i.p.), 2 mice with ETO (33 mg/kg i.p.), 2 mice with amsacrine (Amsa, 25 mg/kg i.p.) and 2 mice with epirubicine (EPI, 25 mg/kg i.p., single application). For ALL-435, another sample obtained at initial diagnosis, drugs were applied daily over 3 days and 2 mice were treated with ETO (33 mg/kg, i.p.), 2 mice with Amsa (25 mg/kg i.p.) and one mouse with EPI (25 mg/kg i.p., single application). ** p< 0.01, *** p<0.001, **** p<0.0001 by two-tailed unpaired t-test.
(D) Mean relative proportion of LRC in total PDX cells with and without treatment.
(E) To study their stem cell potential, LRC of ALL-199 LRC were isolated after treatment, re-transplanted and growth monitored by in vivo imaging mean of each group (n=1-2) +/- standard error. Imaging pictures from dpi 60 (ctrl, VCR) and dpi 108 (ETO).

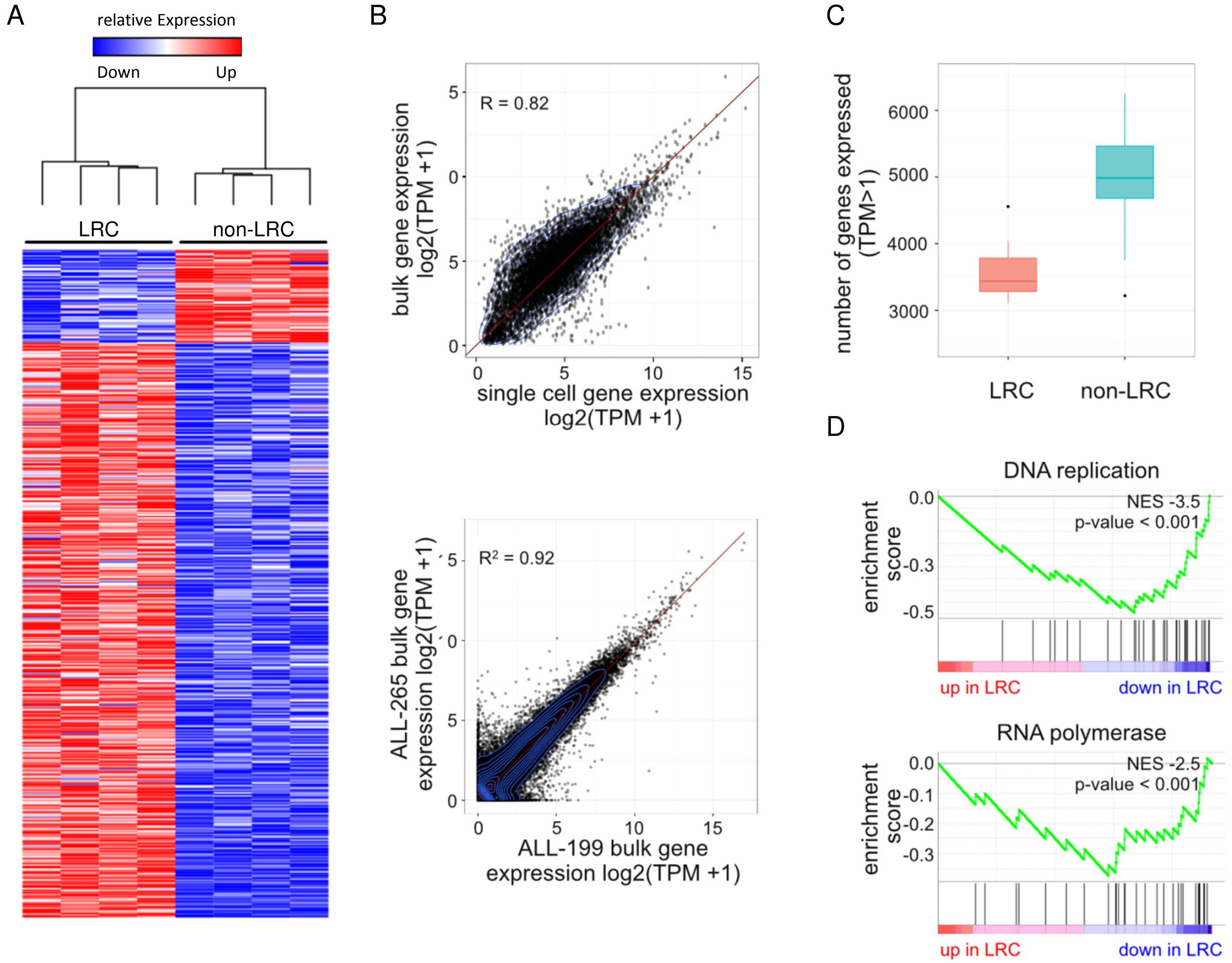

Figure S5, related to Figure 5.
Expression profile of LRC shows distinct changes to non-LRC.

15 days after transplantation of CFSE labeled PDX cells, LRC and non-LRC were subjected to RNA sequencing. For ALL-265, high quality single cell mRNA seq profiles were obtained from 15 LRC and 35 non-LRC cells. To combine single-cell and bulk RNA-seq data, median count data of single-cell experiments were summarized as a single expression profile for each LRC and non-LRC.

(A) Hierarchical clustering and gene expression heatmap across the 500 most differentially expressed genes comparing LRC and non-LRC in ALL-199 ($p < 0.01$).
(B) Comparison of Transcript Per Million (TPM) expression values between bulk versus single-cell ALL-265 (upper) and ALL-265 versus ALL-199 (lower).
(C) Quantification of expressed genes per cell (TPM > 1) in LRC versus non-LRC according to single-cell RNA-seq of ALL-265; shown is the median with upper/lower quartile and maximum/minimum, outliers are shown as dots.
(D) Gene set enrichment analysis for indicated KEGG pathways and the genes differentially regulated in LRC versus non-LRC.

Table S4, related to Figure 5.
List of 500 most differentially expressed genes between LRC and non-LRC in single cell RNA sequencing of ALL-265

Provided as an Excel file.

Table S5, related to Figure 5. Integrated LRC signature.

Provided as an Excel file.

Table S6, related to Figure 5.
KEGG pathways enriched with LRC versus non-LRC differentially expressed genes in combined analysis

Provided as an Excel file.

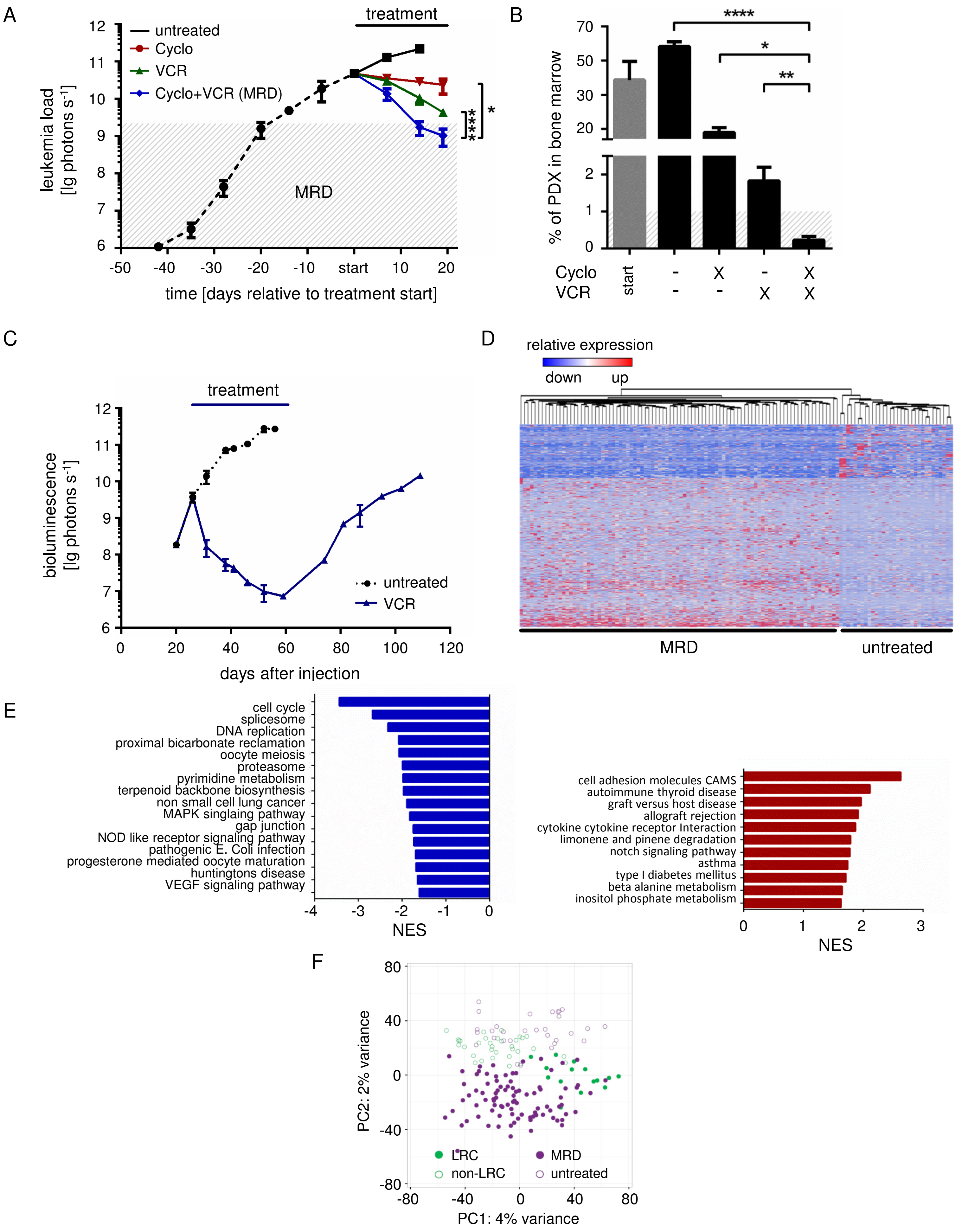

A
treatment
untreated
Cyclo
VCR
Cyclo+VCR (MRD)
leukemia load [lg photons s-1]
MRD
time [days relative to treatment start]
B
% of PDX in bone marrow
****
*
**
Cyclo
VCR
start
C
treatment
bioluminescence [lg photons s-1]
untreated
VCR
days after injection
D
relative expression
down
up
MRD
untreated
E
cell cycle
splicesome
DNA replication
proximal bicarbonate reclamation
oocyte meiosis
proteasome
pyrimidine metabolism
terpenoid backbone biosynthesis
non small cell lung cancer
MAPK singlaing pathway
gap junction
NOD like receptor signaling pathway
pathogenic E. Coli infection
progesterone mediated oocyte maturation
huntingtons disease
VEGF signaling pathway
NES
cell adhesion molecules CAMS
autoimmune thyroid disease
graft versus host disease
allograft rejection
cytokine cytokine receptor Interaction
limonene and pinene degradation
notch signaling pathway
asthma
type I diabetes mellitus
beta alanine metabolism
inositol phosphate metabolism
NES
F
PC2: 2% variance
PC1: 4% variance
LRC
non-LRC
MRD
untreated

Figure S6, related to Figure 6.
Characterization of cells at minimal residual disease.

(A-B) 10^7 ALL-265 cells were injected into 28 mice; when 40 % of bone marrow cells were human, therapy was started using vincristine (VCR, 0.25 mg/kg; n=4) or cyclophosphamide (Cyclo, 100 mg/kg; n=4) or a combination thereof (VCR+Cyclo; n=12), weekly for 3 weeks; VCR+Cyclo combination treatment had reduced tumor burden to minimal residual disease (MRD; < 1% human cells in bone marrow).
(A) Mean of each group +/- standard error; * p<0.05, **** p<0.0001 by two-tailed unpaired t-test; mice receiving buffer had to be sacrificed after two weeks of treatment due to end stage leukemia.
(B) Percentage of PDX ALL cells in mouse bone marrow as determined by flow cytometry post mortem as mean +/- standard error; * p<0.05, ** p<0.01, **** p<0.0001 by two-tailed unpaired t-test.
(C) To study their behavior after release of treatment pressure, ALL-199 cells were injected into 4 mice per group which were repetitively monitored by in vivo imaging; at substantial tumor burden, mice were treated with Vincristine (VCR) 0.4 mg/kg and left untreated thereafter; mean of each group +/- standard error.
(D-F) ALL-199 cells were injected into 19 mice; when 30 % of bone marrow cells were human, 5 untreated samples were harvested and one mouse were subjected to single cell sequencing; remaining mice received either buffer or vincristine (VCR, 0.25 mg/kg; n=5) or cyclophosphamide (Cyclo, 100 mg/kg; n=3) or a combination thereof (VCR+Cyclo; n=6) weekly for 2 weeks; when VCR+Cyclo combination treatment had reduced tumor burden to minimal residual disease (MRD; < 1% human cells in bone marrow), cells from the 6 VCR+Cyclo mice were isolated and one mouse were subjected to single cell sequencing.
(D) Hierachical clustering and gene expression heatmap across the 500 most differentially expressed genes between MRD cells and untreated cells in ALL-199 single cell RNA sequencing (MRD cells n=90; untreated cells n=32; p < 0.01; for gene annotation see Table S7).
(E) Significantly enriched KEGG pathways (p < 0.05) in MRD cells versus untreated cells as determined by geneset enrichment analysis.
(F) Principle component analysis of transcriptomes of 32 untreated control ALL-199 single cells and 90 MRD cells together with single cell data from LRC and non-LRC as in Figure 5C.

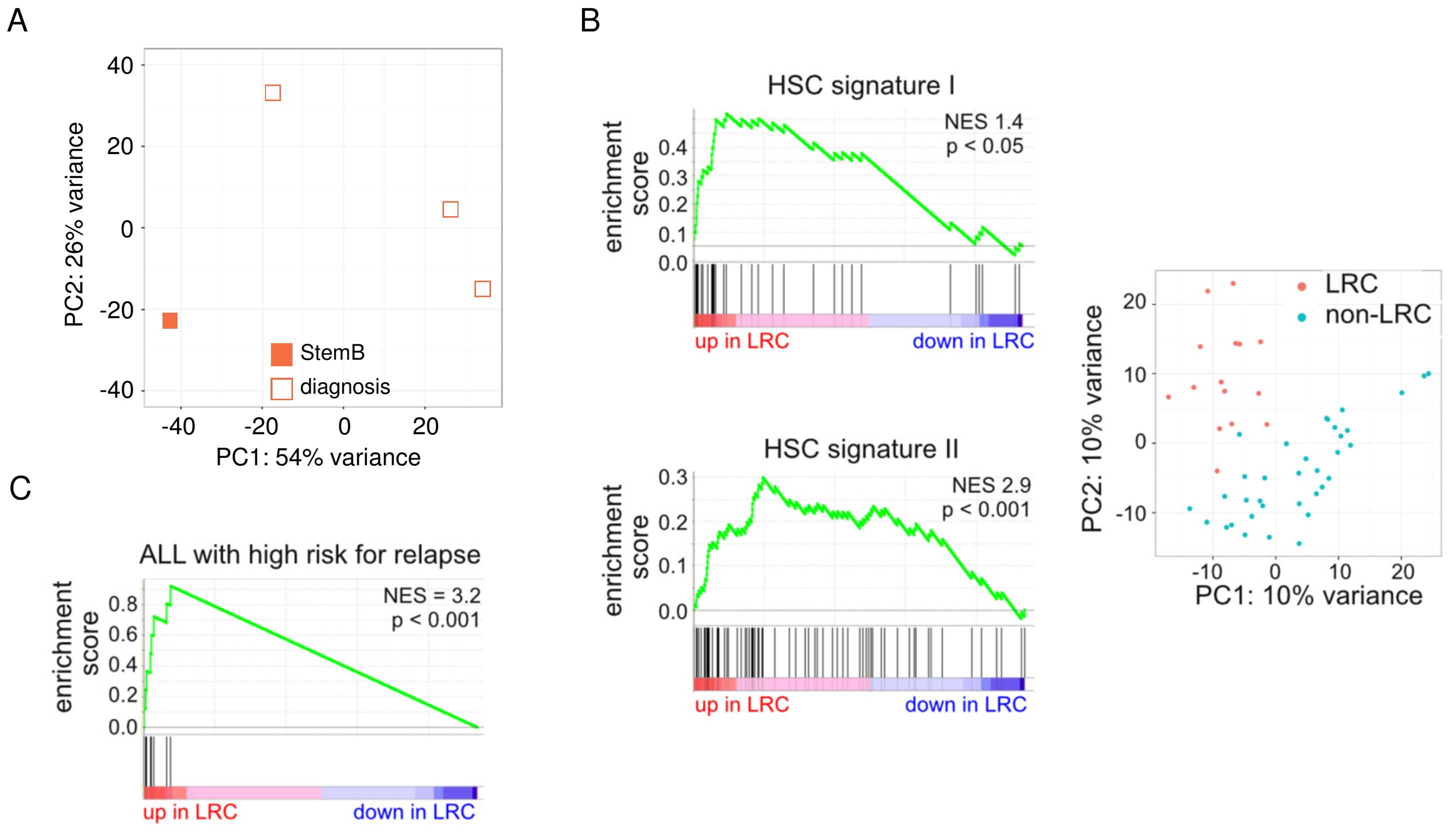

Figure S7, related to Figure 7.
LRC resemble primary MRD cells from patients.

(A) Principal component analysis of the bulk adult StemB sample compared to 3 bulk diagnosis samples of adult patients with BCR-ABL positive ALL.
(B) Gene set enrichment analysis of genes differentially expressed in LRC versus non-LRC and published signatures; HSC signature I = Georgantas et al., 2004; HSC signature II = Eppert et al., 2011 (left panel). Principal component analysis (PCA) of ALL-265 single cells on the basis HSC marker genes (Eppert et al., 2011) (right panel).
(C) Geneset enrichment analysis for a published gene signature prognostic for ALL with high risk of relapse (Kang et al., 2010).

Table S7, related to Figure 7.
Clinical data from BCP ALL patients of transcriptomes at diagnosis and/or MRD.

sample	age#	sex	multi-center study	genetic subtype	flow RG	proto-col RG&	stage after induc-tion II	day of MRD measure-ment §	BM blasts at MRD (%)$	sort
**1**	38	F	GMALL 0703	BCR-ABL	na	VHR	CR	71	0.24	StemB*
**2**	39	M	GMALL 0703	BCR-ABL	na	VHR	CR	71	0.32	StemB*
**1**	4	F	BFM 2009	ETV6/ RUNX1	MR	MR	na	na	na	$CD19^{+}$, $CD10^{+++}$, $CD20^{-}$
**2**	3	F	BFM 2009	ETV6/ RUNX1	MR	SR	na	na	na	$CD19^{+}$, $CD99^{bright}$, $CD10^{+++}$
**3**	5	M	BFM 2009	HD	MR	HR	na	33	0.69	$CD19^{+}$, $CD10^{+}$, $CD123^{+}$
**4**	18	M	BFM 2009	B OTHER	MR	HR	na	33	1.10	$CD19^{+}$, $CD10^{++}$, $CD45^{-/dim}$
**5**	3	F	BFM 2009	HD	MR	MR	na	33	0.13	$CD19^{+}$, $CD10^{++}$, $CD20^{dim}$

#age at diagnosis in years; F=female; M=male; GMALL=German Multicenter ALL Study Group; BFM=Berlin-Frankfurt-Münster; HD=high hyperdiploid karyotype; RG=risk group; na=not applicable; MR=medium risk; VHR=very high risk; SR=standard risk; HR=high risk; &therapy risk group (RG) assignment; §days after onset of treatment; BM=bone marrow; $in BCR-ABL positive samples, MRD was quantified by PCR using the BCR-ABL/ABL ratio; *StemB cells are $CD19^{+}$, $CD34^{+}$, $CD38^{-/low}$ according to Lutz et al., 2013; Hong et al., 2008; Castor et al., 2005

Table S8, related to Figure 7.
List of most significantly differentially expressed genes between primary samples from 5 primary ALL diagnosis and 3 MRD samples after 33 days of treatment.

Provided as an Excel file.

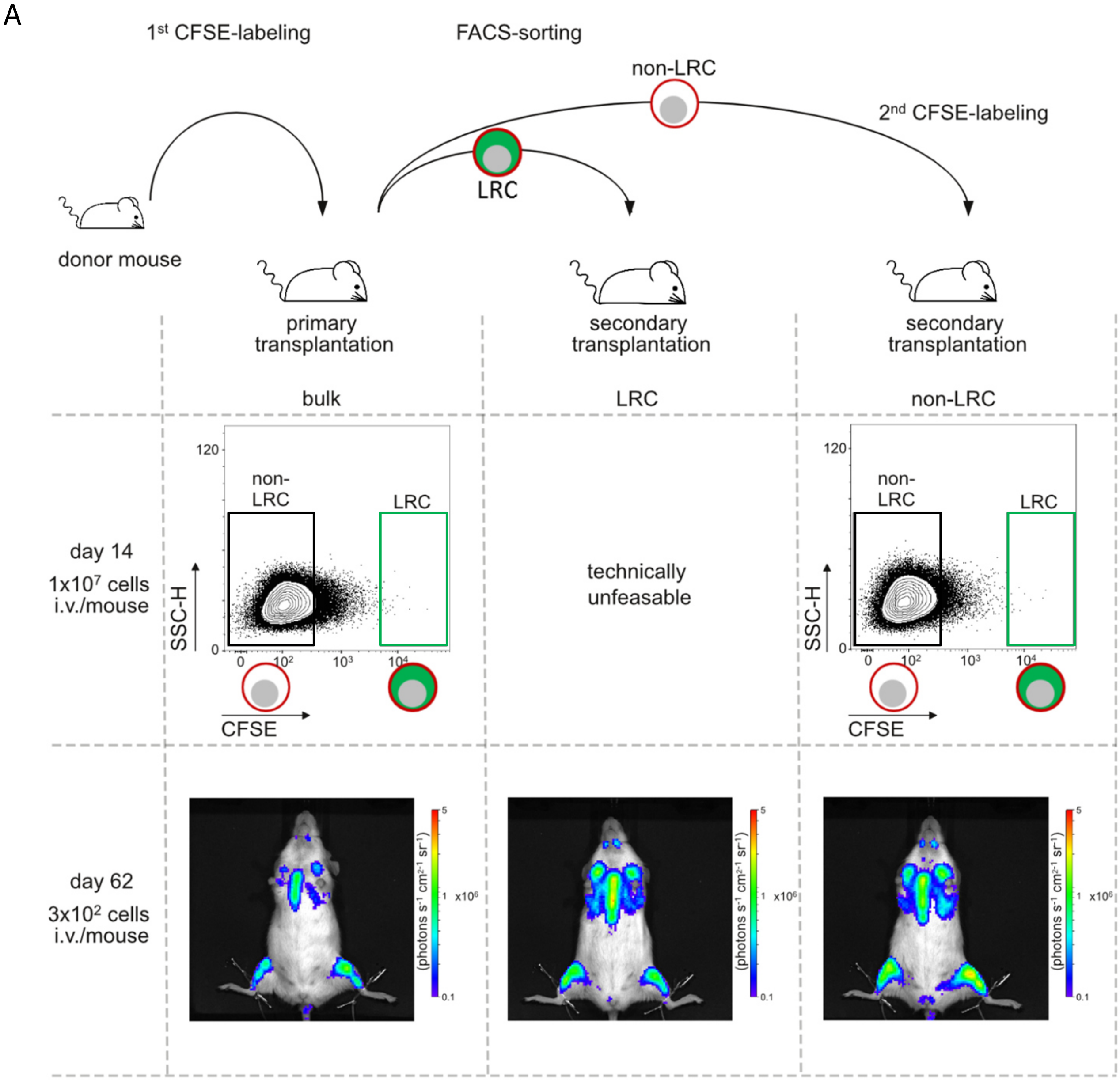

A
1st CFSE-labeling
FACS-sorting
non-LRC
2nd CFSE-labeling
LRC
donor mouse
primary transplantation
secondary transplantation
secondary transplantation
bulk
LRC
non-LRC
day 14
1x10^7 cells i.v./mouse
technically unfeasable
non-LRC
LRC
SSC-H
CFSE
day 62
3x10^2 cells i.v./mouse
(photons s-1 cm-2 sr-1)
x10^6

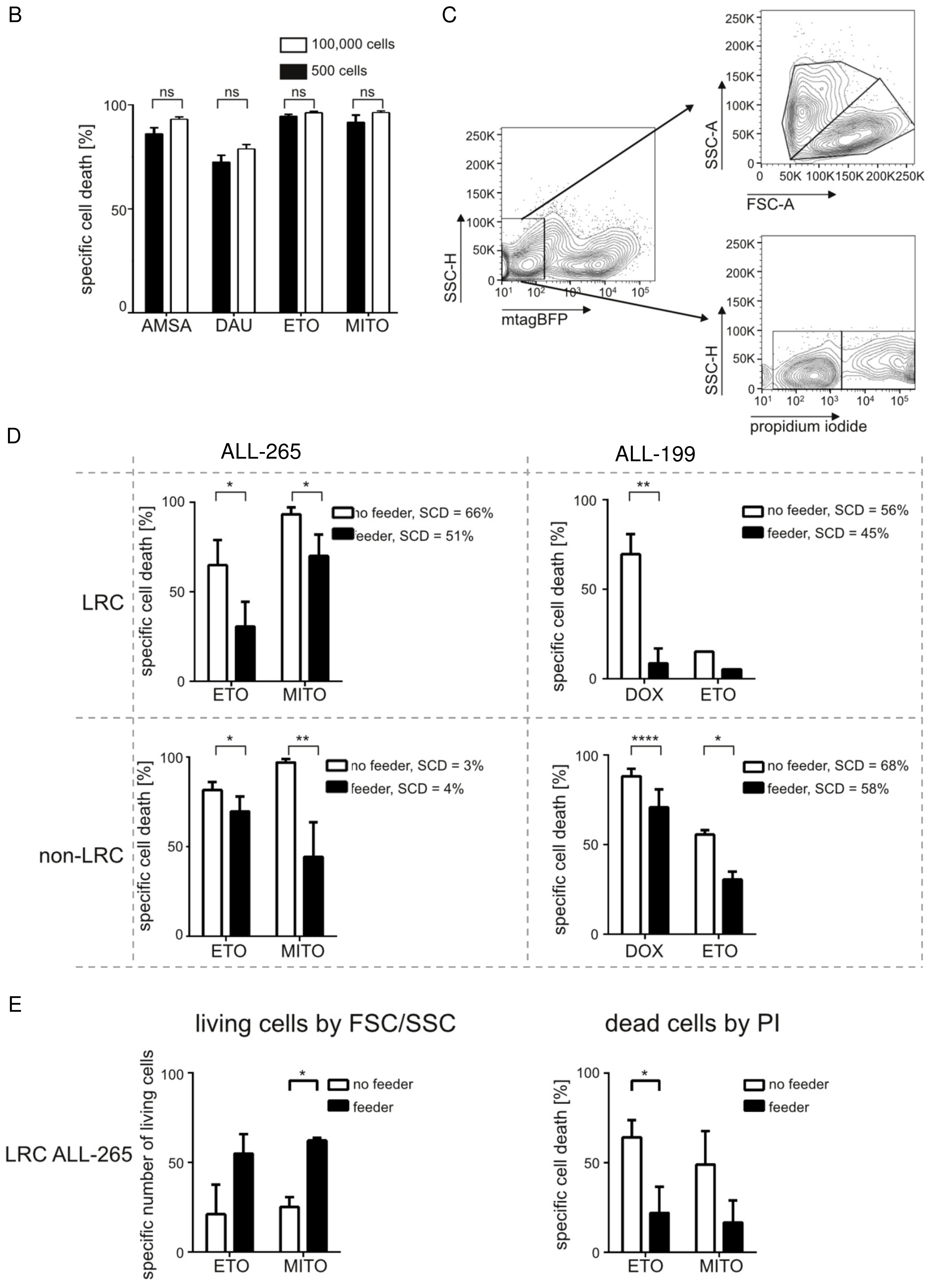
B
100,000 cells
500 cells
ns
specific cell death [%]
100
50
0
AMSA
DAU
ETO
MITO
C
250K
200K
150K
100K
50K
0
SSC-H
mtagBFP
SSC-A
FSC-A
0
50K
100K
150K
200K
250K
propidium iodide
D
ALL-265
ALL-199
LRC
non-LRC
*
**
****
no feeder, SCD = 66%
feeder, SCD = 51%
no feeder, SCD = 56%
feeder, SCD = 45%
no feeder, SCD = 3%
feeder, SCD = 4%
no feeder, SCD = 68%
feeder, SCD = 58%
ETO
MITO
DOX
E
living cells by FSC/SSC
dead cells by PI
LRC ALL-265
specific number of living cells
no feeder
feeder

F

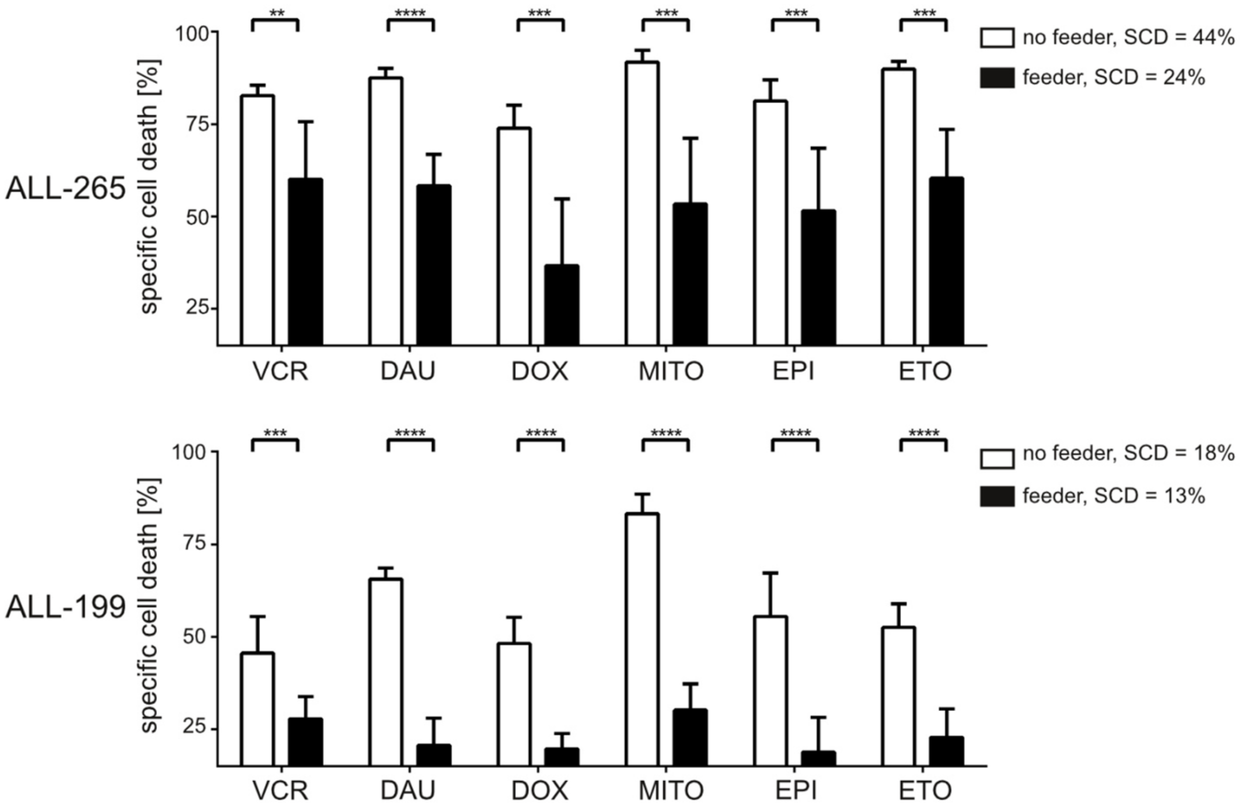
ALL-265
specific cell death [%]
100
75
50
25
**
****
***
***
***
***
VCR
DAU
DOX
MITO
EPI
ETO
no feeder, SCD = 44%
feeder, SCD = 24%
ALL-199
specific cell death [%]
100
75
50
25
***
****
****
****
****
****
VCR
DAU
DOX
MITO
EPI
ETO
no feeder, SCD = 18%
feeder, SCD = 13%

G

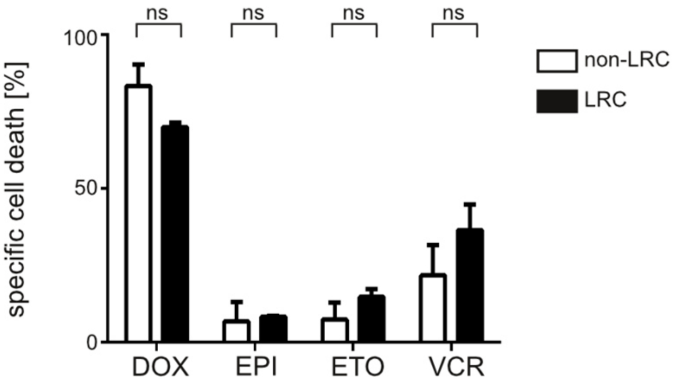
specific cell death [%]
100
50
0
ns
ns
ns
ns
DOX
EPI
ETO
VCR
non-LRC
LRC

Figure S8, related to Figure 7.
Identical growth behavior upon re-transplantation and identical ex vivo drug sensitivity in LRC versus non-LRC.

(A) Upper panel shows experimental procedure; ALL-265 cells were amplified in donor mice, CFSE labeled, re-transplanted into primary recipients and re-isolated after 14 days (left lane). Cells were separated into LRC (middle lane) and non-LRC (right lane) and re-transplanted into secondary recipients which were imaged after 62 days (lower row). non-LRC were additionally re-labeled with CFSE and re-transplanted at high numbers which was unfeasible for LRC due to their minor abundance (middle panel).

(B) 500 or 100,000 freshly isolated non-LRC (ALL-265) were stimulated ex vivo for 48 hours with the following cytotoxic drugs: amsacrine (AMSA; 18 µM), daunorubicine (DAU; 250 nM); etoposide (ETO; 30 µM) and mitoxantrone (MITO; 675 nM); shown is one experiment in triplicates +/- standard error; ns = not significant by two-tailed unpaired t-test. Specific cell death was determined by DAPI staining and specific cell death calculated thereof.

(C-F) Freshly isolated PDX cells were seeded in triplicates in the presence or absence of irradiated MS-5 cells expressing the blue fluorochrome mtagBFP. Cells were stimulated for 48-72 hours and all cells per well were removed by trypsin digestion and analyzed by flow cytometry. * $p<0.05$, ** $p<0.01$, *** $p<0.001$ and **** $p<0.0001$ by two-tailed unpaired t-test. SCD = spontaneous cell death in the absence of cytotoxic drugs.

(C) Feeder cells were excluded by gating on non-blue/mtagBFP-expressing cells; living cells were quantified in absolute and relative amounts using either forward/side scatter analysis or propidium iodide staining with similar results.

(D, E) 700-1,900 fresh LRC or non-LRC were stimulated with the following drugs: Etoposide (ETO; 3 µM) or mitoxantrone (MITO; 0.45 µM) for ALL-265 and etoposide (ETO; 15 µM) or doxorubicine (DOX; 0.5 µM) for ALL-199. E shows results obtained by forward sideward scatter analysis, F shows results obtained by propidium iodide (PI) staining as well as absolute number of surviving cells as estimated in forward/side scatter analysis. Shown are mean of up to 3 independent experiments; +/- standard error.

(F) 100,000 unsorted PDX ALL-265 were stimulated with vincristine (VCR; 0.3 µM), daunorubicine (DAU; 0.25 µM), doxorubicine (DOX; 0.5 µM), mitoxantrone (MITO; 0.45 µM), epirubicine (EPI; 0.4 µM) and etoposide (ETO; 3 µM) for 48 hours; 100,000 unsorted PDX ALL-199 were stimulated with vincristine (VCR; 0.03 µM), daunorubicine (DAU; 0.25 µM), doxorubicine (DOX; 0.5 µM), mitoxantrone (MITO; 0.25 µM), epirubicine (EPI; 0.4 µM) and etoposide (ETO; 3 µM) for 72 hours; mean of 9 data points from 3 independent experiments in triplicates is shown; +/- standard error; Welch's correction war required in two-tailed unpaired t-test for ALL-265 and DAU stimulation in ALL-199.

(G) 14 days after transplantation, LRC or non-LRC were isolated and 500-800 cells stimulated ex vivo for 48 hours with the following cytotoxic drugs: doxorubicine (DOX; 500 nM), epirubicine (EPI; 500 nM); etoposide (ETO; 30 µM) and vincristine (VCR; 300 nM). Specific cell death was determined after 48h by forward-side scatter and by DAPI staining and specific cell death calculated thereof; mean of 6 data points from 2 independent experiments in triplicates is shown +/- standard error; ns = not significant by two-tailed unpaired t-test.

Supplemental Experimental Procedures

The NSG mouse model of individual ALL

ALL blasts were obtained from children and adults treated within clinical multicenter studies. NSG mice (NOD/scid, IL2 receptor gamma chain knockout mice) were obtained from The Jackson Laboratory (Lund, Sweden). The animal model was performed as described (Liem et al., 2004). Briefly, fresh primary ALL cells were isolated by Ficoll gradient centrifugation from peripheral blood or bone marrow aspirates that had been obtained from leftovers of clinical routine sampling before onset of therapy. 10 million ALL cells were injected into 6-12 weeks old NSG mice via the tail vein. Engraftment was monitored by 2-weekly flow cytometry measurement of human cells in peripheral blood starting at week 6. ALL-265 was first engrafted by Jean Pierre Bourquin and Beat Bornhäuser in Zurich. Mice were sacrificed at first clinical signs of disease, as measured by quantification of human cells in peripheral blood or by in vivo imaging. From engrafted mice, PDX ALL cells were harvested from enlarged spleens and either directly re-injected or frozen at -190 °C and re-injected after thawing. Accuracy of sample identity was verified by repetitive finger printing using PCR of mitochondrial DNA (Hutter et al., 2004).

Cloning

The construct encoding for all 3 transgenes (Figure S1A) was generated by cloning a synthesized DNA-fragment (Eurofins Scientific, Luxembourg) encoding for mKate and a truncated form of the human nerve growth factor receptor lacking any intracellular signaling domain (NGFR; construct -mKateT2A-NGFR) into the pCDH-EF1α-extGLucT2A-copGFP Vector (Terziyska et al., 2012), leaving membrane anchored Gaussia luciferase and replacing copGFP gene using BamHI and SalI; T2A or P2A self-cleaving peptides enabled equimolar expression of the transgenes. For immunohistochemistry, cells were additionally transduced with a construct expressing mCherry which was obtained by amplifying mCherry from the pSicoR-U6-EF1a-mCherry Vector (addgene, Cambridge, MA, USA) and cloning it into the pCDH-EF1α-extGluc-T2A-copGFP Vector replacing the copGFP gene using BamHI and SalI.

Lentiviral transduction of ALL PDX cells and enrichment of transgenic cells

ALL-199 and ALL-265 were transduced using pCDH-EF1α-extGlucT2A-mKate-NGFR. Third generation packaging plasmids pMDLg/pRRE, pRSV-Rev and pMD2-G (Dull et al., 1998) were kindly provided by T. Schroeder. High-titer vesicular stomatitis virus (VSV) G protein-pseudotyped lentivector was prepared by transient 4-plasmid transfection of 293T cells using TurboFect Transfection Reagent (Thermo Scientific, Waltham, MA, USA) and supernatant concentration as described (Klier et al., 2008; Terziyska et al., 2012). The functional titer of virus was determined by transduction of NALM-6 B-ALL cell line cells with serial dilutions of the vector stock, followed by analysis of transgene positive cells using flow cytometry.

Generation of transgenic PDX cells was performed as previously described (Terziyska et al., 2012). In brief, PDX cells were transduced over night with lentivirus at MOI > 10 in the presence of 8 µg/ml polybrene. The next day, cells were washed thoroughly and injected into mice. After passaging, cells expressing the transgenes were enriched in two consecutive rounds by flow cytometry using FACSAriaIII (BD Biosciences) and gating on the red fluorochrome before cell re-amplification in mice. Although lentiviral transduction could in principle alter cells due to the transduction process or genomic integration, we could not detect adverse effects so far in comprehensive quality controls (Terziyska et al., 2012).

Bioluminescence in vivo imaging

For bioluminescence in vivo imaging mice were anesthetized with isoflurane and D-Luciferin (BIOMOL GmbH, Hamburg, Germany) dissolved in sterile PBS was used as substrate. Immediately after intravenous tail vein injection of 150 mg/kg of native D-Luciferin per mouse, mice were imaged for 30 seconds or up to 2 minutes using a field of view of 12.5 cm with binning 8, f/stop 1 and open filter setting using the IVIS Lumina II Imaging System (Perkin Elmer, MA, USA). The Living Image software 4.x (Perkin Elmer, MA, USA) was used for data acquisition and quantification of light emission using a scale with a minimum of 1.8×10^4 photons per second per cm^2 per solid angle of 1 steradian (sr) (Terziyska et al., 2012). Mice were considered positive for engraftment, if light emission by the entire mouse exceeded $5x10^5$ photons s^{-1} and positive signals were detected at typical sites at the lower extremities.

Reagents

For flow cytometry, analysis of NGFR, mKate, mCherry, BrdU, Annexin V, DAPI and PI was performed by flow cytometry, using BD LSRFortessa and BD FACSAriaIII (BD Biosciences, Heidelberg, Germany). The following antibodies were used: NGFR-PerCP-Cy5.5 (Biolegend, CA, USA), BrdU-APC, Annexin V-FITC detection kit (both

from BD Biosciences, Heidelberg, Germany). Mouse CD45-APC-Cy7 (Biolegend, San Diego, CA, USA) was used to exclude mouse cells.
BrdU incorporation was detected using the BrdU Flow Kit (BD Biosciences, Heidelberg, Germany). For analysis of cell viability, DAPI and/or PI were added to the cells at a concentration of 1µg/ml. All antibodies and reagents were used according to the manufacturer's instructions.
For chemotherapy treatments in vivo and ex vivo vincristine (VCR; Merck, Darmstadt, Germany), etoposide (ETO; Sigma Aldrich, St. Louis, USA), cyclophosphamide (Cyclo; Baxter, USA), epirubicine (EPI; Sigma Aldrich, St. Louis, USA), amsacrine (Amsa, Sigma Aldrich, St. Louis, USA), cytarabine (Ara-C; cell pharm GmbH, Bad Vilbel, Germany), daunorubicin (DAU; Sigma Aldrich, St. Louis, USA), mitoxantrone (MITO; Sigma Aldrich, St. Louis, USA) or doxorubicin (DOX, Sigma Aldrich, St. Louis, USA) were used.

Labeling of PDX cells with BrdU and CFSE

To label PDX cells with BrdU, donor mice were fed with BrdU (VWR, Radnor, PA, USA) during the 7 last days before cell isolation, at approximately 0.8 mg/kg/d BrdU using BrdU-containing drinking water. Freshly isolated PDX cells were labeled with CFDASE (Life Technologies, Carlsbad, CA, USA) according to manufacturer's instructions. Cells were washed and directly injected into recipient mice. The procedures resulted in both BrdU and CFSE positivity of well above 98% of PDX cells, as validated by flow cytometry. As PDX ALL cells are heterogeneous in size, loss of CFSE appears as continuum in flow cytometry devoid of the distinct peaks known from normal lymphocytes.

Enriching human PDX ALL cells from murine bone marrow

The aim was to isolate and enrich minute numbers of human PDX ALL cells out of a major excess of murine bone marrow cells. The procedure was designed according to published protocols for isolating normal mouse hematopoietic stem cells from murine bone marrow (Takizawa et al., 2011). Our studies concentrated on the first 3 weeks of ALL growth in mice, when low tumor burden is mainly restricted to bone marrow without major involvement of further organs (data not shown).

<u>Isolation of bone marrow cells from mice</u>

To collect as many bone marrow cells as possible from each mouse, the hip, femura, tibiae, spine and sternum were isolated and crushed in a porcelain mortar. The suspension was washed with cold PBS, filtered through a 70 µm cell strainer, washed again with PBS and re-suspended in cold PBS at $1x10^7$ cells/ml.

<u>Step 1: Enriching NGFR expressing PDX cells from the bone marrow suspension</u>

A first enrichment step consisted in magnetic cell separation (MACS) of NGFR-expressing PDX ALL cells from the entire mouse bone marrow isolated. 20 µl per $1x10^7$ cells of anti-human NGFR MicroBeads (Miltenyi Biotech, Bergisch Gladbach, Germany) were added to the isolated mouse bone marrow cell suspension and incubated 10 minutes at 4°C. A maximum of $2x10^8$ cells were loaded onto a LS column (Miltenyi Biotech, Bergisch Gladbach, Germany), prepared according to manufacturer's instructions. Cells were recovered from the column according to manufacturer's instructions and washed with PBS.

<u>Step 2: Enriching and quantifying fluorochrome expressing PDX cells from NGFR-expressing cells</u>

The second consecutive enrichment step consisted in flow cytometry enrichment of red fluorochrome expressing cells out of the cell suspension obtained after MACS enrichment. Cells obtained after MACS enrichment were stained with DAPI to exclude dead cells and with anti-muCD45-APC-Cy7 (anti-mouse CD45) to exclude murine hematopoietic cells. Cells were quantified and sorted using a BD FACSAriaIII (BD Biosciences, Heidelberg, Germany), gating (i) on the lymphocyte gate in forward/side scatter, (ii) the negative gate for both mouse CD45 and DAPI and ultimately (iii) the positive gate for the red fluorochrome.
Alternatively and to quality control for the MACS enrichment step, 10% of the entire population of bone marrow cells was directly analyzed by flow cytometry without prior MACS enrichment and with the identical staining procedure (Figure S1D). The disadvantage of this procedure lies in the prolonged periods of time required for flow cytometric cell enrichment disabling measuring more than 10% of all cells.

Enriching dormant cells (LRC) from human PDX ALL cells

<u>Step 3: Separating PDX ALL cells into LRC and non-LRC</u>

Separating PDX ALL cells into LRC and non-LRC was performed within the flow cytometry enrichment step described above (Step 2) by addition of a 4th gating strategy. Additionally to gating on (i) the lymphocyte gate in

forward/side scatter, (ii) the negative gate for both mouse CD45 and DAPI and (iii) the positive gate for the red fluorochrome, gating (iv) on CFSE was used to discriminate LRC and non-LRC as shown in Figure 1D. To set gate 4, CFSE intensity was measured at day 3 after injection when major bleaching had stopped; maximum CFSE MFI was used to define start of any cell proliferation ("0 divisions"). Maximum CFSE MFI was divided by factor 2 to calculate CFSE bisections mimicking cell divisions. 7 CFSE MFI bisections or more were defined as entire loss of the CFSE signal characterizing non-LRC. The LRC gate was set to include all cells harboring high CFSE signal of below 3 bisections of the maximum CFSE MFI (Schillert et al., 2013) (Figure 1D). All further analyses were done and analyzed with the same instrument settings and gates as determined using the sample on day 3 sample of the experiment.

Ex vivo culture of PDX cells

PDX cells were cultured in RPMI medium supplemented with 20% FSC, 1% pen/strep, 1% gentamycin, 6 mg/l insulin, 3 mg/l transferrin, 4 µg/l selenium (ITS-G, Gibco, San Diego, CA, USA), 2 mM glutamine, 1 mM sodium pyruvate, 50 µM α-thioglycerol (Sigma-Aldrich, St. Louis, MO, USA).

Limiting dilution transplantation assay (LDTA)

For LDTAs, NSG mice were injected intravenously with different amounts of PDX cells from ALL-265 or ALL-199. Development of leukemia was monitored by bioluminescence in vivo imaging every 7 to 14 days after cell injection. LIC frequencies were determined according to Poisson statistics, using the ELDA software application (http://bioinf.wehi.edu.au/software/elda/) (Hu and Smyth, 2009).

Drug stimulation ex vivo

500 LRC and 500 or 100,000 non-LRC were cultured in 100 µl medium in 96-well plates, in cell concentrations of 5,000 cells/ml or 10^6 cells/ml. Cytotoxic drugs were added in triplicates at the clinically relevant concentrations described in each Figure legend. Cell death was measured after 48h by forward-side scatter and DAPI or propidium iodide staining in a flow cytometer. Specific cell death induced by each drug was calculated as follows: specific cell death = [(cell death(stimulated) – cell death(control) / (100 – cell death(control))] * 100.

For co-cultures, MS-5 cells stably expressing mtagBFP as blue fluorochrome were irradiated in suspension with 60 Gy and seeded at 10^4 per well in a 96 well plate; 700 - 1,900 freshly isolated PDX cells were incubated with and without feeder cells in 100 µl medium for 24-48h stimulated with conventional cytotoxic drugs at clinically relevant concentrations; entirely all cells of each well were removed using trypsin digestion and stained with propidium iodide; feeder cells were excluded by gating on non-blue-expressing cells independently from CFSE or propidium iodide staining; absolute numbers of living PDX cells were measured using forwardside scatter analysis and cell death was additionally measured by propidium iodide staining in flow cytometry.

In vivo treatment of mice

For treatment of LRC, NSG mice were injected i.v. with $1x10^7$ PDX cells. 7 days after cell injection, control animals received physiological salt solution i.p., while treatment group mice were injected with chemotherapeutic drugs as indicated in Figure legends. Mice were taken down 3 days later, bone marrow was collected, and PDX cells were isolated and analyzed for CFSE label retention. For calculation of relative drug effect on LRC compared to non-LRC (Figure 4D), first absolute number of control LRC or non-LRC were divided by the absolute number of treated LRC or non-LRC, respectively. In a second step, relative cell reduction in non-LRC was set to 100% and cell reduction in LRC was calculated relative to non-LRC. A maximum of 4 animals could be included into the same experiment as a maximum of 4 animals could be analyzed for CFSE distribution at the same day.

To obtain cells at minimal residual disease, $1x10^6$ ALL-199 or ALL-265 were injected into 19 NSG mice and leukemic growth was followed by weekly in vivo imaging. Treatment was started at an average of $1x10^{11}$ photons s^{-1}, when untreated cells were recovered from 5 mice. Mice were divided into different treatment groups which were treated as indicated in Figures legends.

Immunostaining of bone marrow sections

Mouse femurs were fixed in zinc formalin fixative for 1 day at 4°C. Bones were washed with PBS and decalcified with Osteosoft (Merck) for 3 days at 4°C, infiltrated with 30% sucrose for 1 day at 4°C, embedded in O.C.T. compound (Sakura) and stored at -80°C. Cryosections of decalcified bones were obtained by using the CryoJane tape transfer system (Leica). For immunostaining, sections were permeabilized and blocked with 5% goat serum and 0.1% Tween-20 serum in PBS for 45 min at room temperature. Primary antibodies were applied for 1 day at 4°C and

followed by secondary antibody incubation for 45 min at room temperature. Sections were finally stained with 10 mg/ml DAPI for 15 min and the slides were mounted with prolong gold (Invitrogen). Washing in between each staining steps was performed. Primary antibodies were rabbit-anti-FITC (ThermoFisher; 1:100) and rabbit-anti-mCherry (Abcam; 1:100) and goat-anti-rabbit with Alexa 594 (Invitrogen) was used as secondary antibody. Images were acquired on a Leica SP5 confocal microscope and analyzed with ImageJ. CFSE signal intensity was adapted to the mCherry signal by adjusting the 8 bit threshold for quantification of the LRC population based on FACS data. The endosteal region was defined as less than 100 µm from bone matrix (Nombela-Arrieta et al., 2013). Cells of interests were counted semi-automatically by the program ImageJ. Relative endosteal cells were calculated as absolute cell numbers in the endosteal region divided by absolute cell numbers in entire bone marrow section. Mean and standard error were calculated from at least 3 sections of each femur from 2 independent mice.
For immunhistology of primary bone marrow biopsies, bone marrow biopsies were fixed and stained using the avidin-biotin-peroxidase complex (ABC) method (Hsu et al., 1981) and anti-TdT antibody (Leica, Germany) and anti-Ki-67 antibody (Dako, Germany).

Flow cytometric cell enrichment of StemB cells from BCR-ABL positive ALL
Thawed mononuclear bone marrow cells were handled on ice and stained with CD3-FITC, CD19-PE, CD34-APC, CD38-PECy7 (all Becton Dickinson) and DAPI 0.1 µg/ml; StemB cells expressing $CD3^-$ $CD34^+$ $CD38^{-/low}$ $CD19^+$ cells were enriched using the FACSAria™ (Becton Dickinson) according to (Castor et al., 2005; Hong et al., 2008; Lutz et al., 2013).

Flow cytometric cell enrichment of diagnostic and MRD pediatric BCP-ALL cells
Thawed mononuclear bone marrow cells were handled on ice and stained using antibodies appropriate for minimal residual disease (MRD) detection against CD10, CD19, CD20, CD34, CD38, CD45, CD58, CD99, and CD123. Leukemic blasts were enriched to >95% purity using a FACSAria™ Fusion cell sorter equipped with an automatic cell deposition unit (ACDU; Becton Dickinson); data analysis was performed using the FACSDiva™ software (Becton Dickinson).

Bulk RNA sequencing library construction
PDX LRC and non-LRC cell populations were sorted into lysis buffer composed of 0.2 % Triton X-100 (Sigma) and 2 U/µl of RNAse Inhibitor (Life Technologies). ERCC spike-in controls (Life Technologies) were added to the cell lysis mix at 1:5,000 dilution. RNA was cleaned-up from the crude lysate with Agencourt RNAclean XP SPRI beads (Beckman-Coulter). cDNA was synthesized and pre-amplified from 5 µl of lysate according to the Smart-seq2 protocol (Picelli et al., 2013).
For each pediatric ALL MRD and PDX MRD sample, 2000 cells were sorted into TCL buffer (Qiagen). RNA was cleaned up using Agencourt RNAclean XP SPRI beads from half of the lysate and used to generate UMI-seq libraries as previously described (Parekh et al., 2016).
For all libraries, 1 ng of pre-amplified cDNA was used as input for tagmentation by the Nextera XT Sample Preparation Kit (Illumina), where a second amplification round was performed for 12 cycles.

RNA sequencing library construction of primary StemB single cells
Single adult StemB cells were deposited in 96-well plates containing 5 µl lysis buffer composed of a 1:500 dilution of Phusion HF buffer (NEB). Single-cell RNA-seq libraries were constructed using the SCRB-seq method according to (Soumillon et al., 2014).

RNA-seq analysis
All sequencing reads were demultiplexed from the Nextera (i5 and i7) indices.
For Smart-seq libraries, demultiplexed reads were aligned to the human genome (hg19) and ERCC reference using NextGenMap (Sedlazeck et al., 2013). Count data was generated from mapped reads using featureCounts (Liao et al., 2014) on ENSEMBL gene models (GRCh38.74).
For UMI-seq and SCRB-seq libraries, read pairs were processed by tagging the cDNA read with barcode and UMI sequences using the Drop-seq tools pipeline (Macosko et al., 2015). Tagged reads were aligned to the human genome (hg19) using STAR (Dobin et al., 2013) and sample-wise count tables generated using Drop-seq tools.

To remove noise from lowly expressed genes, count data sets were subjected to data-driven gene filtering using the HTSFilter R package (Rau et al., 2013). For PDX single cell sequencing libraries, only those cell data sets were used

which came from viable cells, obtained at least 1 million reads and detected at least 3000 genes (TPM > 1). For combined bulk (1x ALL-265; 4x ALL-265) and single cell (1x ALL-265) analysis (Figure 5), filtered single cell datasets were included summarized by gene-wise median read count as one LRC and non-LRC replicate. Differential expression (DE) analysis was done in the DESeq2 R package (Love et al., 2014) using the Wald test.
A combined LRC signature (ALL-265 & ALL-199; 250 genes; FC > 1; padj < 0.05) was obtained from this data.
Overrepresentation of significantly differentially expressed genes in KEGG pathways was tested by a fixed network enrichment analysis (FNEA) implemented in the neaGUI R package (Alexeyenko et al., 2012).
We applied hierarchical clustering gene-wise and sample-wise with complete linkage based on Euclidian distances of variance stabilized counts of DE genes (500 genes with lowest padj, FDR adjustment (Benjamini and Hochberg, 1995)) and plotted as heatmap. The reference expression value is the expression average of non-LRC cells.
Principal Component Analysis (PCA) of LRC PDX cells was performed on variance stabilized counts of the 500 most variable genes to display the main variance of the samples.
To analyse combined data from all obtained single-cells, count data was normalized accounting for batch effects using SCONE (Risso et al., 2014). PCA and k-means clustering of combined single-cell data was performed on all shared detected genes.
Gene set enrichment analysis was performed using GSEA Desktop Application. For ranking all genes, a metric score was calculated by multiplying their log fold changes with the –log10(p_adj) values and submitted to the Pre-Ranked GSEA tool. The statistical significance was determined by 1000 gene set per mutations (Subramanian et al., 2005).

Dynamical modelling

The growth behavior of ALL cells in bone marrow has been analyzed using mechanistic ordinary differential equation models describing the population growth and the CFSE dilution. To gain insights into the in vivo growth behavior of ALL cells, we compared three alternative models. The first model assumed exponential growth, the second model assumed logistic growth caused by a decreased rate of cell division at higher cell densities, and the third model assumed logistic growth caused by an increased rate of cell death at higher cell densities.
The state variables of all three models are the cell number $n(t)$ and the mean fluorescence intensity $m(t)$. The governing equations for $n(t)$ and $m(t)$,

$$dn/dt = (\alpha(n)-\beta(n))\, n, \qquad N(0) = n_0,$$
$$dm/dt = -\,(\alpha(n)+k)\, m, \qquad m(0) = m_0,$$

have been deduced from existing partial differential equation models (Hasenauer et al., 2012). In this governing equations $\alpha(n)$ denotes the rate of cell division, $\beta(n)$ denotes the rate of cell death, and k denotes the rate of CFSE degradation.
The three model alternatives only differed in the parameterization of rates of cell division and cell death, $\alpha(n)$ and $\beta(n)$. For the exponential growth model all rates were constant, $\alpha(n) = \alpha_0$ and $\beta(n) = \beta_0$. For the logistic growth model with decreasing cell division at higher cell densities $\alpha(n) = \alpha_0\,(1\text{-}n/n_\alpha)$ and constant $\beta(n) = \beta_0$ were used. For the logistic growth model with increasing cell death at higher cell densities constant $\alpha(n) = \alpha_0$ and $\beta(n) = \beta_0\,(1+n/n_\beta)$ were used. As the measurement of the mean intensity induced by CFSE is corrupted by the cell's autofluorescence, we measure $m'(t) = m + m_a$, in which m_a denotes the average autofluorescence.
The parameters of the three models were determined from measurement of $n(t)$ and $m'(t)$ using maximum likelihood estimation, assuming normally distributed measurement noise. For model comparison the Akaike information criterion (AIC) was used.

Statistics

All statistical analyses were calculated using GraphPad Prism 6 software. Two-tailed unpaired t-test was applied to evaluate differences after drug treatment. F-test was applied to compare standard deviations; in cases, when standard deviations differed significantly, Welch's correction was applied. LIC frequencies were calculated according to Poisson statistics using the ELDA software application (http://bioinf.wehi.edu.au/software/elda) (Hu and Smyth, 2009).

Supplemental References

Alexeyenko, A., Lee, W., Pernemalm, M., Guegan, J., Dessen, P., Lazar, V., Lehtio, J., and Pawitan, Y. (2012). Network enrichment analysis: extension of gene-set enrichment analysis to gene networks. BMC Bioinformatics *13*, 226.

Benjamini, Y., and Hochberg, Y. (1995). Controlling the False Discovery Rate: A Practical and Powerful Approach to Multiple Testing. Journal of the Royal Statistical Society Series B, Methodological *57*, 289-300.

Castor, A., Nilsson, L., Astrand-Grundstrom, I., Buitenhuis, M., Ramirez, C., Anderson, K., Strombeck, B., Garwicz, S., Bekassy, A. N., Schmiegelow, K.*, et al.* (2005). Distinct patterns of hematopoietic stem cell involvement in acute lymphoblastic leukemia. Nat Med *11*, 630-637.

Dobin, A., Davis, C. A., Schlesinger, F., Drenkow, J., Zaleski, C., Jha, S., Batut, P., Chaisson, M., and Gingeras, T. R. (2013). STAR: ultrafast universal RNA-seq aligner. Bioinformatics *29*, 15-21.

Dull, T., Zufferey, R., Kelly, M., Mandel, R. J., Nguyen, M., Trono, D., and Naldini, L. (1998). A Third-Generation Lentivirus Vector with a Conditional Packaging System. Journal of Virology *72*, 8463-8471.

Hasenauer, J., Schittler, D., and Allgöwer, F. (2012). Analysis and Simulation of Division- and Label-Structured Population Models. Bull Math Biol *74*, 2692-2732.

Hsu, S. M., Raine, L., and Fanger, H. (1981). Use of avidin-biotin-peroxidase complex (ABC) in immunoperoxidase techniques: a comparison between ABC and unlabeled antibody (PAP) procedures. J Histochem Cytochem *29*, 577-580.

Hu, Y., and Smyth, G. K. (2009). ELDA: Extreme limiting dilution analysis for comparing depleted and enriched populations in stem cell and other assays. J Immunol Methods *347*, 70-78.

Hutter, G., Nickenig, C., Garritsen, H., Hellenkamp, F., Hoerning, A., Hiddemann, W., and Dreyling, M. (2004). Use of polymorphisms in the noncoding region of the human mitochondrial genome to identify potential contamination of human leukemia-lymphoma cell lines. Hematol J *5*, 61-68.

Klier, M., Anastasov, N., Hermann, A., Meindl, T., Angermeier, D., Raffeld, M., Fend, F., and Quintanilla-Martinez, L. (2008). Specific lentiviral shRNA-mediated knockdown of cyclin D1 in mantle cell lymphoma has minimal effects on cell survival and reveals a regulatory circuit with cyclin D2. Leukemia *22*, 2097-2105.

Liao, Y., Smyth, G. K., and Shi, W. (2014). featureCounts: an efficient general purpose program for assigning sequence reads to genomic features. Bioinformatics *30*, 923-930.

Liem, N. L. M., Papa, R. A., Milross, C. G., Schmid, M. A., Tajbakhsh, M., Choi, S., Ramirez, C. D., Rice, A. M., Haber, M., Norris, M. D.*, et al.* (2004). Characterization of childhood acute lymphoblastic leukemia xenograft models for the preclinical evaluation of new therapies. Blood *103*, 3905-3914.

Love, M. I., Huber, W., and Anders, S. (2014). Moderated estimation of fold change and dispersion for RNA-seq data with DESeq2. Genome Biol *15*, 550.

Macosko, E. Z., Basu, A., Satija, R., Nemesh, J., Shekhar, K., Goldman, M., Tirosh, I., Bialas, A. R., Kamitaki, N., Martersteck, E. M.*, et al.* (2015). Highly Parallel Genome-wide Expression Profiling of Individual Cells Using Nanoliter Droplets. Cell *161*, 1202-1214.

Nombela-Arrieta, C., Pivarnik, G., Winkel, B., Canty, K. J., Harley, B., Mahoney, J. E., Park, S. Y., Lu, J., Protopopov, A., and Silberstein, L. E. (2013). Quantitative imaging of haematopoietic stem and progenitor cell localization and hypoxic status in the bone marrow microenvironment. Nat Cell Biol *15*, 533-543.

Parekh, S., Ziegenhain, C., Vieth, B., Enard, W., and Hellmann, I. (2016). The impact of amplification on differential expression analyses by RNA-seq. Scientific reports *6*, 25533.

Picelli, S., Bjorklund, A. K., Faridani, O. R., Sagasser, S., Winberg, G., and Sandberg, R. (2013). Smart-seq2 for sensitive full-length transcriptome profiling in single cells. Nat Methods *10*, 1096-1098.

Rau, A., Gallopin, M., Celeux, G., and Jaffrezic, F. (2013). Data-based filtering for replicated high-throughput transcriptome sequencing experiments. Bioinformatics *29*, 2146-2152.

Risso, D., Ngai, J., Speed, T. P., and Dudoit, S. (2014). Normalization of RNA-seq data using factor analysis of control genes or samples. Nat Biotechnol *32*, 896-902.

Sedlazeck, F. J., Rescheneder, P., and von Haeseler, A. (2013). NextGenMap: fast and accurate read mapping in highly polymorphic genomes. Bioinformatics *29*, 2790-2791.

Soumillon, M., Cacchiarelli, D., Semrau, S., Oudenaarden, A. v., and Mikkelsen, T. S. (2014). Characterization of directed differentiation by high-throughput single-cell RNA-Seq. bioRxiv.

Subramanian, A., Tamayo, P., Mootha, V. K., Mukherjee, S., Ebert, B. L., Gillette, M. A., Paulovich, A., Pomeroy, S. L., Golub, T. R., Lander, E. S., and Mesirov, J. P. (2005). Gene set enrichment analysis: a knowledge-based approach for interpreting genome-wide expression profiles. Proc Natl Acad Sci U S A *102*, 15545-15550.
